# Microtubule-Mediated Regulation of β_2_AR Translation and Function in Failing Hearts

**DOI:** 10.1161/CIRCRESAHA.123.323174

**Published:** 2023-10-23

**Authors:** Zoe Kwan, Binoy Paulose Nadappuram, Manton M. Leung, Sanika Mohagaonkar, Ao Li, Kumuthu S. Amaradasa, Ji Chen, Stephen Rothery, Iyobel Kibreab, Jiarong Fu, Jose L. Sanchez-Alonso, Catherine A. Mansfield, Hariharan Subramanian, Alexander Kondrashov, Peter T. Wright, Pamela Swiatlowska, Viacheslav O. Nikolaev, Beata Wojciak-Stothard, Aleksandar P. Ivanov, Joshua B. Edel, Julia Gorelik

**Affiliations:** National Heart and Lung Institute (Z.K., S.M., A.L., K.S.A., J.C., I.K., J.F., J.L.S.-A., C.A.M., P.S., B.W.-S., P.T.W., J.G.), Imperial College London, United Kingdom.; Department of Chemistry (Z.K., B.P.N., A.P.I., J.B.E.), Imperial College London, United Kingdom.; Department of Pure and Applied Chemistry, University of Strathclyde, United Kingdom (B.P.N.).; Sir William Dunn School of Pathology, University of Oxford, United Kingdom (M.M.L.).; Division of Cancer and Stem Cells, University of Nottingham Biodiscovery Institute, United Kingdom (A.K.).; Gene Regulation and RNA Biology, School of Pharmacy, University of Nottingham, United Kingdom (A.K.).; FILM Facility, Imperial College London, United Kingdom (S.R.).; Institute of Experimental Cardiovascular Research, University Medical Center, Hamburg-Eppendorf, Germany (H.S., V.O.N.).; School of Life and Health Sciences, University of Roehampton, United Kingdom (P.T.W.).

**Keywords:** animal, cardiomyocytes, heart failure, infarction, microtubules

## Abstract

**Background:**

β_1_AR (beta-1 adrenergic receptor) and β_2_AR (beta-2 adrenergic receptor)-mediated cyclic adenosine monophosphate signaling has distinct effects on cardiac function and heart failure progression. However, the mechanism regulating spatial localization and functional compartmentation of cardiac β-ARs remains elusive. Emerging evidence suggests that microtubule-dependent trafficking of mRNP (messenger ribonucleoprotein) and localized protein translation modulates protein compartmentation in cardiomyocytes. We hypothesized that β-AR compartmentation in cardiomyocytes is accomplished by selective trafficking of its mRNAs and localized translation.

**Methods:**

The localization pattern of β-AR mRNA was investigated using single molecule fluorescence in situ hybridization and subcellular nanobiopsy in rat cardiomyocytes. The role of microtubule on β-AR mRNA localization was studied using vinblastine, and its effect on receptor localization and function was evaluated with immunofluorescent and high-throughput Förster resonance energy transfer microscopy. An mRNA protein co-detection assay identified plausible β-AR translation sites in cardiomyocytes. The mechanism by which β-AR mRNA is redistributed post–heart failure was elucidated by single molecule fluorescence in situ hybridization, nanobiopsy, and high-throughput Förster resonance energy transfer microscopy on 16 weeks post–myocardial infarction and detubulated cardiomyocytes.

**Results:**

β_1_AR and β_2_AR mRNAs show differential localization in cardiomyocytes, with β_1_AR found in the perinuclear region and β_2_AR showing diffuse distribution throughout the cell. Disruption of microtubules induces a shift of β_2_AR transcripts toward the perinuclear region. The close proximity between β_2_AR transcripts and translated proteins suggests that the translation process occurs in specialized, precisely defined cellular compartments. Redistribution of β_2_AR transcripts is microtubule-dependent, as microtubule depolymerization markedly reduces the number of functional receptors on the membrane. In failing hearts, both β_1_AR and β_2_AR mRNAs are redistributed toward the cell periphery, similar to what is seen in cardiomyocytes undergoing drug-induced detubulation. This suggests that t-tubule remodeling contributes to β-AR mRNA redistribution and impaired β_2_AR function in failing hearts.

**Conclusions:**

Asymmetrical microtubule-dependent trafficking dictates differential β_1_AR and β_2_AR localization in healthy cardiomyocyte microtubules, underlying the distinctive compartmentation of the 2 β-ARs on the plasma membrane. The localization pattern is altered post–myocardial infarction, resulting from transverse tubule remodeling, leading to distorted β_2_AR-mediated cyclic adenosine monophosphate signaling.

**Novelty and Significance**

**What Is Known?**

- β_1_AR (beta-1 adrenergic receptor) and β_2_AR (beta-2 adrenergic receptor) are functionally compartmentalized in cardiomyocytes.
- The mRNA for β_2_AR contain specific structural elements for translational (inhibition) and mRNA localization control, whereas β_1_AR mRNA does not. Some but not all mRNAs in cardiomyocytes are trafficked using the microtubule network.
- Heart failure alters cardiac function in several ways, including redistribution and dysregulation of βAR signaling.

**What New Information Does This Article Contribute?**

- β_2_AR but not β_1_AR mRNA is trafficked in cardiomyocytes using the microtubule network.
- β_2_AR protein localization and function at the plasma membrane compartment is regulated using the microtubule network.
- In failing cardiomyocytes, β_2_AR mRNA is distributed differently than in normal myocytes.
- In heart failure, the dismantling of the transverse tubules (t-tubules) (detubulation) is a driving force leading to β_2_AR redistribution, on both mRNA, protein and functional level.

Both β_1_AR and β_2_AR could be found on the plasma membrane of cardiomyocytes, with β_2_AR predominantly compartmentalized to the t-tubules in healthy cardiomyocytes and β_1_AR found throughout the sarcolemma membrane. In failing hearts, β_2_AR redistributes from the t-tubules toward the sarcolemma membrane contributing to the loss in precise regulation of β_2_AR-mediated cAMP signaling.

Using smFISH and nanoscale tweezer subcellular biopsy, we have discovered β_1_AR and β_2_AR mRNA distribute differently in cardiomyocytes, and that both mRNAs redistributed in failing hearts. Using a microtubule depolymerizing agent, we demonstrated on mRNA, protein, and functional levels that β_2_AR but not β_1_AR distribution is regulated by the microtubule network. When t-tubules were dismantled by a drug-induced acute detubulation, the loss of t-tubules lead to a physical redistribution of β_2_AR.

Taken together, we have demonstrated the importance of βARs localization in regulating proper cardiac function. By aiming to restore a proper structural landscape (t-tubules, microtubules), which will lead to a restoration of correct distribution of β2AR on both mRNA and protein level, one could potentially restore the β_2_AR-mediated cardiac function in failing hearts.

Heart failure (HF) is one of the most common cardiac-related causes of death globally.^1,2^ Cardiomyocytes, which comprise the bulk of the human heart, undergo various dynamic physiological changes during HF, such as distortion of the β-ARs (β-adrenergic receptors) signaling pathways. There are 3 types of β-ARs in the human myocardium, with β_1_AR (beta-1 adrenergic receptor) and β_2_AR (beta-2 adrenergic receptor) being the 2 most abundant subtypes, existing at roughly 70:30 ratio.^3–6^ Although β_1_ARs and β_2_ARs are highly similar in structure, they also have some distinctive features. For example, both β_1_AR and β_2_AR could trigger stimulatory Gαs-protein activation, but the latter also can couple with inhibitory Gαi-protein making it possible for β_2_AR to play multiple regulatory roles in mammalian hearts.^7–11^ In failing hearts, β-AR signaling undergoes remodeling, a highly complex process, with features such as equalized expression and function between β_1_AR and β_2_AR (uncoupling from G proteins).^12–14^

Emerging evidence suggests that different β-AR subtypes modulate the downstream cyclic adenosine monophosphate (cAMP) signaling pathway differently, resulting in the differential compartmentation and signaling profile of the 2 β-AR subtypes.^15^ A 2010 study previously demonstrated that, unlike β_1_AR, distributed throughout the plasma membrane, β_2_AR is compartmentalized mainly to the transverse tubules (t-tubules) in healthy cardiomyocytes. However, this difference is lost in HF, where β_2_ARs are physically redistributed from the t-tubules to the plasma membrane.^16^ The mechanism of this redistribution remains unclear. What is known, however, is that t-tubule remodeling in cardiomyocytes is one of the hallmarks of HF and an indicator of the disease’s progression.^17–22^ In mammals, the β_1_AR and β_2_AR receptor proteins are encoded by the Adrb1 and Adrb2 genes, respectively, both genes having no introns. Several studies highlighted the importance of regulation at the level of mRNA localization and protein synthesis for β_2_AR. It has been demonstrated that Adrb2 mRNA, unlike Adrb1, can be initially translationally silent.^18^ A 1994 study has shown that the 5′ upstream open reading frame in the Adrb2 mRNA translates into a small peptide product that could inhibit the receptor’s synthesis.^23^ A more recent study has demonstrated that in DT1-MF2 and A431 cells, the binding of nucleocytoplasmic shuttling RNA-binding protein HuR to the 3′-UTR of Adrb2 mRNA is crucial in its translational silencing and trafficking to the cell membrane.^24^ The authors showed that downregulation of HuR led to premature translational initiation of Adrb2 mRNA and mislocalization of the protein from the cell periphery to other subcellular locations,^24^ highlighting the importance of mRNA trafficking for appropriate β_2_AR receptor localization.

While one common mechanism of protein and signal compartmentation in some mammalian cells, such as neurons, is by localized protein translation,^25,26^ it was previously unclear whether this applies to mammalian cardiomyocytes. However, in recent years, emerging evidence has demonstrated that at least some mRNAs in cardiomyocytes are locally translated into proteins in individual sarcomeres. In 2018, Lewis et al demonstrated for the first time that some sarcomeric proteins, including Myh6 (myosin heavy chain 6), are locally translated in individual sarcomeres. In addition, the authors found that many sarcomeric proteins are also locally degraded by ubiquitin.^27^ Following that study, Scarborough et al^28^ revealed that these sarcomeric factors were not the only transcripts found to be translated throughout mammalian cardiomyocytes, but in fact, it is a general phenomenon involving many transcripts in these cells. The mechanism for how mRNAs in mature mammalian cardiomyocytes are transported has also been suggested by the same study in which the microtubule network plays a key role.

Microtubules, a major component of the cell cytoskeleton, play a crucial role in intracellular trafficking in mammalian cells. Biological molecules such as mRNAs and proteins can be attached to microtubules as cargo complexes via motor proteins (eg, dynein), where multiple cargos can bind to multiple motor proteins of the same or opposite polarity.^29–32^ In the 2021 study conducted by Scarborough et al,^28^ the authors found that in adult rat ventricular myocytes (ARVMs), most mRNAs are trafficked using the microtubule network. The authors demonstrated that when the microtubule network was disrupted, mRNAs, rRNAs (translational machinery), and nascent protein products relocalized to the perinuclear region of ARVM, suggesting that an intact microtubule network is crucial to proper mRNA transportation in these cells for localized translation to take place, hence enabling proper localization of the translated proteins. A more recent study utilizing a highly sensitive and specific fluorescent in situ hybridization (FISH) technique that can visualize 18s associated mRNAs of interest also demonstrated that in murine cardiomyocytes, some but not all mRNAs are locally translated into proteins.^33^ It suggested that protein translation in cardiomyocytes can be conducted in different subcellular compartments and may be subject to remodeling upon local stimulation. Other than mRNP (messenger ribonucleoprotein) trafficking, the microtubule network has also been reported to be involved in regulating t-tubule stability in cardiomyocytes via anchoring proteins such as Junctophilin-2 and BIN1,^34–37^ further indicating the importance of microtubule homeostasis in regulating normal cardiac function.

In this study, we aimed to investigate whether the compartmentation of β_2_AR in cardiomyocytes is accomplished by selective trafficking of its mRNAs. We were also interested in finding out whether the localization pattern of β_2_AR mRNA in failing cardiomyocytes changes and if this change is associated with the loss of compartmentation of the receptor protein on the plasma membrane. Since β_2_AR mRNA has a unique feature where it is initially translationally repressed after nuclear export,^24^ we hypothesized that localization of β_2_ARs, but not β_1_ARs, depends on local translation and hence proper mRNA trafficking in cardiomyocytes.

## METHODS

### Data Availability

Detailed methodology and protocols are included in the supplementary section.

## RESULTS

### A New Software for Clustering Analysis of smFISH Images on Multinucleated Cells

A clustering analysis approach for analyzing FISH images was reported by Hye et al utilizing the concept of polarization index (PI) and dispersion index (DI). Later, Stueland et al developed the original algorithm into software to automate the image analysis process.^38^ The original algorithm was designed for analyzing single-nucleated cells, assuming the cell centroid and the nucleus centroid to be the same—which is the case for most single-nucleated cells. This is, however, not the case in ARVM, as these are binucleated cells. A new algorithm suitable for analyzing cells with multiple nuclei was therefore developed.

The geometric center of a cell, the nucleus, and the population of FISH signals in a particular digital image were each defined as a centroid (the averaged middle point of a particular cluster of fluorescent signals):


$$\begin{array}{l} Centroid=\left(\frac{1}{n}\overset{n}{\underset{i}{\sum }}x_{i},\frac{1}{n}\overset{n}{\underset{i}{\sum }}y_{i}\right)=\left(\bar{x},\bar{y}\right) \end{array}$$

Where x and y are the pixel coordinates of the boundary of the cell, the nucleus, or the recognized FISH signals, and n is the number of points that define the cell boundary, the nucleus, and the FISH signals. To estimate the distribution of a given RNA cluster in relation to the cell size/shape across different cells, the radius of gyration (scalar) of the cell was first calculated using the cell mask.


$$\begin{array}{l} Radiusofgyration\left(Rg\right)=\sqrt{\frac{\overset{n}{\underset{i}{\sum }}\left[\begin{array}{c} {\left(x_{i}-{\bar{x}}_{Cell}\right)}^{2}+{\left(y_{i}-{\bar{y}}_{Cell}\right)}^{2} \end{array}\right]}{n}} \end{array}$$

Where ${\bar{x}}_{Cell}$, ${\bar{y}}_{Cell}$ is the coordinates of the cell centroid, (x_i_, y_i_) is the collection of coordinates defining the cell boundary.

**Figure 1.**
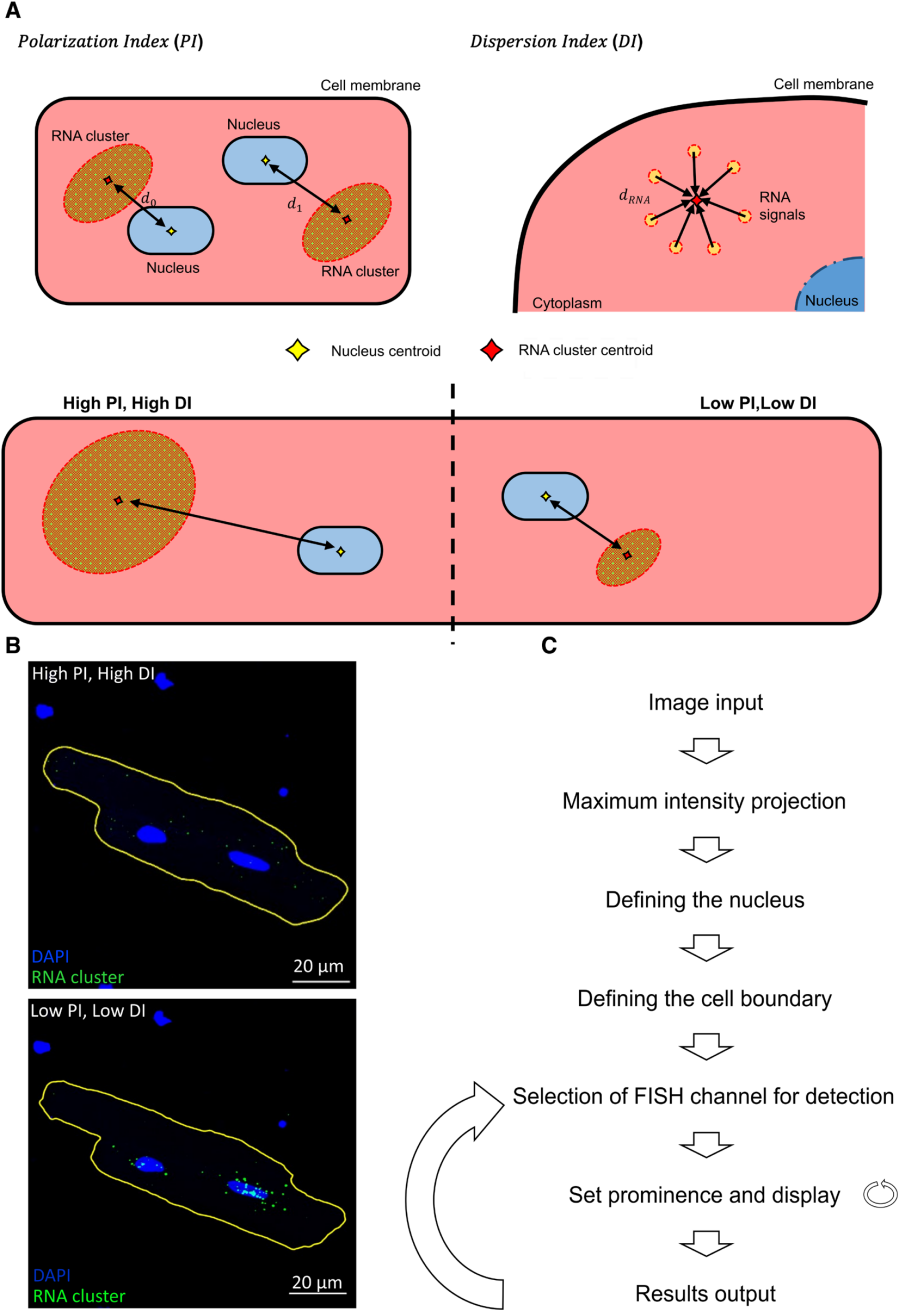
**Description of the algorithm used for understanding RNA distribution patterns in multinucleated cells by clustering analysis. A**, Schematic of the concept of polarization index (PI) and dispersion index (DI). A high polarization index indicates a given RNA cluster traveled further away from the nucleus; a high dispersion index suggests a given RNA cluster is more dispersed throughout the cell. **B**, Polarization index and dispersion index demonstrated on a smFISH image. An RNA cluster with high PI and DI suggests that the RNA are found dispersed throughout the cell far away from the nucleus. An RNA cluster with low PI and DI suggests that the labeled RNA molecules are found to be highly concentrated at the perinuclear region. **C**, The workflow of Fiji macro-operation and data output.


The displacement (d_0_) between the FISH signal cluster centroid and the nucleus centroid is described as the PI (Figure 1A). The longer the distance between the RNA signal cluster and the nucleus is, the greater the PI is. To compare the displacement across cells, we normalize the size of the displacement vector with the radius of the gyration of the cell. The cellular radius of gyration (Rg_Cell_) describes the cell morphology as a linear scalar to match the size of the displacement scalar.

$$\begin{array}{l} PolarisationIndex\left(PI\right)=\sqrt{\frac{\begin{array}{c} {\left({\bar{x}}_{RNA}-{\bar{x}}_{Nucleus}\right)}^{2}+\;{\left({\bar{y}}_{RNA}-{\bar{y}}_{Nucleus}\right)}^{2} \end{array}}{Rg_{cell}}} \end{array}$$

The DI was used to describe how tightly packed a given RNA cluster is. It summarizes the displacements between all RNA fluorescent signals and the RNA cluster centroid. The more dispersed the RNA signal, the higher the DI will be. As an intracellular component, RNA distribution is strongly influenced by the shape of the cell. The second moment of area of the RNA cluster and the cell was used to describe how fluorescent signals/points are distributed. The DI was expressed as the second moment of area of a given RNA cluster (the distribution of the RNA cluster) normalized to the second moment of area of the cell (the shape of the cell).


$$\begin{array}{l} Secondmoment_{RNA}=\mu_{2}=\frac{1}{n}\overset{n}{\underset{i}{\sum }}\left[\begin{array}{c} {\left(x_{i}-{\bar{x}}_{RNA}\right)}^{2}+\;{\left(y_{i}-{\bar{y}}_{RNA}\right)}^{2} \end{array}\right] \end{array}$$

$$\begin{array}{l} Secondmoment_{Cell}={\mu_{2}}^{\prime }=\frac{1}{m}\overset{m}{\underset{i}{\sum }}\left[\begin{array}{c} {\left(x_{i}-{\bar{x}}_{Cell}\right)}^{2}+\;{\left(y_{i}-{\bar{y}}_{Cell}\right)}^{2} \end{array}\right] \end{array}$$

$$\begin{array}{l} DispersionIndex\left(DI\right)=\frac{\mu_{2}}{{\mu_{2}}^{\prime }} \end{array}$$

Where ${\bar{x}}_{Cell}$, ${\bar{y}}_{Cell}$ is the coordinates of the cell centroid, (x_i_, y_i_) is the collection of coordinates defining the cell boundary.

In the case of multinucleated cells, the algorithm assumes that an individual RNA signal originated from its immediate nucleus. Therefore, within the same cell, each RNA molecule signal is assigned to one of the 2 nuclei, and each nucleus will result in 2 indexes: 1 PI and 1 DI. The averaged PI/DI between all nuclei in that particular cell is used to represent the overall PI/DI for that cell.

### Adrb1 and Adrb2 Are Differentially Localized in ARVM

**Figure 2.**
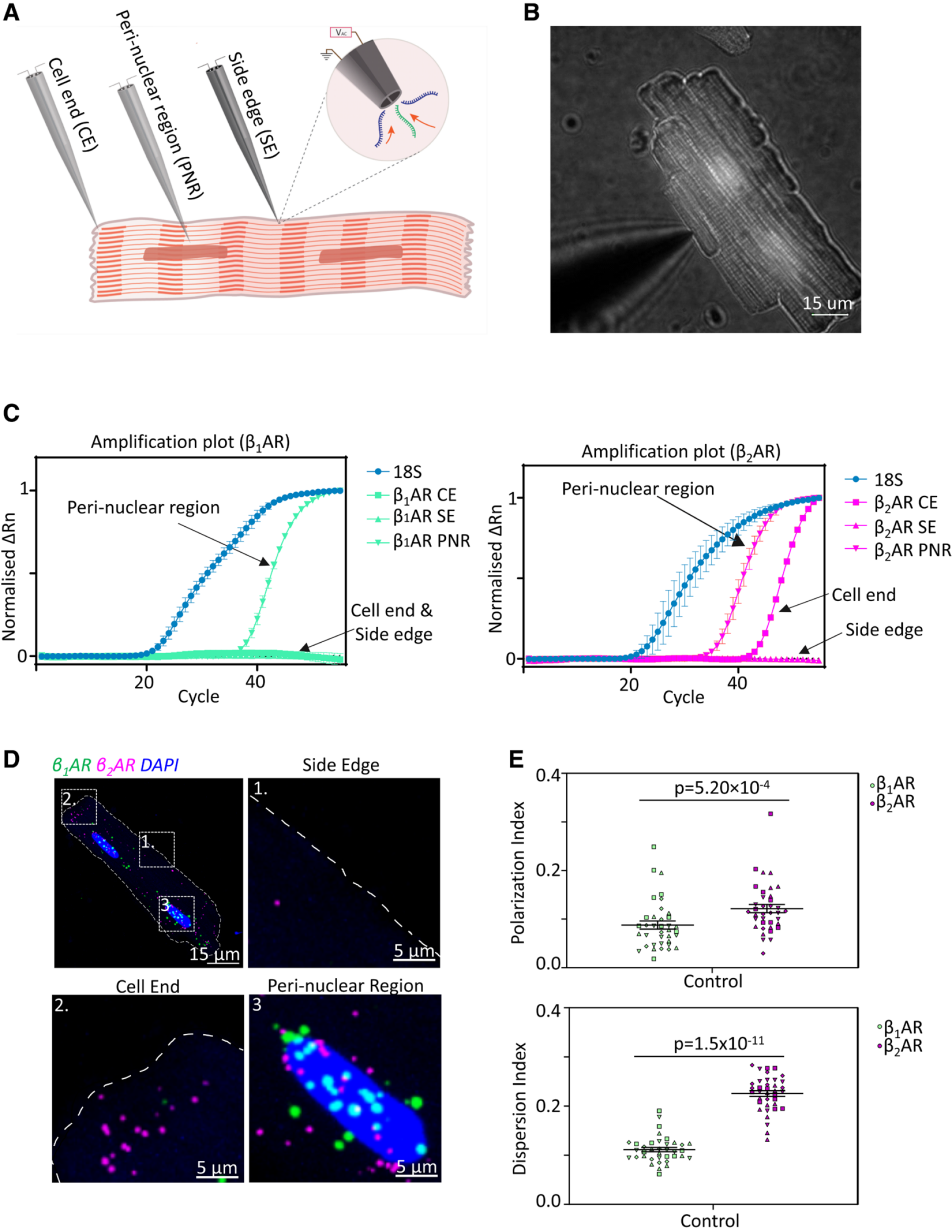
**β_1_AR (beta-1 adrenergic receptor) and β_2_AR (beta-2 adrenergic receptor) mRNAs are differentially distributed in healthy cardiomyocytes. A**, A schematic of nanobiopsy procedure to obtain subcellular content using nanoscale tweezers. **B**, Microscopic image showing a control rat cardiomyocyte undergoing nanoscale biopsy. **C**, Representative reverse transcription-quantitative PCR (RT-qPCR) amplification plots of nanobiopsy extracted from different cellular compartments in healthy cardiomyocytes. β_1_AR mRNA is mostly detected at the perinuclear region (PNR) but rarely at the cell ends (CE) and side edges (SE). β_2_AR mRNAs are found near the nucleus and often closer to the plasma membrane compartments. 18S was used as housekeeping control and is detected in all nanobiopsy analyzed. N=5, n≥15. **D**, RNAscope smFISH images showing β_1_AR (green) and β_2_AR (magenta) transcripts distribution in healthy cardiomyocytes. β_1_AR mRNAs were found clustering around the perinuclear region, whereas β_2_AR mRNAs are distributed throughout the cell (**E**) resulting in a higher polarization and dispersion index when compared with β_1_-AR. N=4, n=37. Data are represented as mean±SEM. Statistical significance was determined using the Wilcoxon matched-pairs signed rank test.

To study the localization pattern of β_1_AR or β_2_AR mRNAs in ARVM, a dielectrophoretic (DEP) nanotweezer single-cell sampling technique was used.^39^ These nanoscale tweezers generate an electric field gradient upon applying an alternating current across the 2 nanoelectrodes at their tip. This allows highly localized trapping and extraction of mRNA molecules from individual ARVMs. Highly localized single-cell biopsies (within 300 nm from the tweezer tip) were reproducibly obtained from the cell end, perinuclear region, and side edge of live adult rat cardiomyocytes in a highly precise manner (Figure 2A and 2B). TaqMan reverse transcription-quantitative PCR (RT-qPCR) analysis was used to detect and quantify the presence of β_1_AR or β_2_AR transcript in different cellular regions. The results revealed that most β-AR transcripts were found in the perinuclear regions regarding the subtype. However, some β_2_ARs were also detected near the cell periphery (Figure 2C).

To confirm this finding, a highly sensitive and specific amplification-based Single molecule fluorescence in situ hybridization (smFISH) technology RNAscope was utilized. Qualitatively, it was revealed that β_1_AR transcripts were found mainly in the perinuclear region, whereas β_2_AR mRNAs were distributed throughout the cells (Figure 2D). Clustering analysis of the smFISH images showed β_1_AR mRNA clustered around the nucleus with PIs and DIs of 0.088 and 0.111, respectively, whereas β_2_AR mRNA distribution is more polarized and dispersed with PIs and DIs of 0.122 and 0.226, respectively (Figure 2E).

### An Intact Microtubule Network Is Essential in β_2_AR mRNA but Not β_1_AR mRNA Trafficking

To test whether the differential localization of the 2 β-AR mRNAs is a result of spatially regulated mRNA trafficking via the microtubule network, we treated cardiomyocytes with 2 μM vinblastine to disrupt the microtubule network in ARVM for 18 hours and observed its effect on β_1_AR and β_2_AR mRNAs localization. We have confirmed by immunostaining that treating ARVM with 2 μM vinblastine for 18 hours sufficiently disrupted the microtubule network and that such treatment did not cause significant β_1_AR and β_2_AR gene expression alteration as confirmed by our RT-qPCR analysis on total extracted RNA (Figure S1).

**Figure 3.**
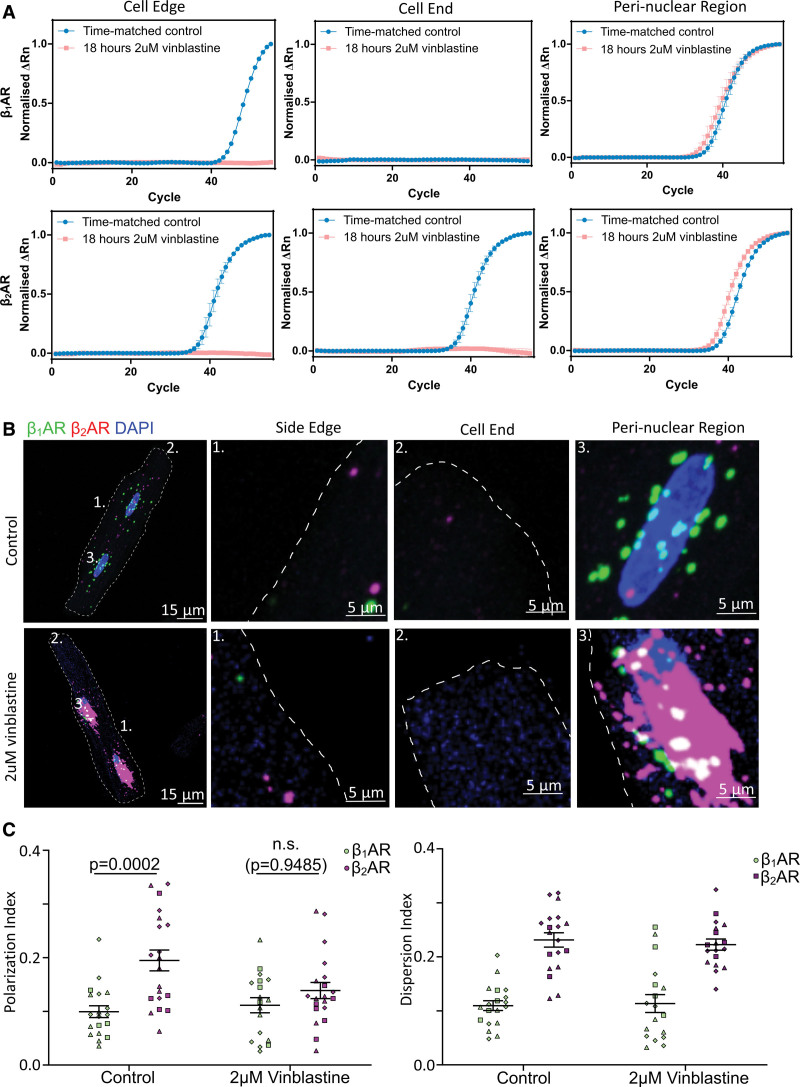
**β_2_AR (beta-2 adrenergic receptor) mRNA transport in cardiomyocytes is microtubule dependent. A**, When 2-μM vinblastine was used to disrupt microtubules from cardiomyocytes, β_2_AR became less likely to be detected close to the membrane but became more abundant around the nucleus, compared with a time-matched (18-hour incubation in culture) control. N=3, n≥16. **B**, Representative RNAscope single molecule fluorescence in situ hybridization (smFISH) images showing that in 2-μM vinblastine-treated cells and time-matched control, β_2_AR (magenta) mRNAs are redistributed to the perinuclear region from the distal cytosol of the cell. β1AR (beta-1 adrenergic receptor; green) mRNA, on the other hand, did not seem to be largely affected. **C**, Without an intact microtubule in cardiomyocytes, the differential localization of β_1_AR and β_2_AR transcripts was lost, and the difference in polarization between the 2 mRNAs of interest are no longer statistically significant. Data were represented as mean±SEM. Statistical significance was determined using 2-way ANOVA with post hoc Tukey multiple comparisons test. N=3, n≥18.

After testing the efficacy of vinblastine in depolymerizing the microtubule network, we proceeded to extract nanobiopsy samples from the different cellular regions of interest from control and vinblastine-treated ARVM. RT-qPCR analysis of the nanobiopsy samples showed that β_2_AR mRNAs relocalized from the cell periphery (side edges and cell ends) to the perinuclear region of ARVM after microtubule disruption (Figure 3A). Once again, the RT-qPCR results were validated using smFISH, which agreed with our qPCR findings. smFISH revealed that β_1_AR-mRNA localization in vinblastine-treated cells was not statistically significant from control cells. However, β_2_AR mRNAs were redistributed from the cell periphery to the perinuclear region, and differences in polarization between the 2 transcripts were lost, suggesting β_2_AR-mRNA transportation was dependent on the microtubule network (Figure 3B and 3C).

Since 18 hours of 2 μM vinblastine treatment has been found to have no statistically significant effect on β_1_AR and β_2_AR expression levels, we concluded that the changes we observed in β_2_AR localization following microtubule disruption were a result of changes in physical redistribution of the mRNA molecules, but not due to altered gene expression. To control for drug-specific effect of vinblastine, the same smFISH experiment was performed on 10 μM nocodazole-treated cells. In all these cases, a similar trend was observed where β_2_AR mRNAs, but not β_1_AR, trafficking in cardiomyocytes was affected by microtubule disruption (Figure S3).

### An Intact Microtubule Is Crucial for β_2_AR Trafficking, Translation, and Function

**Figure 4.**
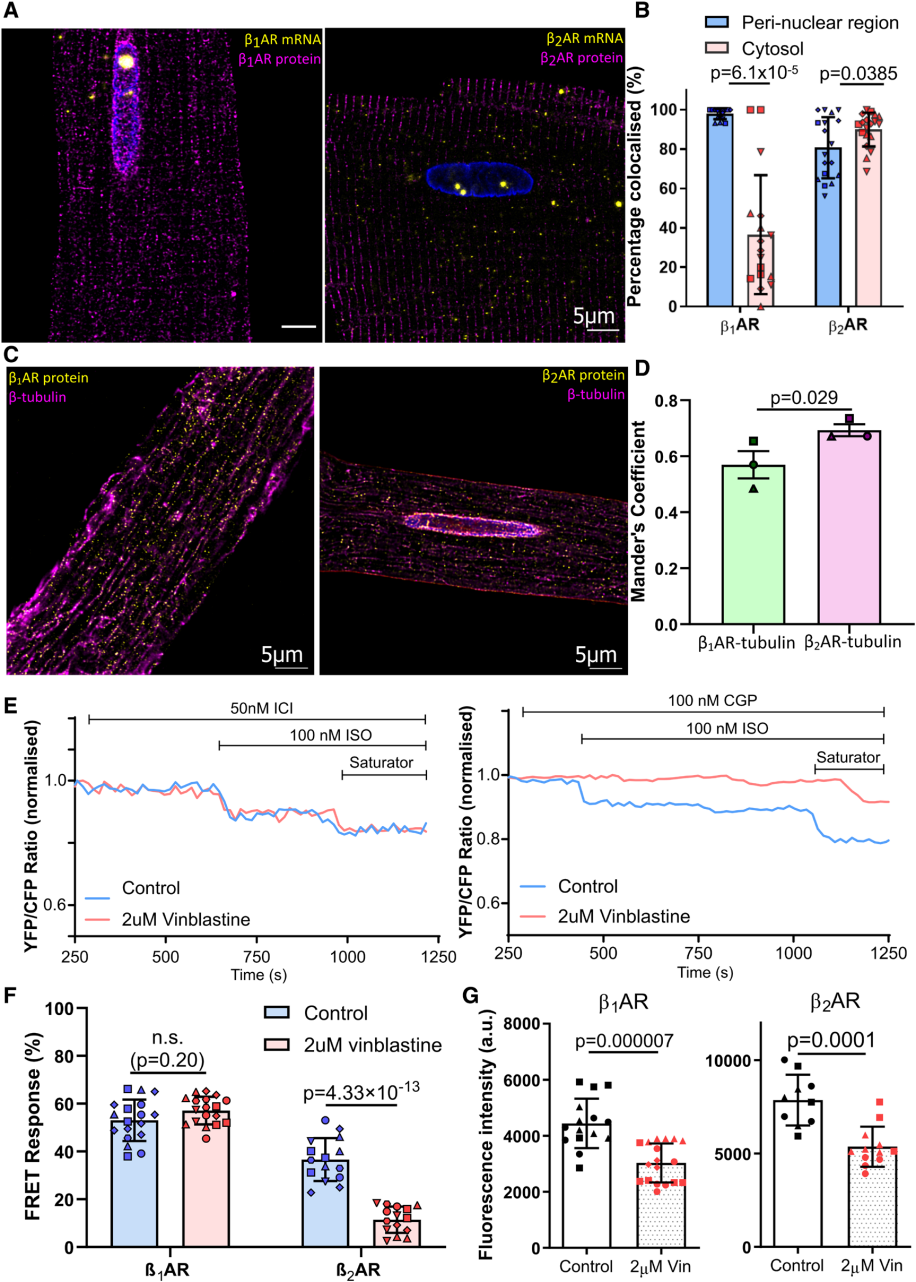
**An intact microtubule is crucial for β_2_AR (beta-2 adrenergic receptor) trafficking, translation, and function. A**, Representative fluorescent labeling of β_1_AR (beta-1 adrenergic receptor) and β_2_AR mRNA and their corresponding proteins. **B**, Quantification of mRNA protein co-detection shows that >90% of β_1_AR mRNA colocalized with its protein at the perinuclear region, compared with <40% in the cytosol. β_2_AR mRNA and protein tend to colocalize in the cytosol, further away from the nucleus. Colocalization was defined as the distance of a given RNA signal is <0.1 µm from the closest corresponding protein signal. A detailed protocol can be found in the supplementary methodology. Magenta: proteins; yellow: RNA molecules. N≥3, n≥17. Statistical significance was determined using Wilcoxon matched-pairs signed rank tests. **C**, Representative fluorescent labeling of either β_1_AR or β_2_AR protein and tubulin. **D**, β_2_AR proteins colocalize with the microtubule network at a higher degree than their β_1_AR counterpart, suggesting an intact microtubule is essential for β_2_AR protein trafficking. N=3, n=12 to 21. Statistical significance was determined using a nested *t* test. **E**, Representative Förster resonance energy transfer (FRET) traces of β_1_AR and β_2_AR specific cAMP response in control and 2-μM vinblastine-treated cardiomyocytes, normalized to baseline. β-AR subtype-specific blocker (β_1_-blocker 100 nmol/L CGP 20712 **A**, β_2_-blocker 50 nmol/L ICI-118 551) was added, and the nonselective β-agonist 100 nmol/L isoproterenol (ISO) was used to trigger β-AR subtype-specific response. A saturator (100 µmol/L IBMX+10 µmol/L forskolin) was then added to trigger maximum cAMP response of the cell. **F**, β_1_AR- and β_2_AR-specific cAMP response of control and 2 μM vinblastine-treated cardiomyocytes, normalized to saturator response. β_2_AR-specific cAMP response is significantly lower in microtubule-disrupted cardiomyocytes, whereas β_1_AR-specific cAMP response was unaffected despite the absence of an intact microtubule network. Data were represented as mean±SEM. N=6, n≥15. Statistical significance was determined by 2-way ANOVA followed by post hoc Tukey multiple comparisons test. **G**, Quantification of immunofluorescent images of β_1_AR and β_2_AR in control and vinblastine-treated cardiomyocytes (representative fluorescent images are available in Figure S3). The amount of both β_1_AR and β_2_AR proteins on the cell surface was significantly reduced as the microtubule network was disrupted in cardiomyocytes. Statistical significance was determined by the Mann-Whitney *U* test (β_1_AR) or the unpaired *t* test (β_2_AR).

To investigate whether localization of the β-AR mRNAs to the cell periphery resulted in localized protein translation, we performed a single-cell RNA-protein co-detection assay to visualize the localization of β-AR mRNAs and their corresponding translated proteins in the same single isolated ARVM. The distance analysis has revealed that β_1_AR mRNA molecules and their corresponding translated proteins almost always colocalize in the perinuclear region, but only in <40% in other parts of the cell. On the other hand, β_2_AR mRNA molecules and their translated proteins colocalized at a much higher degree at >80% on average, regardless of the sub-compartments; in fact, β_2_AR mRNAs tended to colocalize with its translated proteins more in the cytosol (89.9%) than in the perinuclear region (80.1%). This result suggests that β_2_AR mRNA is much more likely to be locally translated into proteins compared with its β_1_AR counterpart (Figure 4A and 4B).

We next studied if the newly synthesized β-AR proteins are located close to microtubules by immunostaining of β-tubulin and β-AR proteins. We analyzed the images using Mander coefficient, and it was found that β_2_AR proteins indeed tended to colocalize with the microtubule network at a significantly higher degree as compared with β_1_AR proteins, suggesting that β_2_AR distribution relies on the microtubule network to a much higher degree than its β_1_AR counterpart (Figure 4C and 4D).

To elucidate whether β_1_AR and β_2_AR mRNA localization and functional properties of their protein products depend on microtubules, we performed a Förster resonance energy transfer (FRET)-microscopy experiment by transducing cardiomyocytes with a plasma membrane-localized cAMP biosensor pmEpac2. We then stimulated β_1_AR or β_2_AR subtype-specific cAMP responses on the plasma membrane compartment in control and 2 μM vinblastine-treated ARVMs. As expected, selective activation of β_1_AR resulted in a substantial increase in cAMP production in both control and vinblastine-treated ARVM (Figure 4E). Differences in the FRET response between the control and vinblastine-treated group (Figure 4F) were not statistically significant, indicating that activation of cAMP synthesis by β_1_AR receptors on the plasma membrane is independent of microtubules. However, β_2_AR-specific cAMP response in vinblastine-treated cardiomyocytes was significantly lower than in control cells (Figure 4E and 4F), suggesting that microtubule disruption significantly impairs β_2_AR receptor function on the plasma membrane. We therefore concluded that a microtubule-dependent mRNA trafficking mechanism governs the compartmentation/function of β_2_AR but not β_1_AR, on the plasma membrane. To control for the drug-specific effect of vinblastine, we also repeated the same FRET experiment on 10 μM nocodazole-treated ARVMs, and we observed the same trend where β_2_AR, but not β_1_AR, receptor function was affected by microtubule disruption in cardiomyocytes (Figure S3).

To investigate if microtubule disruption would reduce the levels of β-AR, we quantified the amount of membrane-localized β_1_AR and β_2_AR proteins in control and vinblastine-treated cardiomyocytes after immunostaining. Interestingly, we found that the levels of both β_1_AR and β_2_AR localized to the plasma membrane were reduced at the edge of the cell following microtubule disruption (Figure 4F; Figure S4A). The distribution of both proteins was altered following vinblastine treatment, as both the density and regular distribution pattern of both β-AR subtype proteins were reduced (Figure S4A). To validate this result, a radioligand binding assay was performed to determine the ratio between β_1_AR and β_2_AR in control and vinblastine-treated cardiomyocytes. Analysis of the whole microsomal fraction containing all membrane compartments did not show any difference between treated and untreated cells in terms of β_2_AR amounts (Figure S4A). However, strikingly, microtubule disruption led to a statistically significant reduction of the relative ratio of β_2_AR/β_1_ARs but almost no β_2_AR binding detectable in the plasma membrane fraction, suggesting that the abovementioned changes in receptor localization may result from altered trafficking and distinct subcellular distribution in various membrane compartments (Figure S4B). On the contrary, actin depolymerization had no statistically significant effect on β_1_AR or β_2_AR protein localization or their function in the plasma membrane (Figure S7).

### Both β_1_AR and β_2_AR Transcripts Are Redistributed in Failing Cardiomyocytes

After establishing differences in β_1_AR and β_2_AR mRNA localization, translation, and receptor function in healthy ARVMs, we next examined if their localization has changed in HF and whether redistribution of β_2_AR proteins in failing cardiomyocytes, reported in one of our previous studies,^16^ can be related to mislocalization of their mRNA.

**Figure 5.**
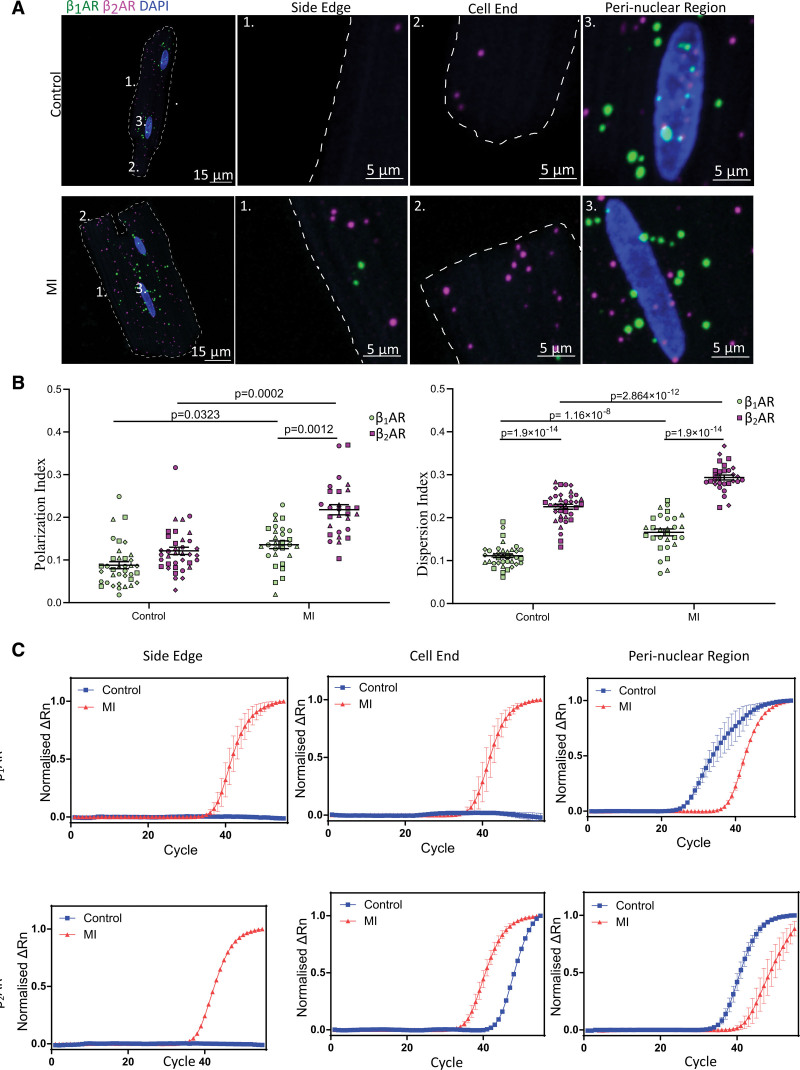
**Both β_1_AR (beta-1 adrenergic receptor) and β_2_AR (beta-2 adrenergic receptor) transcripts are redistributed in failing cardiomyocytes. A**, Post–heart failure, both β_1_AR and β_2_AR mRNAs were found to have redistributed where they moved closer to the plasma membrane. **B**, The mRNA redistribution was reflected in the clustering analysis as an increase in both polarization index (PI) and dispersion index (DI) for both mRNAs of interest. Although both transcripts have redistributed post–myocardial infarction (MI), the differences between β_1_AR and β_2_AR persist. N≥3, n=29. Data are represented as mean±SEM. Statistical significances were determined using 2-way ANOVA with Sidak multiple comparisons test. **C**, Representative reverse transcription-quantitative PCR (RT-qPCR) amplification curves of nanobiopsy samples extracted from different cellular compartments confirm the changes in both β_1_AR and β_2_AR mRNA localization post-MI, where it became more likely to detect both β_1_AR and β_2_AR mRNA close to the cell membrane in failing cardiomyocytes. N=5, n≥15.

We first performed smFISH on healthy and failing ARVMs. It was found that there was indeed redistribution of the 2 transcripts in failing cells compared with control (Figure 5A and 5B). Clustering analysis of the images showed that both β_1_AR and β_2_AR-mRNAs became more polarized and dispersed in failing ARVMs, indicating that both transcripts migrated further away from the nucleus and became less clustered.

Since hypertrophy is a common feature of failing cardiomyocytes, the clustering analysis results must be confirmed with a different technique, as the PI and DI were calculated based on the cell morphology. To address this, we performed a correlation analysis and found no statistically significant correlation between the cell area and the PI (Figure S5). Next, to confirm our smFISH analysis data, we analyzed nanoscale biopsies from the 3 cellular compartments of interest (perinuclear, cell end, and side edge) using TaqMan RT-qPCR. The results confirmed a redistribution of β_1_AR and β_2_AR mRNAs from the perinuclear region to cytosol close to the cell periphery in failing ARVM, consistent with the smFISH results (Figure 5C).

### T-Tubule Remodeling Contributes to β_2_AR Redistribution in HF

To elucidate the potential mechanism leading to β-AR mRNA redistribution in failing cardiomyocytes, we used a recently discovered detubulation agent, imipramine, to remove t-tubules from control cardiomyocytes, to investigate whether t-tubule remodeling, a known feature in failing cardiomyocytes, contributes to β-AR mRNA redistribution post–myocardial infarction.

**Figure 6.**
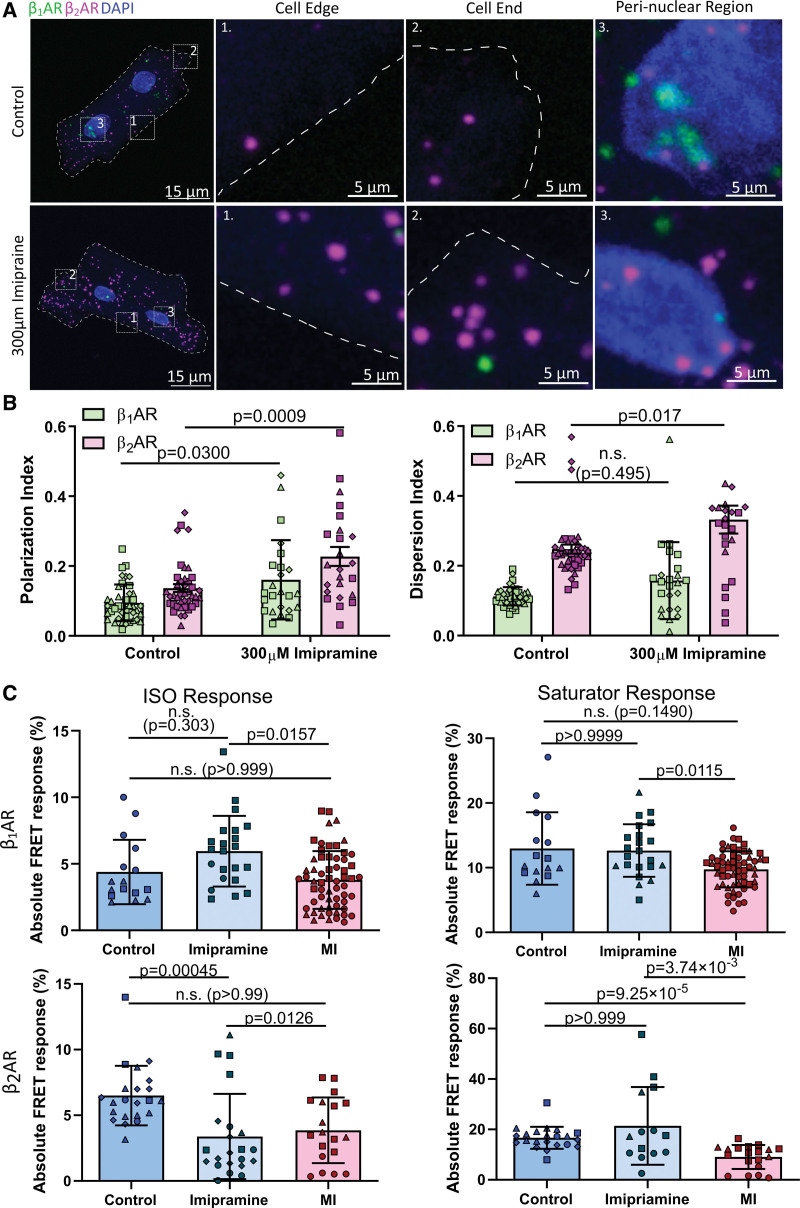
**Detubulation contributes to β_2_AR (beta-2 adrenergic receptor) redistribution in heart failure. A**, RNAscope smFISH images showing β_1_AR (beta-1 adrenergic receptor) and β_2_AR mRNA distribution in control and 300-μM imipramine-treated, detubulated cardiomyocytes. **B**, Clustering analysis of single molecule fluorescence in situ hybridization (smFISH) images shows an increase in polarization index and dispersion index for β_2_AR in detubulated myocytes. Polarization of β_1_AR also significantly increased postdetubulation. N=3, n=24 to 41. Data were presented as mean±SD. Statistical significance was determined using 2-way ANOVA with Šidák correction. **C**, Quantified absolute Förster resonance energy transfer (FRET) response (not normalized to saturator) shows imipramine-driven detubulation has a similar effect on β_2_AR receptor function as myocardial infarction by reducing mainly β_2_AR specific cyclic adenosine monophosphate (cAMP) output. β_1_AR: N=3, n=16 to 61. β_2_AR: N=3 to 4, n=19 to 22. Data were presented as mean±SD. Statistical significance was determined using Kruskal-Wallis test.

After confirming the efficacy of 300 μM imipramine in removing t-tubules from isolated ARVM (Figure S6), we performed smFISH experiments on control and detubulated ARVMs (Figure 6A). Clustering analysis of smFISH images revealed that t-tubule remodeling does alter both β-AR mRNA localization patterns in cardiomyocytes. Both β_1_AR and β_2_AR mRNAs PI were increased (Figure 6B), an effect similar to that observed in failing cardiomyocytes. In terms of dispersion, only β_2_AR transcripts’ distribution was altered as the DI increased, in line with failing cardiomyocytes (Figure 6B). However, the dispersion pattern of β_1_AR mRNAs was not statistically significant between control and detubulated ARVMs (Figure 6B), suggesting t-tubule remodeling affects β_2_AR more statistically significantly than its β_1_AR counterpart.

To test whether acute de-tubulation influences membrane β_2_AR function and whether its effect is comparable to that of failing cardiomyocytes, we performed a FRET microscopy experiment using the membrane-localized FRET sensor pmEpac2, on both control, 300 μM imipramine treated, and failing ARVMs. Unsurprisingly, de-tubulation significantly reduced β_2_AR response on the cell membrane, similar to failing cardiomyocytes (Figure 6C). It should be pointed out that the total amount of cAMP available in the cell is not affected by de-tubulation treatment but is lower in failing cardiomyocytes (Figure 6C), and thus, the reduction in FRET response observed was due to altered β_2_AR function on the membrane rather than changes to the total cAMP level available in the cell at that membrane compartment.

## DISCUSSION

In this study, we demonstrated that β_1_AR and β_2_AR-mRNAs are differentially localized in cardiomyocytes, suggesting possible differences in the membrane receptor trafficking mechanisms. We have also found that β_2_AR, but not β_1_AR, mRNA is transported in cardiomyocytes via the microtubule network. While previous studies^27,28^ demonstrated that several cardiomyocyte mRNAs are locally translated into proteins, we have shown that not all mRNAs behave in this manner. This suggests that in ARVM, some, but not all, mRNAs are translated into proteins far from the nucleus, consistent with recent observations in murine cardiomyocytes.^33^

Various studies have demonstrated that compartmentation of β_2_AR induces a cAMP response in ARVMs,^40–44^ and in 2010, one of our previous studies demonstrated the redistribution of functional β_2_AR receptors on the plasma membrane and the loss of compartmentation; yet, the mechanism controlling this remained elusive at the time.^16^ This study shows that, unlike β_1_AR, β_2_AR mRNA is localized throughout the whole cell. In addition, we found that β_2_AR mRNA colocalized with its translated protein to a higher degree compared with β_1_AR. We also found that, at the protein level, β_2_AR showed a higher colocalization with the microtubule network than β_1_AR. Taken together, this suggests that β_2_AR is likely to be locally translated into functional receptors as shown for some other proteins.^27,28^

In neurons, where localized translation of protein is extensively studied, mRNA compartmentalization around the synapse and targeted, localized translation facilitate rapid physiological changes at the microdomains.^25,26^ In the case of cardiomyocytes, however, this was not known to be the case. Although emerging evidence has suggested that various mRNAs are found throughout cardiomyocytes, and their transport is microtubule-dependent, our data have further demonstrated that some mRNAs, such as β_1_AR and β_2_AR differ in the way they are targeted for translation. The former could be mainly trafficked as translated proteins, whereas the latter could be translated in dedicated cellular compartments after the respective mRNA has been trafficked there. It is possible that these 2 functionally and structurally very similar receptors evolved to perform their function in different compartments by means of differential mRNA localization and subsequent translation of their products.

We are the first to show that mRNA molecules and mRNP destination sites are colocalized with the microtubules within the cell. This suggests that in cardiomyocytes, some mRNAs, such as β_2_AR, are packaged into messenger ribonucleoproteins and are translated into proteins as they are transported to their final destination via microtubules. This idea is supported by a previous study, where the authors found that the protein synthesis machinery, namely the Golgi apparatus and endoplasmic reticulum, is scattered throughout a cardiomyocyte.^33^ In the same study, the authors also revealed that in cardiomyocytes, ribosome-associated mRNAs are distributed throughout the cell. They activated the mTOR pathway to increase transcription and protein synthesis. It was found that by doing so, there was a global increase of ribosome-associated mRNAs throughout the cell without any changes in their distribution.^33^ This finding further supports the idea that cardiomyocyte mRNAs may be translated into functional proteins as they are being transported along the microtubule network toward their final destination.

Our FRET data provided additional evidence supporting this idea. Depolymerization of microtubules caused a significant downregulation of β_2_AR-induced cAMP release on the plasma membrane, whereas the β_1_AR receptor function was not statistically significantly affected, indicating there is a downregulation in β_2_AR but not β_1_AR receptor number on the cell surface when microtubules are disrupted. We have also found that neither β_1/2_AR protein localization pattern nor their function on the membrane compartments were regulated by the actin network, which explains why the presence of actin depolymerizing agent cytochalasin D did not affect β-adrenergic mediated cAMP response at multiple subcellular nanodomains in cardiomyocytes.^45^ Our study focused predominantly on mechanisms regulating membrane localization of β_1_AR and β_2_AR in cardiomyocytes. The role of microtubules in redistributing these proteins to other cellular compartments is beyond the scope of this study and will require further investigation.

In failing cardiomyocytes, β_1_AR and β_2_AR-mRNAs became more polarized (having traveled further away from the nucleus) and more dispersed (less clustered in relation to each other). We have also noticed that acute de-tubulation using imipramine led to β_2_AR mRNA redistribution, a pattern that was also observed in failing cardiomyocytes. To investigate whether acute de-tubulation in healthy cardiomyocytes could alter the amount of functional β_2_AR proteins in the plasma membrane, we performed FRET microscopy on healthy, detubulated, and failing cardiomyocytes. The results show that detubulation indeed had a similar effect as myocardial infarction on membrane-localized β_2_AR at a functional level. The results suggest that t-tubule remodeling is likely to have contributed to the redistribution of the β_2_AR mRNA, hence a physical redistribution of the translated proteins and altered β-AR mediated cAMP signaling pathway in failing ARVMs. A 2014 study has revealed the link between microtubule densification in failing hearts leading to t-tubule remodeling in pressure-overloaded murine hearts due to a defective transport of juntophilin2, a protein that anchors the t-tubules to the sarcoplasmic reticulum membranes.^36^ It is, therefore, likely that in failing hearts, there is the densification of the microtubule network in cardiomyocytes, leading to insufficient or defective transport of proteins toward the cell periphery. This leads to t-tubule remodeling and subsequent redistribution of β-AR mRNAs and proteins, causing the loss of compartmentation of β_2_AR-mediated cAMP signaling.^16^ β_2_AR then became predominantly associated with the stimulatory G-protein and further drives pathological phenotype in a positive feedback loop. This may be even more exacerbated by the remodeling of the phosphodiesterases.^46^

To conclude, differential β_1_AR and β_2_AR localization in healthy cardiomyocytes are dependent on the asymmetrical microtubule-dependent trafficking of mRNA (for β_2_AR) for or protein (for β_1_AR), underlying the distinctive compartmentation of the 2 beta-adrenergic receptors on the plasma membrane. Following myocardial infarction, the localization pattern of beta-adrenergic receptor alters, partly due to the t-tubule remodeling and microtubule restructuring, eventually leading to distorted β2AR-mediated cAMP signaling.

## ARTICLE INFORMATION

### Acknowledgments

The authors thank the Facility for Imaging by Light Microscopy and the Center of Excellence Cellular Mechanosensing and Functional Microscopy at Imperial College London.

### Sources of Funding

This work was supported by the British Heart Foundation grant (RG/F/22/110081 to J. Gorelik/J.L. S.-A.). A.P. Ivanov and J.B. Edel acknowledge support from EPSRC grants EP/P011985/1, EP/V049070/1, and Analytical Chemistry Trust Fund grant 600322/05. This project has also received funding from the European Research Council (ERC) under the European Union’s Horizon 2020 research and innovation program (grant agreement nos. 724300 and 875525). B. Wojciak-Stothard acknowledges BHF grant PG/19/19/34286. B.P. Nadappuram acknowledges support from the Analytical Chemistry Trust Fund and Community for Analytical Measurement Science fellowship (Ref. No. 600310/21/07).

### Disclosures

None.

### Supplemental Material

Expanded Material and Methods

References 47–53

Figures S1–S7

Supplementary Scripts

Data and Statistics

Major Resource Table

## Supplementary Material


