# Supplementary material for: Microtubule-Mediated Regulation of β2AR Translation and Function in Failing Hearts
Source: Circ Res. 2023 Oct 23;133(11):944–58. doi: 10.1161/CIRCRESAHA.123.323174 (PMC10635332; doi:10.1161/CIRCRESAHA.123.323174)
Supplement: Supplementary file 1 [file res-133-944-s001.pdf]

Supplemental Material

Microtubule-mediated regulation of β2AR translation, localization and function in failing hearts

Zoe Kwan, Binoy P. Nadappuram, Manton M. Leung, Alexander Kondrashov, Sanika Mohagaonkar, Ao Li, Kumuthu S. Amaradasa, Ji Chen, Stephen Rothery, Iyobel Kibreab, Jiarong Fu, Jose L. Sanchez-Alonso, Catherine A. Mansfield, Hariharan Subramanian, Peter T. Wright, Pamela Swiatlowska, Viacheslav O. Nikolaev, Beata Wojciak-Stothard, Aleksandar P. Ivanov, Joshua B. Edel, Julia Gorelik

METHODS

Animal model

All animal surgical procedures and perioperative management were carried out in accordance with the United Kingdom Home Office Guide on the Operation of the Animals (Scientific Procedures) Act 1986 and EU Directive 2010/83, under the approval of the Animal Welfare and Ethics Review Board (AWERB) of Imperial College London. Myocardial infarction by proximal coronary ligation was used as a model for heart failure in adult rats as previously described[1]. In brief, coronary ligation was performed on randomly selected male Sprague-Dawley rats to generate transmural infarcts and induce chronic heart failure. 16-24 weeks post-infarction, the infarcted heart significantly increased in heart weight/body weight ratios (g/kg) and the heart showed significantly reduced ejection fraction and elevated end-diastolic pressure, suggested hypertrophy of the heart as well as decreased cardiac output and function[20]. Animals that failed to reach 16 weeks post-infarction due to surgery complications were excluded from the study. The full exclusion, randomization, and blinding criteria can be found in the major resources table.

Adult rat ventricular myocytes (ARVM) were isolated from male Sprague-Dawley rats using a Langendorff perfusion system as previously described[46]. In brief, male Sprague-Dawley rats weighing 300-500g at around 4-6 months old were anesthetized with isoflurane, followed by euthanasia by cervical dislocation. The heart was excised from the thoracic cavity and placed into ice-cold Krebs-Henseleit (KH) Buffer. The lungs, thyroid, and pericardial fat were removed, the aorta was cannulated, and the heart was perfused with KH buffer at 37°C on a Langendorff apparatus. The high calcium content in the KH buffer maintains the rhythmic beating of the heart while the blood is being cleared. Once the fluid starts to run clear, the heart is perfused with an oxygenated, low calcium buffer (LoCa2+) containing 12-15µM $CaCl_2$, 120mM NaCl, 5.4mM KCl, 5mM $MgSO_4$, 5mM pyruvate, 20mM glucose, 20mM taurine, 10mM HEPES, and 5mM nitrilotriacetic acid. The low calcium content and nitrilotriacetic acid cause the heart to stop beating, protecting cardiomyocytes from hypercontracture while the perfusion process continues for 5 minutes. The heart is then perfused with an oxygenated enzymatic buffer containing 1mg/ml Collagenase II and 0.6mg/ml Hyaluronidase (C+H) in enzyme (Enz) buffer (12-15µM $CaCl_2$, 120mM NaCl, 5.4mM KCl, 5mM $MgSO_4$, 5mM pyruvate, 20mM glucose, 20mM taurine, 10mM HEPES,150µM Ca^{2+}) to digest and isolate the cardiomyocytes from the scaffold

enzymatically. The heart was perfused in this solution for 10 minutes. Once the perfusion had been completed, the heart was transferred into a plate with fresh C+H buffer for further processing. The left ventricles were removed, minced, and transferred into a clean 50mL tube containing clean C+H buffer, and the mixture was shaken at 35°C for 5 minutes. The mixture was passed through a clean gauze to collect the isolated cardiomyocytes supernatant. The remaining tissue was transferred to a falcon tube with more fresh C+H buffer for the second round of digestion when it continued to be shaken for 30 minutes at 35°C. The mixture was once again passed through a clean gauze, and the supernatant was collected. The supernatant containing cardiomyocytes was centrifuged at 700 rpm for 1 minute to obtain the cell pellet, and the supernatant was aspirated. The cell pellet was then resuspended in fresh Enz buffer, ready for further applications. ARVMs isolated using this procedure maintain their rod-shaped morphology, are Ca^{2+} tolerant, and remain primarily quiescent unless under stimulation, which are also well maintained in an *in vitro* environment.

Isolated ARVMs were used directly for other biological studies, either centrifuged to obtain the cell pellet or plated onto laminin-coated plastic or glass bottom dishes for downstream studies. For imaging purposes, cells were plated on either 13 or 25 mm laminin-coated glass coverslips. For live cell experiments, 10,000 ARVMs were suspended in 100µl of plating media (5.37g MEM with Hank's salts (Sigma cat no. M1018-10L), 375 mg $NaHCO_3$, 5mL 200mM L-Glutamine (Gibco cat no. 25030081), 5 mL Antibiotic Antimycotic Solution (Sigma, cat no. A5955-100ML), dissolved in 500mL culture grade water), with 10% fetal bovine serum) plated onto laminin-coated 35mm glass bottom dishes (MatTek, cat no. P35G-1.5-14-C) and incubated in the incubator at 37°C with 5% CO_2 for 40-60 minutes until the cells were well attached. Non-adherent cells were then aspirated and the dishes were replenished with fresh serum-free maintenance media (5.37g MEM with Hank's salts (Sigma cat no. M1018-10L), 375 mg $NaHCO_3$, 5mL 200mM L-Glutamine (Gibco cat no. 25030081), 5 mL Antibiotic Antimycotic Solution (Sigma, cat no. A5955-100ML), dissolved in 500mL culture grade water). For imaging purposes, instead of glass bottom dishes, cells were plated on either 13 or 25 mm laminin-coated glass coverslips placed in a 35 mm plastic culture dish, using the exact sample protocol. Any required drug treatments were performed by directly adding the required drug into the culture media to the culture dishes.

Pharmacological treatments

For microtubule disruption in ARVM, either 2µM vinblastine (Tocris, Cat. No. 1256) dissolved in water or 10µM nocodazole (Tocris Cat no. 12-281-0) dissolved in DMSO was used. To efficiently disrupt the microtubule network from healthy ARVMs, either microtubule disrupting agent was added to culture media for 18 hours at 37°C, 5% CO_2. To remove t-tubules from cardiomyocytes, 300 µM Imipramine hydrochloride (Merck, Product No. I-902) dissolved in water was used. Imipramine was diluted to a working concentration of 300µM in a physiological buffer-modified Tyrode's solution (140mM NaCl, 6mM KCl, 1mM $MgCl_2$, 1mM $CaCl_2$, 10mM HEPES, 10mM glucose, calibrated to pH 7.4) and was incubated with ARVMs for 15 mins at 37°C. Imipramine-induced detubulation protocol was adapted from Bourcier *et al.*[47]. 10 µM Cytochalasin D dissolved in DMSO was used to depolymerize the actin network in ARVMs by 24 hours incubation at 37°C, 5% CO_2.

Molecular biology and single-cell biopsy

Total RNA isolation

Total cellular RNA was extracted from isolated ARVMs using RNAzolRT reagents (Merck, Cat no. R4533-50ML) according to the manufacturer's protocol. In brief, for freshly isolated ARVM, 1mL of cells were pelleted by centrifugation at 300g for 5 mins at room temperature. The supernatant was aspirated, and the cell pellet was lysed in 1 mL of RNAzolRT reagent. For cells plated on laminin-coated dishes, culture media was aspirated, 1 mL of RNAzol RT reagent was used to lyse cells directly in the dish, and the cell lysate was collected in a nuclease-free Eppendorf tube. 400µL of nuclease-free distilled water (Invitrogen, Cat no. 11538646) was added to the cell lysate, and the mixture was vortexed vigorously for 15 seconds to homogenize. The mixture was incubated on ice for 15 minutes. The lysed samples were then spun at 12,000g for 15 mins at 4°C. 1 mL of the supernatant containing the RNA was collected and transferred to a new Eppendorf tube without disturbing the pellet at the bottom of the tube containing DNA, protein, and most polysaccharides. 1 mL of isopropanol was added to each sample and was mixed by gently inverting the tube 5-10 times. RNA was precipitated out of the mixture by incubating on ice for 10 minutes, following centrifugation at 12,000 g for 10 minutes. The pelleted RNA was washed with 75% freshly prepared ethanol, followed by centrifugation at 6,000g for 3 mins at 4°C. Ethanol was aspirated, and the washing process was repeated once. After removing all the ethanol, the RNA pellet was either stored at -80°C or dissolved in 30 µL nuclease-free distilled water for downstream application. RNA concentration was determined on a NanoDrop™ 8000 Spectrophotometer instrument.

Single-cell nanobiopsy and sample preservation

In order to obtain highly localized cellular biopsies from ARVMs, nanotweezers were first pulled from dual-barrel glass capillaries using a laser puller and fabricated by carbon deposition as previously described[39].

Nanoscale biopsies were obtained from different cellular locations (cell ends, side edges, peri-nuclear regions) of single ARVMs using the nanoscale tweezers as previously described[39]. In brief, ARVMs were plated on a laminin-coated 35 mm glass bottom dish. Right before the nanobiopsy experiment, the nuclei of ARVMs were fluorescently labeled using NucSpot Live 488 (Biotum, cat no. BT40081-T) according to the manufacturer's protocol. After nuclear staining, the dishes were taken out of the incubator, and culture media was replaced by a high potassium buffer A (120 mM K-gluconate, 25 mM KCl, 2 mM $MgCl_2$, 1 mM $CaCl_2$, 2 mM EGTA, 10 mM glucose, 10 mM HEPES, calibrated to pH 7.4) to depolarise the cells in order to prevent cell contraction during biopsy. The dish with cells was then mounted onto the stage of an optical microscope (IX71, Olympus) and placed on a vibration-isolated table (PTM51509, Thorlabs). The fabricated nanotweezer was mounted onto a micromanipulator (PatchStar, Scientifica) and placed directly on top of the cells. The nanotweezer was manually lowered into the cell at the cellular regions of interest

chosen under the optical microscope, and the function generator was turned on to trigger the dielectrophoretic (DEP) force. Polarisable subcellular content was extracted at 1MHz, 16V, 20 seconds. The nanotweezer, alongside the trapped cellular content, was then retracted manually, and the tweezer tip was snapped into a 0.2mL PCR tube pre-filled with 5µL nuclease-free water.

The samples were subjected to reverse transcription on the same extraction day to minimize RNA degradation, using SuperScript IV VILO master mix (Invitrogen, Cat no. 11756050) according to the manufacturer's protocol. In brief, to the 5 µL sample, 2 µL SuperScript IV VILO master mix and 2µL nuclease-free water were added, and the solution was mixed by pipetting followed by quick spin down. The samples were then placed into a Primus HT Dual Thermal Cycler PCR machine (MWG AG Biotech). The thermocycling profile is as follows: 25°C for 10 mins (primer annealing), 50°C 10 mins (reverses transcription), and 85°C for 5 mins (enzyme inactivation). The complementary DNA (cDNA) was then stored at -80°C until further analysis.

<u>Reverse transcribed quantitative polymerase chain reaction (RT-qPCR)</u>

TaqMan-based RT-qPCR experiments were performed using TaqMan™ Fast Advanced Master Mix (Applied Biosystem, cat no. 4444557) and TaqMan® Gene Expression Assays probes (ThermoFisher Scientific, cat no. 4331182) corresponding to rat β_1AR (assay ID: Rn00824536_s1), rat β_2AR (assay ID: Rn00560650_s1), and 18S (assay ID: Hs03003631_g1, standard or primer limited for duplexed experiments) respectively. To each well on a MicroAmp™ optical 384-well reaction plate (Applied Biosystems, cat no. 4343370), 5 µl of TaqMan™ fast advanced master mix, 0.5 µl of appropriate TaqMan Assay probes, 2.5 µl nuclease-free water, and 2 µl of either diluted cDNA samples or DNA standards were added to make up of a total reaction volume of 10 µl. The plate was then sealed using a MicroAmp™ optical adhesive film (Applied Biosystems, cat no. 4311971). The plates were spun and loaded into Applied Biosystems QuantStudio 6 Flex Real-Time PCR Systems. The thermocycling profile was set according to the manufacturer's protocol, with 55 amplification cycles. The exported data were analyzed using QuantStudio Real-Time PCR Software v1.3 and GraphPad Prism 8 software.

Single-molecule fluorescent in situ hybridization (smFISH)

smFISH experiments were performed using RNAscope® Multiplex Fluorescent Reagent Kit v2 (Cat. No. 323100, ACD-Bio) according to the manufacturer's instructions. In brief, cells were first fixed in 4% Paraformaldehyde (PFA) for 30 mins at room temperature. Samples were then washed 3 times in 1X PBS, and hydrogen peroxide was added and incubated with cells for 10 mins at room temperature. After rinsing twice in distilled water, protease III (diluted to 1:15) was added, and samples were permeabilized for 15 minutes at room temperature. The protease solution was washed off using 1X PBS, and hybridization probes specific for rat β_1AR (Rn-Adrb1), rat β_2AR (Rn-Adrb2), 18S (Rn-18S), or positive/ negative control probes were added. The samples were incubated with the hybridization probes for 2 hours at 40°C in the hybridization oven. After probe hybridization, samples were washed 2 times in 1X RNAscope buffer, 2 mins each time. Samples were stored overnight in 5X sodium chloride and sodium citrate solution (SSC) buffer. On the next day, the SSC buffer was

washed away using 1X RNAscope buffer, 2 mins twice. Then, signal amplification, fluorophore conjugation, and channel blocking for the hybridized probes were performed according to the manufacturer's protocol. After all the targets were amplified and conjugated with fluorophore, the samples were counterstained with DAPI supplied and mounted onto Epredia™ SuperFrost Plus™ Adhesion slides (cat no. 12312148, ThermoFisher Scientific) using ProLong™ Gold Antifade Mountant (ThermoFisher Scientific, P10144). Images were taken on a Zeiss LSM-780 confocal microscope or a Leica Stellaris 8 microscope.

Integrated co-detection workflow (ICW)

A commercially available RNA-protein co-detection assay (ACD-Bio, RNA-Protein Co-detection Ancillary Kit Cat No. 323180), originally intended for RNA and protein co-visualization in tissue sections, was modified and adapted for RNA-protein co-detection in adherent cells. The Co-detection Antibody Diluent from the RNA-Protein Co-detection Ancillary Kit (ACD-Bio, Cat No. 323180) and the RNAscope™ Multiplex Fluorescent V2 Assay kit (ACD-Bio) were essential for this experiment.

ARVMs plated on laminin-coated coverslips were first rinsed twice using 1X PBS and fixed using 4% PFA at room temperature for 30 mins. Fixed cells were washed thrice using 1X PBS before the addition of hydrogen peroxide supplied in the RNAscope™ Multiplex Fluorescent V2 Assay kit. After 10 minutes of incubation at room temperature, the hydrogen peroxide was washed away by rinsing the sample twice in distilled water. Following endogenous peroxidase inactivation, the samples were permeabilized using 0.1% Triton-X (diluted in 1X PBS) for 30 minutes at room temperature. Cells were washed 3 times and then incubated with β_1AR or β_2AR primary antibodies (AAR-023 or AAR-016 respectively, alomone labs) diluted in Co-detection Antibody Diluent at 1:100 dilution (ACD-Bio, Cat No. 323180) at 4°C overnight. After overnight incubation, samples were washed 3 times in 1X PBS and fixed with 4% PFA. After antibody fixation, the cells were further penetrated by protease III treatment (supplied in RNAscope™ Multiplex Fluorescent V2 Assay kit, diluted 1:15 in 1X PBS) for 15 mins at room temperature. The protease solution was removed by washing 3 times in 1X PBS, and the samples were subjected to standard RNAscope™ Multiplex Fluorescent V2 Assay as described in the previous section.

After the final ISH HRP blocker step of the RNAscope protocol, AF488- or AF546-conjugated secondary goat anti-rabbit antibody was diluted to 1:1000 (ThermoFisher) in Co-detection Antibody Diluent and were incubated with the sample at room temperature for 30-60 minutes. Cells were washed 3 times in 1X PBS to remove unbound antibodies, and cell nuclei were stained with DAPI. Finally, the samples were mounted onto Epredia™ SuperFrost Plus™ Adhesion slides (cat no. 12312148, ThermoFisher Scientific) using Citifluor anti-fadent AF1 mountant (AGR1320, Agar Scientific). Images were taken on a Leica Stellaris 8 microscope and deconvoluted using the microscope's built-in Stellaris Lightening platform.

Immunofluorescence (IF) staining

Isolated ARVMs were fixed with 4% PFA in phosphate-buffered saline (PBS) for 15 mins at room temperature. Fixed cells were washed thrice using 1X PBS before the addition of 0.1% Triton-X in PBS for penetration at room temperature for 15 minutes. Cells were washed 3 times in 1X PBS. Samples were blocked in blocking buffer (1%BSA with 5% filtered goat serum in PBS) supplemented with 0.1% Triton-X for 1 hour at room temperature. Cells were washed thrice, then β-tubulin primary anti-mouse (1:1000 clone TU27, Cat No 903401; BioLegend), β-tubulin primary anti-rabbit (1:200, Cell signaling, #2128), MAP4 mouse primary antibodies (1:200, SantaCruz, sc-390286), and/or rabbit primary antibodies against β_1AR or β_2AR (AAR-023 or AAR-016 respectively 1:100, alomone labs), diluted in blocking buffer, were added to the samples for overnight incubation at 4°C. After overnight incubation, samples were washed 3 times in 1X PBS, and secondary antibodies (donkey anti-mouse AF488 and goat anti-rabbit AF546, both 1:1000, ThermoFisher Scientific) or AF546 Phalloidin (1:600, TermoFisher Scientific) diluted in blocking buffer were added to the cells and incubated at room temperature for 1 hour. Lastly, the samples were washed in 1X PBS 3 times and counterstained with DAPI before mounting onto glass slides using ProLong™ Gold Antifade Mountant (ThermoFisher Scientific, P10144) or Citifluor anti-fadent AF1 mountant (AGR1320, Agar Scientific) for samples require deconvolution.

Images were taken on either a Zeiss LSM-780 confocal microscope or a Leica Stellaris 8 microscope. Deconvolution was performed using the Leica Stellaris 8 microscope's built-in Stellaris Lightening platform. The lack of non-specific labeling was confirmed using only secondary antibodies for negative control on control adult rat cardiomyocytes following the same protocol. Best illustrative images were selected as representative images. For randomization, sample preparation, sample imaging, and image analysis were performed by at least 2 different persons in parallel.

Image Analysis

Fiji macro for clustering analysis of smFISH images

smFISH images were analyzed using in-house analysis software, modified based on a previously published algorithm utilizing the concepts of polarisation and dispersion indexes[38,48]. The details on the algorithm and mathematics are explained in detail in the result section of the article. The macro script can be found in the supplementary script PI_DI Analysis. The general workflow for operating the macro is as follows:

1. Drag the image into ImageJ/FIJI

    - The macro can handle both multi-channel and multi-channel z-stack images

2. Drag the macro into ImageJ/FIJI and push "RUN"

3. Input the number of expected nucleus in the pop-up window

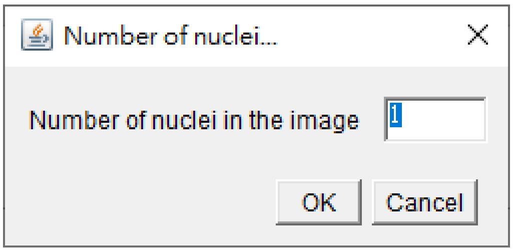

4. Select the nucleus boundary (wand tool is recommended; the macro has pre-set the threshold as 2000

- "0/2" means two nuclei are expected, zero of the nucleus has been selected channel z-stack images

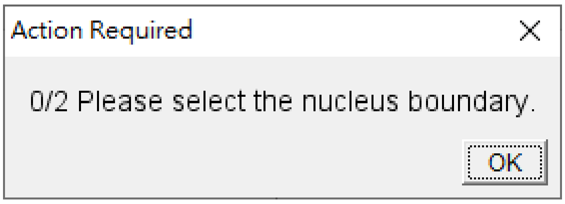

5. Select the cell body (wand tool is recommended; the macro has pre-set the threshold as 60000).

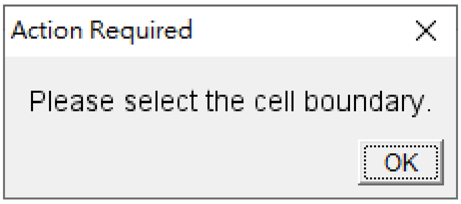

6. Scroll to the channel you want to analyze

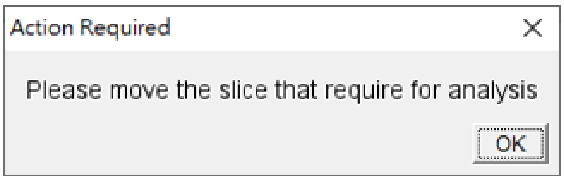

7. Set prominence

- The larger the number, the larger the pixel intensity difference to accept a local maxima (less points selected)
- Input a number and push OK for preview
- Check the box if the prominence gives the desired point selection

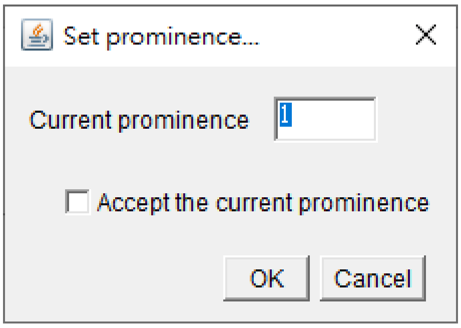

8. Sit back and read the results…

- Log window: Records all the useful information (PI, DI etc.)
- Results window: Records all the positions, nucleus grouping, and distance to their immediate nucleus for individual detected point

- ROI window: Records all the ROI generated during calculation, such as cell centroid, nucleus centroid, and RNA cluster centroid

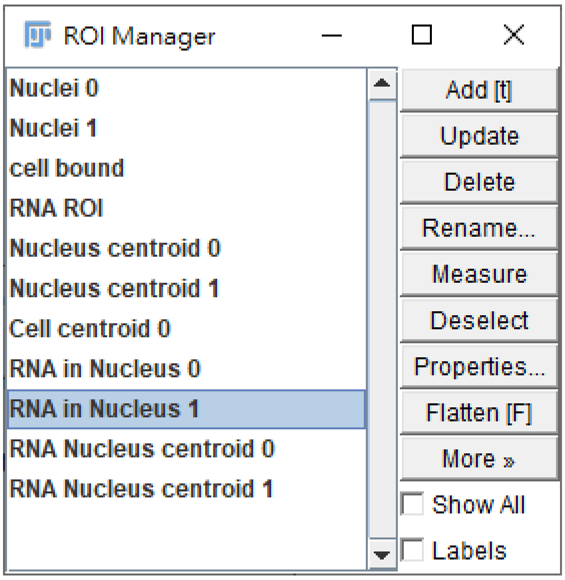

Analysis approach for ICW

In order to analyze the localization pattern between fluorescently labeled RNA and proteins of interest, a distance analysis approach was utilized, and a Fiji macro was created to automate the analysis process. In the Fiji macro script, each RNA signal dot was considered as a particle, and the translated protein as an object. The program automatically detects the labeled RNA and protein signal based on the threshold set by the user. The macro then analyses the images in 3-dimention and calculates the distance between a detected particle (RNA) and its closest object (protein). The results were exported into Excel for further analysis, where the number of particles (RNA) with a distance <0.1 µm to its closest object (protein) was considered as 'colocalized '[2]. Particles found at ≥ 0.1 µm from its closest object were considered as 'not colocalized'. The percentage of colocalization was therefore calculated as:

$$\% \, Localised = \frac{number \; of \; particles \; colocalised \; with \; protein}{total \; number \; of \; particles \; detected}$$

This Fiji macro script can be found in the supplementary script ICW_distanceAnalysis

T-tubule density and regularity analysis

T-tubules of control and imipramine-treated ARVMs were fluorescently labeled using 10µM Di-8-ANEPPS. In brief, 100X Di-8-ANEPPS stock was diluted in modified Tyrode's solution to 1X working concentration, and the mixture was sonicated at 'sweep' mode on a FB15047 sonicator (Fisher Scientific) at 55°C for 20 minutes. The sonicated solution was then added to plated cardiomyocytes, control or drug-treated, for 3 minutes, protected from light. Excess dye was washed twice using 1XPBS, and the t-tubule of the labeled cells was visualized on a Leica Zeiss LSM780 confocal microscope.

T-tubule density of control and/or imipramine-treated ARVMs were quantified using Fiji (https://imagej.net/software/fiji/downloads). First, an image was imported into Fiji and was adjusted into an 8-bit and 1024x1024-pixel image. The image is then rotated until the long axis of the cell under study is in a horizontal position for the ease of t-tubule selection. An ROI of 170.5(W) x 21.5(H) in pixel is then selected, duplicated, and thresholded to identify the fluorescently labeled t-tubules. The percentage of area with fluorescent signal was then calculated. The intensity plot was exported as a .dat file. The exported file is then imported into Matlab software (ver. R2022a, https://uk.mathworks.com/products/matlab.html) and analyzed using the t-tubule calculation program created by Cesare M. Terracciano and Michael Ibrahim as previously published[49]. The y-coordinate of the highest peak was recorded as the quantified t-tubule regularity index. 5 ROIs were randomly selected from each cell for analysis.

Protein density and regularity analysis

β-AR density was quantitively analyzed by Fiji, while βAR regularity was quantitively analyzed using Matlab (ver. R2022a). Images obtained from the confocal microscope were adjusted to 8-bit and 1024*1024 pixels first, followed by further rotation adjustment till the long axis of the cells was roughly parallel with the horizontal line through Image J. A region within cells whose pixels are 170.5 in width and 21.5 in height for a 1024*1024 image was specified. Once the specified region was duplicated, Li's auto-threshold would be applied to the region. The ratio of the area with fluorescent signal to the whole area is the percentage density of β receptors. The plotting data was also saved in '.dat' format and was analyzed via MATLAB with the regularity calculation program created by Cesare M. Terracciano and Michael Ibrahim as previously published[49]. The maximum Y-axis value of the periodogram represents the peak power of T-tubule regularity. Data were collected from 3 to 5 different regions within one cell and presented as mean±SD.

Plasma membrane-localized βAR density was measured using Fiji. The plasma membrane compartment was defined as the edge of the cell. A line was drawn by tracing the edge of the cell, and the fluorescent intensity was measured. One line was measured for each image, and the 3 middle images of the stack of cells were measured. The average value was calculated in Excel. 3-5 cells were measured for each animal, and 3-5 animals were used for each experimental group. Statistical significance was determined by either a normal T-test or the Mann-Whitney U test.

Radioligand binding experiment

Freshly isolated ARVMs were plated on laminin-coated 6 well plates and treated for 18 h either with vehicle or with 2 µM vinblastine. Cells were then disrupted using an Ultra-Turrax device in a buffer containing 5 mM Tris and 2 mM EDTA (pH=7.4), and the membrane (total microsomal fraction) was isolated using ultracentrifugation and resuspended in 50 mM Tris buffer (pH=7.4). Radioligand binding studies were performed as previously described[52]. Briefly, isolated cell membranes (10 µg total protein per sample) were incubated for 2 h at room temperature with 60-100 pmol/L of [3H]-CGP12177 (PerkinElmer Life Sciences, Dreieich, Germany) and increasing

concentrations of ICI118551 to assess the specific binding and relative ratio of beta2/beta1-AR expression. We used a protocol adapted from Kapiloff et al. (PMID: 11590243) for the subcellular fraction with slight modifications. Untreated and vinblastine-treated cardiac myocytes were homogenized in the buffer containing 20 mM HEPES, 1 mM DTT, 0.32 M sucrose, protease, and phosphatase inhibitors (Roche). The homogenates were spun at 20,000 g for 20 min. The supernatant was spun at 100,000 g to collect the microsomal fraction. The microsomal fraction was resuspended in the homogenization buffer, layered over the sucrose gradient (8 parts 24%, 6 parts 40%, 2 parts 50% sucrose), and spun at 100,000 g for 90 min to separate plasma membrane fraction from SR.

Förster resonance energy transfer (FRET) microscopy

High-throughput FRET microscopy (MultiFRET) was performed on live ARVM transduced with plasma membrane-localized cAMP biosensor pmEpac2 as previously described[50,51] using adenoviruses. The pmEpac2 FRET biosensor has a domain that was designed to bind to the adenylyl cyclase 8 on the plasma membrane (supplementary figure1A), making it a good FRET biosensor for monitoring membrane localized cAMP activity[3].

In brief, healthy or failing isolated ARVMs were first plated onto laminin-coated 35mm glass bottom dishes as described in previous sections. The cells were then transduced with a plasma membrane localized cAMP FRET biosensor pmEpac2 using adenoviruses, at a multiplicity of infection (MOI) of 500 in serum-free maintenance medium, for 2 days at 37°C, 5% CO_2. For experiments where drug treatment was required, appropriate drugs were added directly to the culture medium without changing, after adenoviruses were added and before the imaging.

To perform FRET microscopy experiments, the culture medium was first aspirated, and the cells were washed twice with FRET buffer (144mM NaCl, 10mM HEPES, 1mM $MgCl_2$, 5mM KCl). 1mL of buffer A was then added to the cells, and the dish was mounted onto the microscope. The MultiFRET software (Java plugin) was operated by the Icy software (https://icy.bioimageanalysis.org/) as previously described[50]. After cell selection, the MultiFRET was run, and any changes in FRET/ fluorescent signals of all selected cells were recorded in real-time.

The software was set to capture an image and record the fluorescent signal every 12 seconds, and the fluorescent is recorded for ~20 frames before the next reagent is added. Once the baseline signal was stabilized, β-AR selective blockers (β_1 blocker 100nM CGP 20712 A, or β_2 blocker 50nM ICI 118551) were added to the dish. Then, the non-selective β-agonist isoproterenol (ISO, 100 nM) was added, and the change in FRET signal was recorded automatically. A saturator solution (100 µM IBMX and 10 µM forskolin) was then added to trigger the maximum cAMP response, which was once again recorded automatically. The program was terminated once the saturator response had plateaued. The recorded fluorescent data and automatically calculated FRET ratio shift were exported for further analysis.

The FRET ratio shift was calculated as:

$$FRET\ ratio\ shift = \frac{(Average\ baseline\ FRET\ signal - average\ ISO\ triggered\ FRET\ response)}{Average\ baseline\ FRET\ signal}$$

The percentage cAMP response normalised to saturator was then calculated as:

$$\%\ cAMP\ response = \frac{FRET\ ratio\ shift\ of\ ISO\ response}{FRET\ ratio\ shift\ of\ saturator\ response} \times 100\%$$

Statistics

All statistical analysis and graphs were performed/ generated using GraphPad Prism 8. The Shapiro-Wilk test was used to test for dataset normality in all statistical tests performed. The statistical test used for each data set is indicated in each figure legend. If the data presented in this work failed to pass the normality test, the corresponding non-parametric test was used. $p<0.05$ was considered statistically significant. N = number of biological replicates (i.e., number of animals) and n = number of technical replicates (i.e., the number of cells) analysed. Dataset that fulfils the assumption for hierarchieal analysis are analyzed using “nested” tests. Unless specified to be a nested analysis, all statistical tests treats each cell analyzed as a single datapoint. Each animal or biological replicate is represented by a different shape on the presented dot plots.

SUPP. FIGURES

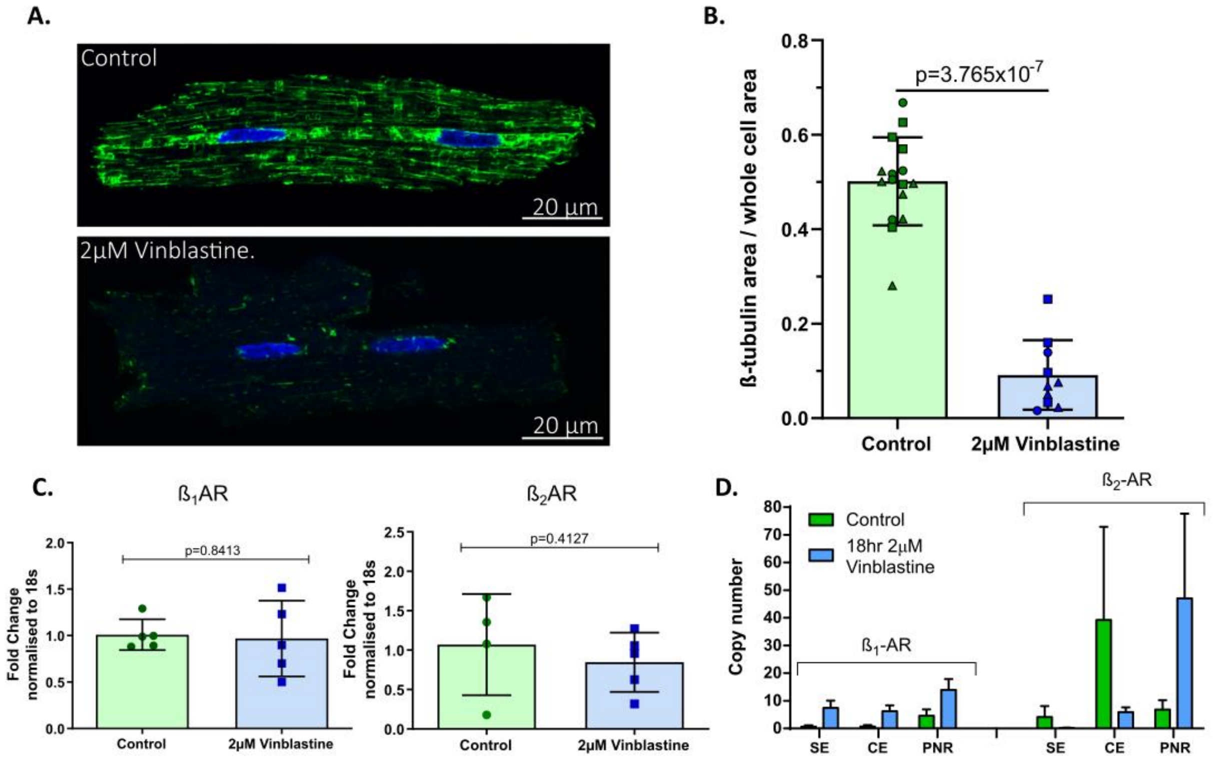

Supplementary figure 1. (A) beta-tubulin staining in control versus 18 hours 2µM vinblastine treated cardiomyocytes. (B) beta-tubulin quantification in control versus vinblastine-treated cardiomyocytes. N=3, n=12-16. Statistical significance was determined by an unpaired t-test. (C) RT-qPCR analysis of total $ß_{1/2}$AR expression in control versus 18 hours 2µM vinblastine treated cardiomyocytes. N=4-5. Statistical significance was determined by the Mann-Whitney U test. (D) The bar chart represents the number of copies of $ß_{1/2}$AR mRNA found in different regions of cardiomyocytes in control versus 18 hours 2µM vinblastine treated cardiomyocytes. SE = side edge, CE = cell edge, and PNE = perinuclear region. N=3-5, n=11-26. All data are represented as mean ± SEM.

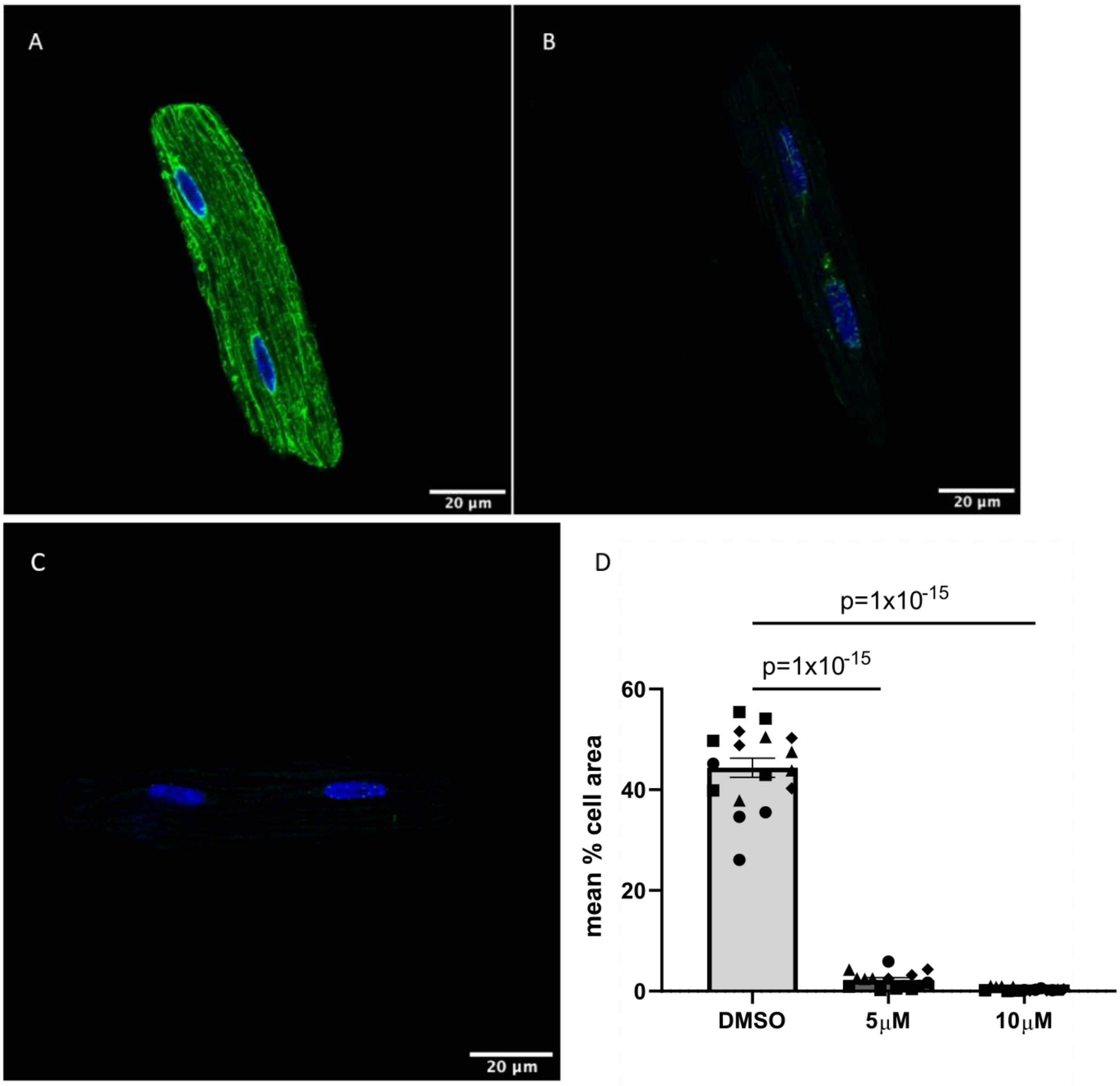

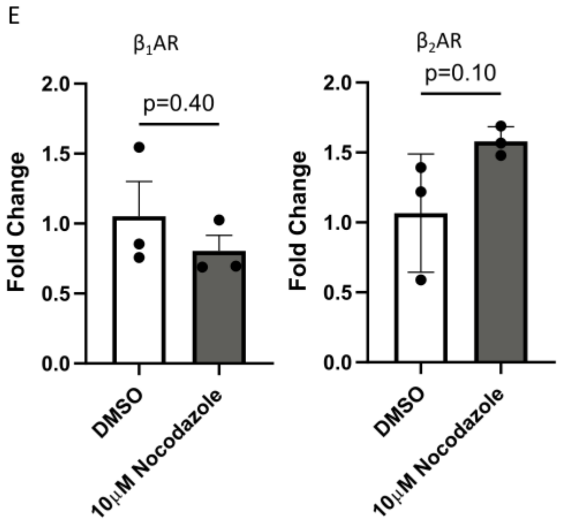

Supplementary figure 2. Representative image of β-tubulin immunostaining in (A) DMSO, (B) 5µM Nocodazole, and (C) 10µM Nocodazole treated cardiomyocyte. (D) Mean percentage of tubulin to cell area of cardiomyocytes treated with DMSO, 5µM, and 10µM after 18 hours (N=4, n≥3). Statistical significance was determined by one-way ANOVA test with post-hoc Dunnett's multiple comparison test with data represented as mean ± SEM. (E) Gene expression level of $\beta_{1/2}$AR in control and 10µM nocodazole-treated cardiomyocytes. Statistical significance for β_1AR was determined by the Mann-Whitney U test and for β_2AR by Welch's unpaired t-test. Data represented as mean ± SEM.

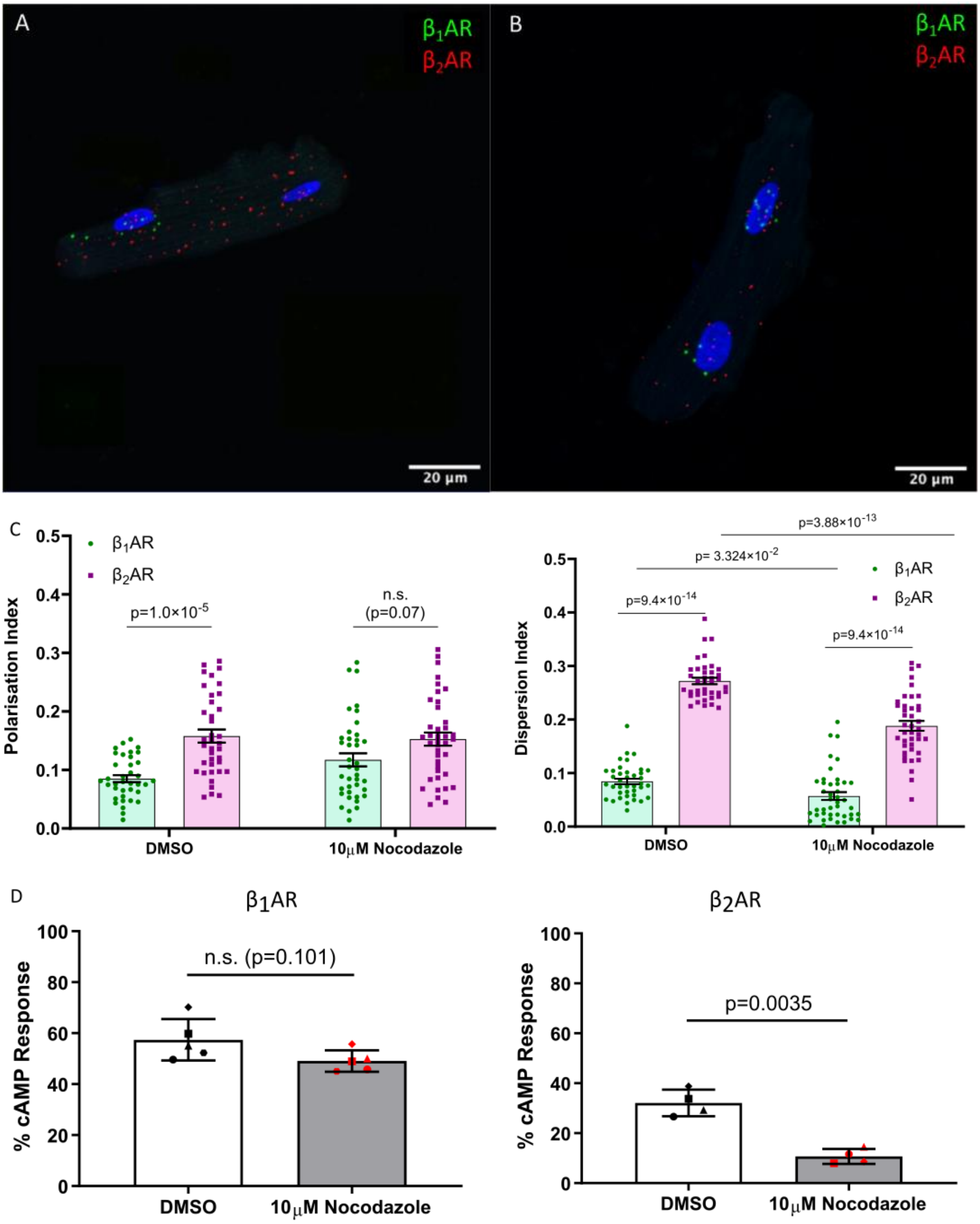

Supplementary figure 3. Representative smFISH image (A) control and (B) 10µM nocodazole treated cardiomyocytes. (C) clustering analysis of smFISH images described as polarisation Index and dispersion index. (N=4, n=39-41). Two-way ANOVA determined statistical significance with post-hoc Tukey's multiple comparison test (DI) or Sidak's multiple comparisons test (PI) with data presented as mean ± SEM. (D) % cAMP response of β_1AR and β_2AR normalized to the saturator response (N=4-

5, n=3-17). Statistical significance was determined via nested t-test, and data was presented as mean ± SEM.

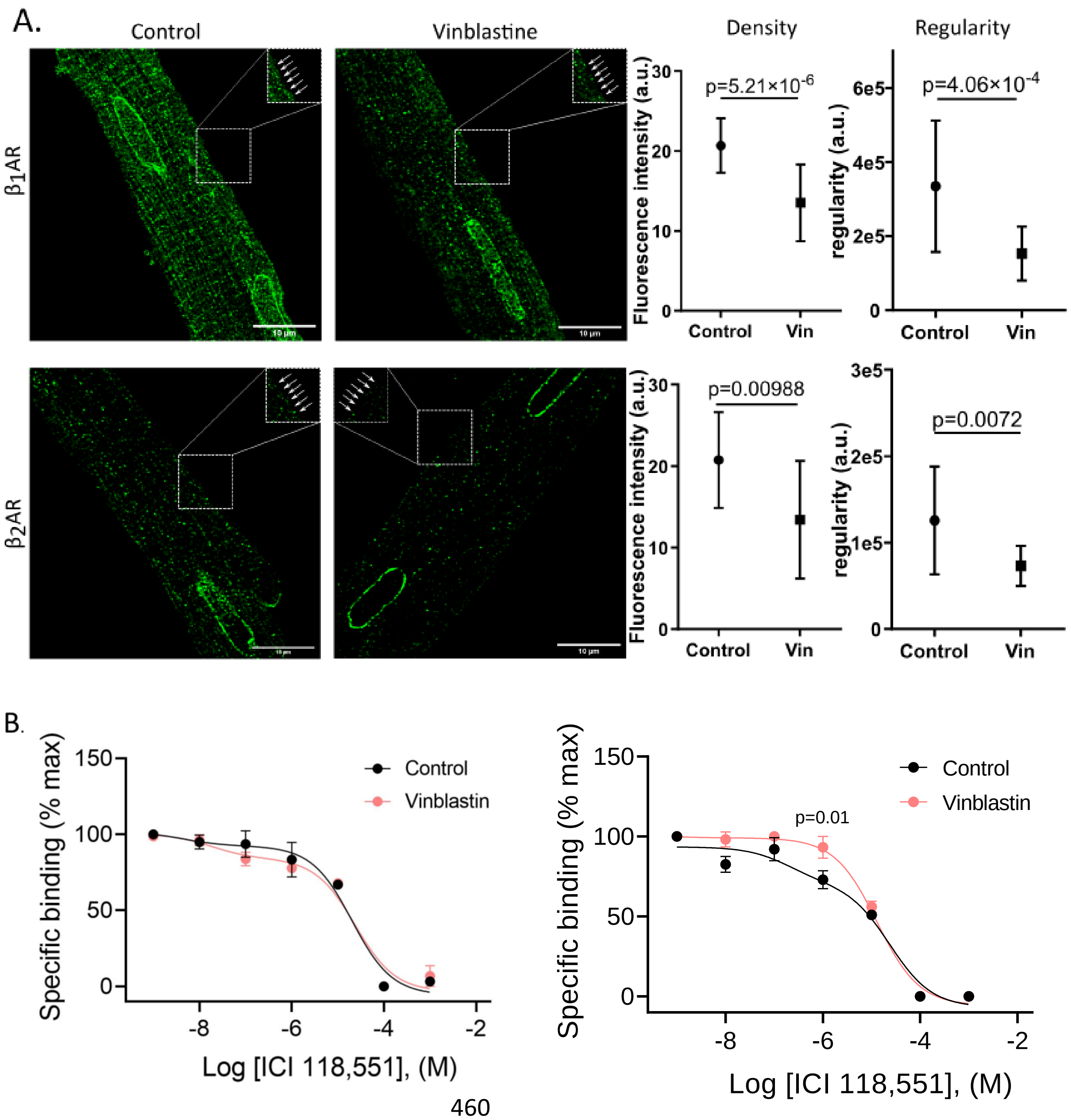

Supplementary figure 4. (A) $\beta_{1/2}$AR protein staining in control and/or 18 hours vinblastine treated cardiomyocytes. Left panel: Representative fluorescent images. Right panel: Protein density and regularity analysis. Data was presented as mean ± SD. N=3-5, n=10-20. Statistical significances were determined by unpaired t-test with Welch's correction or Mann-Whitney U test (β_2AR regularity). (B) Radioligand binding assay for β_1- and β_2-AR receptors in all membrane compartments (left panel) and the plasma membrane compartments (right panel) in cardiomyocytes. The first minor ligand displacement component indicates the β_2AR fraction, the second major

component - the β_1ARs. N=5. Statistical significance was determined by a parametric two-tailed unpaired t-test.

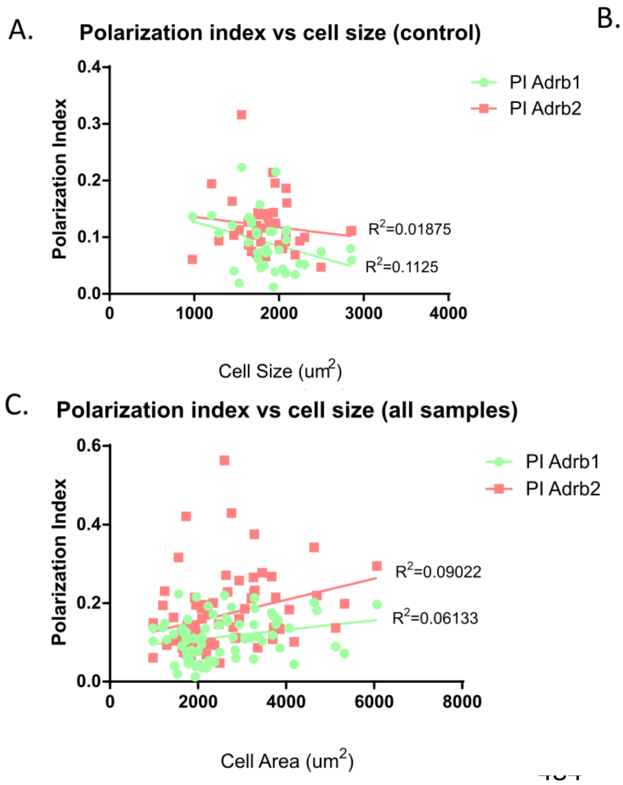

Supplementary figure 5. Correlation between polarization index (PI) and cell area in (A) Control cardiomyocytes, (B) Failing cardiomyocytes, and (C) all cells sampled. There was no direct correlation between cell size and the polarization index generated.

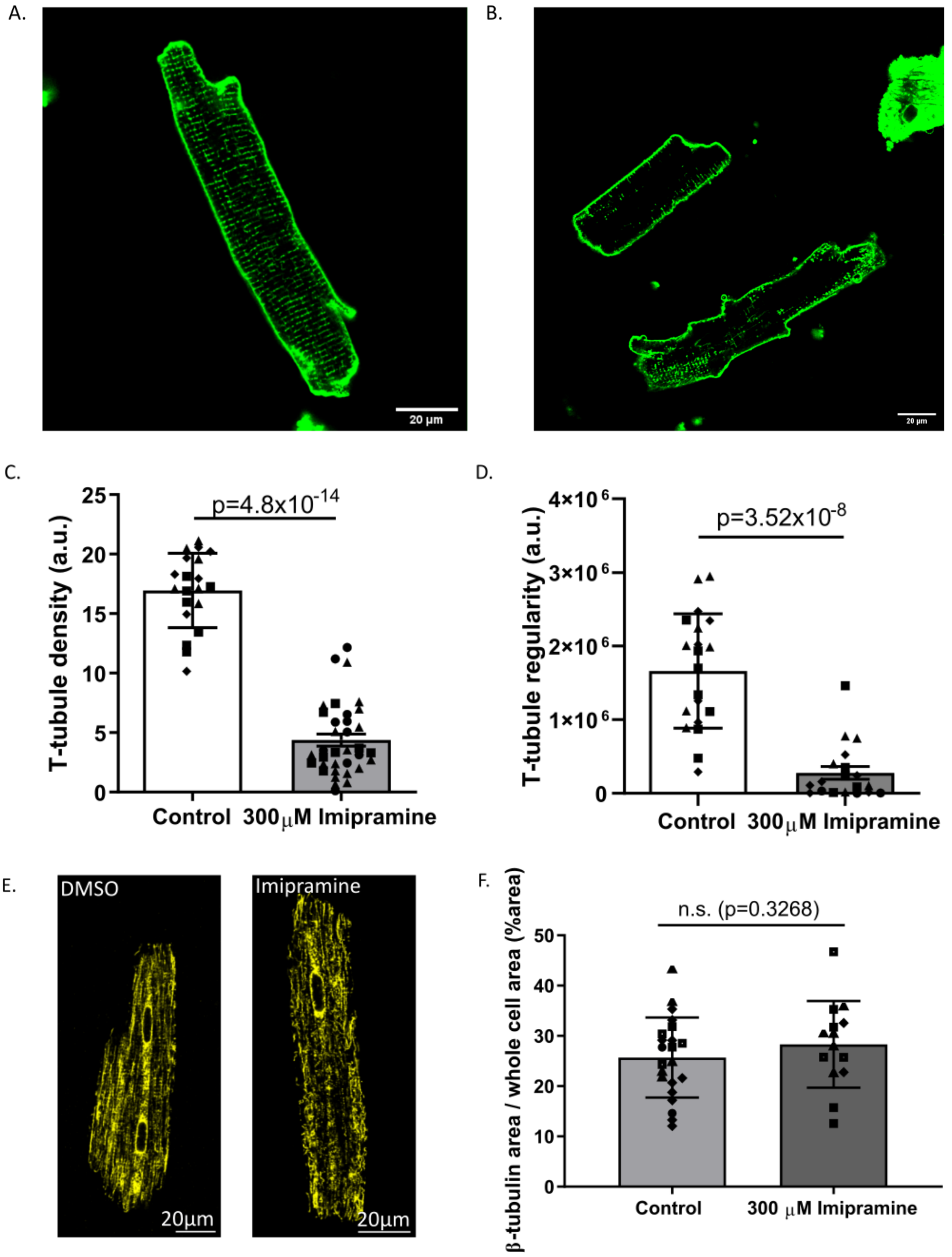

Supplementary figure 6. Validation of Imipramine as a detubulation agent. (A) Representative t-tubule staining in control cardiomyocytes using Di-8-ANEPPS (B) Representative t-tubule staining in 300µM imipramine treated cardiomyocytes using Di-8-ANEPPS. (C) t-tubule density and (D) regularity quantification in control versus imipramine-treated cardiomyocytes. Data was presented as mean ± SEM. N=3-4,

n=20-35. Statistical significance was determined by the Mann-Whitney U Test. (E) Representative fluorescent image of β-tubulin staining in control and imipramine-treated cardiomyocytes. (F) Quantification of β-tubulin as a percentage of tubulin area normalized to cell area. Data presented as mean ± SD N=3, n=14-22

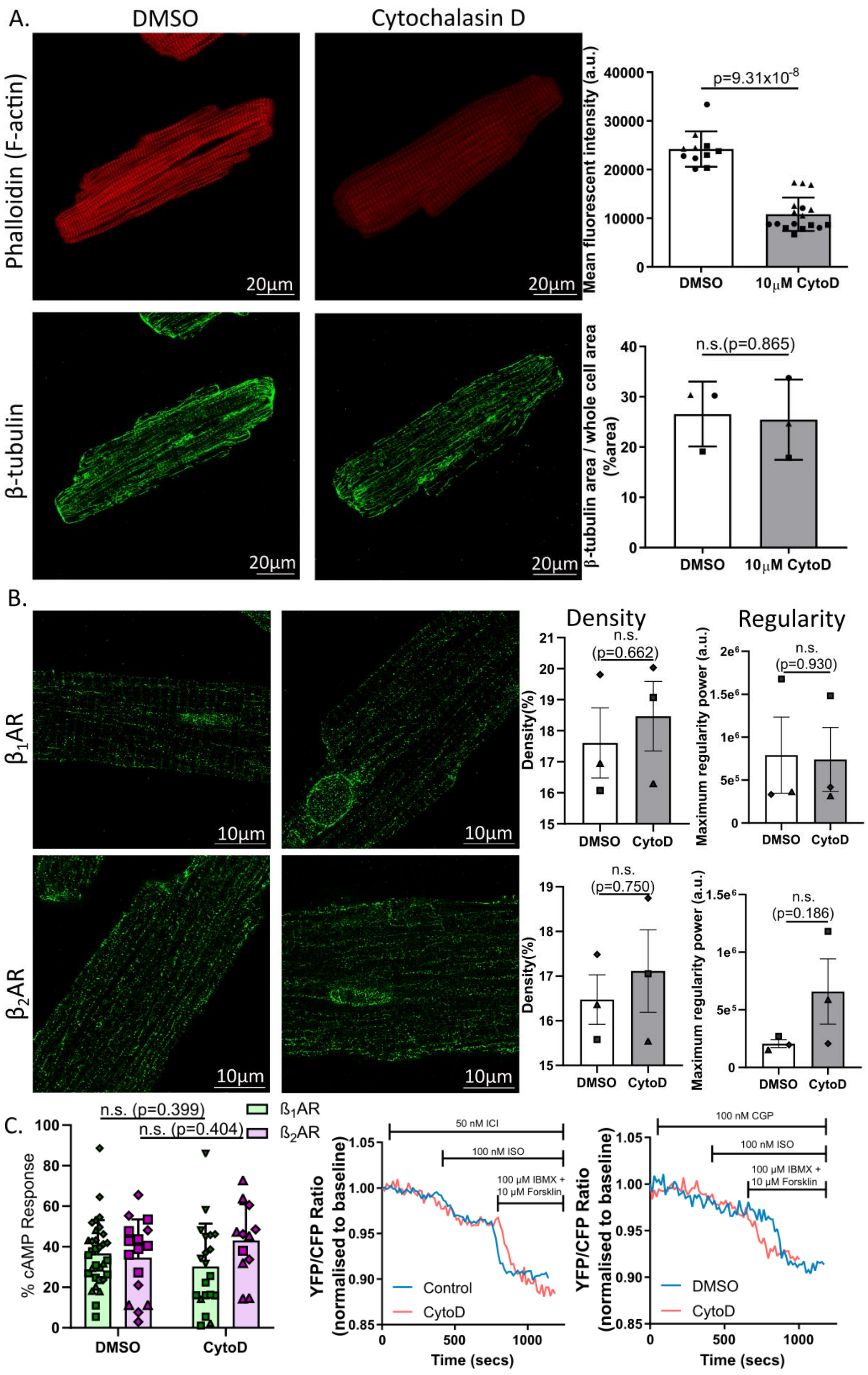
A.
DMSO
Cytochalasin D
Phalloidin (F-actin)
β-tubulin
20μm
Mean fluorescent intensity (a.u.)
p=9.31x10-8
DMSO
10μM CytoD
β-tubulin area / whole cell area (%area)
n.s.(p=0.865)
B.
β1AR
β2AR
10μm
Density
Regularity
n.s. (p=0.662)
n.s. (p=0.930)
n.s. (p=0.750)
n.s. (p=0.186)
Density(%)
Maximum regularity power (a.u.)
DMSO
CytoD
C.
% cAMP Response
n.s. (p=0.399)
n.s. (p=0.404)
ß1AR
ß2AR
50 nM ICI
100 nM ISO
100 μM IBMX + 10 μM Forsklin
YFP/CFP Ratio (normalised to baseline)
Control
CytoD
Time (secs)
100 nM CGP
DMSO

Supplementary figure 7. Actin filament depolymerization did not have statistical significance on βAR localization or function. (A) Representative F-actin and β-tubulin staining and quantification in control versus 10μM Cytochalasin D treated cardiomyocytes. F-actin: N=3, n=11-17. Statistical significance determined by Mann Whitney U test. β-tubulin: N=3, n=13-16. Statistical significance determined by nested t-test. (B) Representative βAR immunostaining and density/ regularity quantification in control versus 10μM Cytochalasin D treated cardiomyocyte. All statistical significance was determined by nested t-tests. (C) The effect of 10μM Cytochalasin D treatment of βAR function on the plasma membrane compartment. Left panel: β_1AR and β_2AR specific cAMP response of control and 10 μM Cytochalasin D treated cardiomyocytes, normalized to saturator response; N=3-4, N=15-32. Mixed-model 2-way ANOVA determined statistical significance with Sidak's multiple comparisons test. Representative FRET traces of (middle panel) β1AR and (right panel) β2AR specific cAMP response in control and 10 μM cytochalasin D treated cardiomyocytes. β-AR subtype-specific blocker (β_1- blocker 100 nM CGP 20712 A, β_2- blocker 50 nM ICI-118,551) was added, and the non-selective β-agonist 100 nM isoproterenol (ISO) was used to trigger β-AR subtype-specific response. A saturator (100 μM IBMX + 10 μM Forskolin) was then added to trigger maximum cAMP response of the cell.

SUPP. SCRIPTS

Macro script PI_DI Analysis

Fiji macro script used for clustering analysis of smFISH images.

```
//This macro is developed for estimate the polarization index (PI) and desersion index (DI) of FISH signals (Park et al., 2012). The calculation mentioned in the paper assumes uninucleated cells and single RNA cluster. Here, the FISH signals are first clustered with regarded to their immediate nucleus. Then, the PI and DI are calculated regarding to their coresponding nucleus centriods (instead of the cell centroid). However, the radius of gyration of cell is kept for normalizing the cell size factor.

//Indexes for one channel can be calculated each time. For multi-channel, please re-run and select the desire channel. Both Z-stack and single plane images can be handled. If an z-stack image is imported, the macro performs max. projection to all channels to yield a 2D multi-channel image.

//Wand tool is suggested for selection of nucleus and cell boundary. Especially, for cell boundary, you may adjust the tolerance of the wand tool and select using the nucleus (DAPI) channel.

//The FISH signals are detected using FIJI buildin "find maxima..." function. Only area within the cell boundary is considered. Prominence is the pixel difference of a local maxima with 8-contiguous pixels (local maxima - promience).

//This macro provides a user-friendly pipeline for PI and DI calculation. Robust results can be excelled, as minimal input is required from users. Shall you have any question, please feel free to email me at "mantonleung@gmail.com".

//

title = getTitle();

print("~~~~~~~");

print(title);

//image preparation

ImgPreparation();


//get nucleus

nuNumber = AskForNuclei();

nuRoi = GetNucleus(nuNumber);


//get Cell boundary
```

```
run("Wand Tool...", "tolerance=60000 mode=Legacy");
waitForUser("Please select the cell boundary.");
RoiRename("cell bound");
 List.setMeasurements;
 getPixelSize(unit, pixelWidth, pixelHeight);
 print(">>Cell area (" + unit + "^2): \t" + List.get("Area"));
cellRoi = newArray();
cellRoi = Array.concat(roiManager("index"));
centNu = GetCentroid(nuRoi, "Nucleus centroid");
centCell = GetCentroid(cellRoi, "Cell centroid");

while(true){
	//get analyzing slice number and detect pts
	waitForUser("Please move the slice that require for analysis");
	anSlice = getSliceNumber();
	roiName = "Ch " + anSlice + " RNA";
	rnaRoi = GetMaximaPt(anSlice, cellRoi, roiName);

	//Calculations
	rog = GetCellROG(cellRoi, centCell);
	nuRNARoi = Nucleigp(rnaRoi, centNu, roiName);

	roiManager("select", cellRoi);
	Roi.getContainedPoints(xpoints, ypoints);

	for(i = 0; i < nuRNARoi.length; i++){
		//Calculation for PI for each nucleus
		print("Now processing channel " + anSlice +"...");
		string = "Ch "+anSlice+" RNA Nucleus centroid " + i;
		centRNA = GetDotsCentroid(nuRNARoi[i], string);
```

```
			nuPI = CalPI(newArray(centNu[i*2], centNu[(i*2)+1]), centRNA, rog);
			print(">>Channel "+ anSlice+" Polar Index (nucleus " + i + "): \t" + nuPI);
			//Calculation for DI
			print("RNA centroid: (\t"+centRNA[0]+", " + centRNA[1]);
			cell2Moment = SecondMoment(centCell[0], centCell[1], xpoints, ypoints);
			print("Second moment of whole cell: \t" + cell2Moment);
			roiManager("select", nuRNARoi[i]);
			getSelectionCoordinates(rnaXpts, rnaYpts);
			rna2Moment = SecondMoment(centRNA[0], centRNA[1], rnaXpts, rnaYpts);
			print("Second moment of RNA nucleus " + i + ": \t" + rna2Moment);
			di = rna2Moment / cell2Moment;
			print(">>Channel "+ anSlice+" Dispersion Index (nucleus " + i + "): \t" + di);
		}
		Dialog.create("Follow up analysis");
		Dialog.addMessage("Continue processing other channels?");
		Dialog.show();
	}
	print("~~~~~~~\n");

function GetDotsCentroid(index, string){
	roiManager("select", index);
	getSelectionCoordinates(xpts, ypts);
	meanX = 0;
	meanY = 0;
	for(i = 0; i < xpts.length; i++){
		meanX = meanX + xpts[i];
		meanY = meanY + ypts[i];
	}
	meanX = meanX / xpts.length;
```

```
		meanY = meanY / xpts.length;
		makePoint(meanX, meanY);
		RoiRename(string);
		print(string + "\t(" + meanX + ", " + meanY + ")");
		return newArray(meanX, meanY);
}

//grouping RNA to indicidual nuclei according to minimal distance
function Nucleigp(index, centroids, string){
		roiManager("select", index);
		getSelectionCoordinates(rnaXpts, rnaYpts);
		group = newArray(rnaXpts.length); //for storing the group number
		minVal = newArray(rnaXpts.length);
		rois = newArray(centroids.length/2);
		for(i = 0; i < rnaXpts.length; i++){
				min = 999999;
				for(j = 0; j < rois.length; j++){
						temp = Distance(rnaXpts[i], rnaYpts[i], centroids[j*2], centroids[(j*2)+1]);
						if(temp < min){
								//print("(" + rnaXpts[i] + ", " + rnaYpts[i]+")");
								//print("Centroid: ("+centroids[j*2]+", " + centroids[(j*2)+1]);
								//print("min = " + min);
								//print("temp = " + temp);
								//print("Current group: " + group[i]);
								//print("Change to group: " + j);
								min = temp;   //replace if find a smaller temp
								group[i] = j;  //register the point to nucleus
						}
				}
				minVal[i] = min;
```

```
	}
	for(i = 0; i < group.length; i++){	//count number of pts for each nucleus
		rois[group[i]] = rois[group[i]] + 1;
		setResult("x", nResults, rnaXpts[i]);
		setResult("y", nResults-1, rnaYpts[i]);
		setResult("group", nResults-1, group[i]);
		setResult("dis", nResults-1, minVal[i]);
	}
	updateResults();
	for(i = 0; i < rois.length; i++){	//make each group of pts into roi
		selectXpts = newArray(rois[i]);
		selectYpts = newArray(rois[i]);
		print("Points belong to nucleus " + i +": \t" + rois[i]);
		j = 0;
		for(k = 0; k < group.length; k++){
			if (group[k] == i){
				selectXpts[j] = rnaXpts[k];
				selectYpts[j] = rnaYpts[k];
				j = j + 1;
			}
		}
		makeSelection("point", selectXpts, selectYpts);
		temp = string +" in Nucleus " + i;
		RoiRename(temp);
		rois[i] = roiManager("index");
	}
	return rois;
}

function CalPI(a, b, rog){
```

```
		pi = Distance(a[0], a[1], b[0], b[1]);
		pi = pi / rog;
		return pi;
	}

	function ImgPreparation(){

		run("Set Scale...", "distance=0 known=0 unit=pixel");
		getDimensions(width, height, channels, slices, frames);
		if(slices > 1)
			run("Z Project...", "projection=[Max Intensity]");
		for(i=1; i<=nSlices;i++){
			setSlice(i);
			resetMinAndMax();
		}
		roiManager("reset");
	}

	function GetCentroid(index, string){
		coord = newArray();
		for(i=0; i<index.length;i++){
			if(index[i] > -1){
				roiManager("select", index[i]);
				List.setMeasurements;
				x = getValue("X");
				y = getValue("Y");
				coord = Array.concat(coord, x);
				coord = Array.concat(coord, y);
				temp = string + " " + i;
				print(temp + ": \t(" +x + ", " +y+")");
```

```
				makePoint(x, y);
				RoiRename(temp);
			}
		}
		return coord;
}

//calculate the radius of gyration
function GetCellROG(index, centroid){
		roiManager("select", index);
		Roi.getContainedPoints(xpoints, ypoints);
		total = 0;
		for(i = 0; i<xpoints.length;i++){
			total = total + pow(Distance(xpoints[i], ypoints[i],centroid[0], centroid[1]),2);
		}
		//print("after sum total: " +total);
		total = sqrt(total/xpoints.length);
		print("Radius of Gyration: \t" + total);
		return total;
}

function GetMaximaPt(slice, index, string){
		print("At slice " + slice);
		prominence = 1;
		notOk = true;
		while(notOk){
			roiManager("select", index);
			setSlice(slice);
			run("Find Maxima...", "prominence="+prominence+" output=[Point Selection]");
```

```
			Dialog.create("Set prominence...");
			//Dialog.addMessage(Set);
			Dialog.addNumber("Current prominence", prominence);
			Dialog.addCheckbox("Accept the current prominence", false);
			Dialog.show();
			prominence = Dialog.getNumber();
			notOk = !Dialog.getCheckbox();
		}
		print("Selected prominence: \t" + prominence);
		getSelectionCoordinates(xpoints, ypoints);
		RoiRename(string);
		print("Points detected: \t" + xpoints.length);
		return roiManager("index");
	}

	function SecondMoment(centroidX, centroidY, xpts, ypts){
		sum = 0;
		for(i = 0; i<xpts.length; i++){
			sum = sum + pow((xpts[i] - centroidX),2) + pow((ypts[i] - centroidY),2);
			//sum = sum + pow(Distance(xpts[i], ypts[i], centriodX, centriodY),2);
		}
		sum = sum / xpts.length;
		return sum;
	}

	function AskForNuclei(){
		nuclei = 1;
		Dialog.create("Number of nuclei...");
		Dialog.addNumber("Number of nuclei in the image", 1);
		Dialog.show();
```

```
		nuclei = Dialog.getNumber();
		return nuclei;
}

function GetNucleus(number){
		roi = newArray();
		run("Wand Tool...", "tolerance=2000 mode=Legacy");
		for(i = 0; i < number; i++){
				boundNotFound = true;
				while(boundNotFound){
						waitForUser(i + "/" + number +" Please select the nucleus boundary.");
						if(selectionType() > -1){
								string = "Nuclei " + i;
								RoiRename(string);
								roi = Array.concat(roi,roiManager("index"));
								boundNotFound = false;
						}
				}
		}
		return roi;
}

function RoiRename(name){
		roiManager("add");
		roiManager("select", roiManager("count")-1);
		roiManager("remove slice info");
		roiManager("rename", name);
}

function Distance(ax, ay, bx, by){
```

```
	return sqrt(pow((ax-bx),2) + pow((ay-by),2));
}
```

Macro script ICW_distanceAnalysis

Fiji macro script used for distance analysis for RNA-protein co-staining images.

```
//initialize
fn=getTitle();
roiManager("reset");
run("Clear Results");
run("Select None");
getDimensions(ImageWidth, ImageHeight, ImageChannels, ImageSlices, ImageFrames);
getVoxelSize(px, py, pz, unit);

//create distance map of channel 3
run("Duplicate...", "title=red duplicate channels=3");
run("Enhance Contrast", "saturated=0.35");
run("Threshold...");
setAutoThreshold("Default dark");
setThreshold(1429, 65535, "raw");
waitForUser("check threshold for red channel");
setOption("BlackBackground", false);
run("Convert to Mask", "method=Default background=Dark");
run("Invert", "stack");
run("Exact Signed Euclidean Distance Transform (3D)");
selectWindow("red");
close();

//create particle roi mask of channel 2 (max projection)
```

```
selectWindow(fn);
run("Duplicate...", "title=green duplicate channels=2");
run("Z Project...", "projection=[Max Intensity]");
setAutoThreshold("Otsu dark");
waitForUser("check threshold for particles");
run("Analyze Particles...", "size=0.1-Infinity exclude add");//particle size
close();


n=roiManager("count");

//find particles in stack and name
selectWindow("green");
setAutoThreshold("Otsu dark");
for (roi=0;roi<n;roi++){
	r1=roiManager("count");
	roiManager("select", roi);
	roiManager("rename", "particle region "+roi+1);
	roiManager("Select", roi);
	Roi.setDefaultGroup(roi+1);
	run("Analyze Particles...", "size=0.10-Infinity add stack");//particle size
	r2=roiManager("count");
	sl=1;
		for (p=r1;p<r2;p++){
			roiManager("select", p);
			roiManager("rename", "part "+roi+1+" "+sl);
			sl+=1;
		}
}
close();
```

```
//remove original mask rois
roiManager("deselect");
roiManager("select", Array.getSequence(n));
roiManager("delete");

n_Total=roiManager("count");
//particles=Array.slice(Array.getSequence(n_Total),n,n_Total);

selectWindow("EDT");
rename(fn+" EDT");

//get data
roiManager("deselect");
roiManager("measure");
Min_Data=Table.getColumn("Min");
Group_Data=Table.getColumn("Group");
selectWindow("Results");
run("Close");
//calibrate data
for (roi=0;roi<n;roi++){
	Min_Data[roi]=Min_Data[roi]*px;
}
//print data
Table.create(fn+" data");
Table.setColumn("Particle", Group_Data);
Table.setColumn("Min distance (um)", Min_Data);
```

DATA AND STATISTICS
