# Supplementary material for: Microtubule-Mediated Regulation of β2AR Translation and Function in Failing Hearts
Source: Circ Res. 2023 Oct 23;133(11):944–58. doi: 10.1161/CIRCRESAHA.123.323174 (PMC10635332; doi:10.1161/CIRCRESAHA.123.323174)
Supplement: Supplementary file 2 [file res-133-944-s002.pdf]

Figure 2E

	Polarisation Index							
	β_1AR				β_2AR			
	0.07452	0.06984	0.03768	0.06306	0.20285	0.16715	0.11643	0.12254
	0.01809	0.03852	0.08742	0.0466	0.092915	0.06886	0.12732	0.09997
	0.08549	0.05503	0.12167	0.08598	0.07489	0.09077	0.11519	0.05716
	0.1508	0.07795	0.07291	0.0466	0.120485	0.08458	0.02958	0.09997
	0.24875	0.19562	0.03894	0.08598	0.3167	0.08313	0.1267	0.05716
	0.10347	0.08474	0.09936	0.06641	0.082365	0.16445	0.08056	0.12984
	0.10319	0.04069	0.04405	0.06663	0.1672	0.14748	0.11298	0.10077
	0.086	0.11246	0.14185	0.0494	0.093275	0.19632	0.11051	0.11256
	0.14444	0.06523		0.0338	0.139	0.10915		0.14452
	0.2005	0.10488			0.1555	0.19535		
Number of values	10	10	8	9	10	10	8	9
Minimum	0.01809	0.03852	0.03768	0.0338	0.07489	0.06886	0.02958	0.05716
Maximum	0.2488	0.1956	0.1419	0.08598	0.3167	0.1963	0.1273	0.1445
Range	0.2307	0.1571	0.1042	0.05218	0.2418	0.1275	0.09774	0.08736
Mean	0.1215	0.08449	0.08048	0.06049	0.1445	0.1307	0.1024	0.1027
Std. Deviation	0.06661	0.04604	0.0393	0.01803	0.07325	0.04889	0.0328	0.02988
Std. Error of Mean	0.02107	0.01456	0.01389	0.00601	0.02316	0.01546	0.0116	0.00996
Shapiro-Wilk test								
W	0.9518	0.8475	0.9196	0.9262	0.8506	0.8896	0.745	0.911
P value	0.6896	0.0542	0.4266	0.4461	0.059	0.1679	0.0072	0.3226
Passed normality test (alpha=0.05)?	Yes	Yes	Yes	Yes	Yes	Yes	No	Yes
P value summary	ns	ns	ns	ns	ns	ns	**	ns

PI	
Wilcoxon matched-pairs signed rank test	
P value	0.000519953
Exact or approximate P value?	Exact
P value summary	***
Significantly different (P < 0.05)?	Yes
One- or two-tailed P value?	Two-tailed
Sum of positive, negative ranks	573.0 , -130.0
Sum of signed ranks (W)	443
Number of pairs	37
Number of ties (ignored)	0
Median of differences	
Median	0.04392
How effective was the pairing?	
rs (Spearman)	0.1703
P value (one tailed)	0.156846145
P value summary	ns
Was the pairing significantly effective?	No

	Dispersion Index							
	β_1AR				β_2AR			
	0.12295	0.08142	0.08711	0.1217	0.22845	0.21635	0.28335	0.2755
	0.13315	0.07249	0.121	0.11021	0.2769	0.1455	0.24345	0.2531
	0.12505	0.10444	0.09606	0.09448	0.19405	0.23165	0.2619	0.22885
	0.09809	0.07855	0.08485	0.11021	0.2083	0.13165	0.2454	0.2531
	0.09748	0.124	0.09173	0.09448	0.23745	0.20195	0.23745	0.22885
	0.15865	0.14465	0.11712	0.17735	0.2142	0.19315	0.21405	0.24945
	0.10667	0.09887	0.12606	0.0938	0.2234	0.17795	0.23645	0.1904
	0.06144	0.12585	0.1131	0.12815	0.19495	0.20425	0.269	0.25035
	0.10959	0.11303		0.10692	0.2354	0.24185		0.1615
	0.19015	0.09847			0.277	0.2268		
Number of values	10	10	8	9	10	10	8	9
Minimum	0.06144	0.07249	0.08485	0.0938	0.1941	0.1317	0.2141	0.1615
Maximum	0.1902	0.1447	0.1261	0.1774	0.277	0.2419	0.2834	0.2755
Range	0.1287	0.07216	0.04121	0.08356	0.08295	0.1102	0.0693	0.114
Mean	0.1203	0.1042	0.1046	0.1153	0.229	0.1971	0.2489	0.2323
Std. Deviation	0.03547	0.02314	0.01644	0.02624	0.02938	0.03629	0.02172	0.03561
Std. Error of Mean	0.01122	0.00732	0.00581	0.00875	0.009292	0.01148	0.00768	0.01187
Shapiro-Wilk test								
W	0.9602	0.9623	0.8907	0.7864	0.8994	0.9298	0.9707	0.8765
P value	0.7883	0.8123	0.2378	0.0142	0.216	0.4458	0.9034	0.1442
Passed normality test (alpha=0.05)?	Yes	Yes	Yes	No	Yes	Yes	Yes	Yes
P value summary	ns	ns	ns	*	ns	ns	ns	ns

DI	
Wilcoxon matched-pairs signed rank test	
P value	1.4552E-11
Exact or approximate P value?	Exact
P value summary	****
Significantly different (P < 0.05)?	Yes
One- or two-tailed P value?	Two-tailed
Sum of positive, negative ranks	703.0 , 0.000
Sum of signed ranks (W)	703
Number of pairs	37
Number of ties (ignored)	0
Median of differences	
Median	0.1225
How effective was the pairing?	
rs (Spearman)	0.2551
P value (one tailed)	0.0638158
P value summary	ns
Was the pairing significantly effective?	No

Figure 3C

	PI control						PI vinblastine					
	β_1AR			β_2AR			β_1AR			β_2AR		
	0.05133	0.13105	0.059168	0.12945	0.3348	0.13755	0.037005	0.233355	0.15901	0.048155	0.0268	0.22945
	0.13957	0.04497	0.093685	0.32015	0.27395	0.2109	0.17907	0.09351	0.1531	0.083095	0.119105	0.13592
	0.07408	0.099005	0.09046	0.124	0.146425	0.12445	0.144845	0.04629	0.16885	0.124	0.1281	0.1896
	0.077275	0.07195	0.23415	0.103395	0.20012	0.25765	0.117025	0.121455	0.06008	0.14795	0.156715	0.128655
		0.135265	0.094315	0.101555	0.06291	0.2609	0.1566	0.106495	0.033705	0.114035	0.124	0.10648
		0.05687	0.13255		0.09723	0.28805		0.12688	0.043095	0.10499	0.28682	0.28115
		0.035445	0.10582		0.18045	0.3375			0.02573		0.078495	0.1637
			0.162355			0.20485						
Number of values	4	7	8	5	7	8	5	6	7	6	7	7
Minimum	0.05133	0.03545	0.05917	0.1016	0.06291	0.1245	0.03701	0.04629	0.02573	0.04816	0.0268	0.1065
Maximum	0.1396	0.1353	0.2342	0.3202	0.3348	0.3375	0.1791	0.2334	0.1689	0.148	0.2868	0.2812
Range	0.08824	0.09982	0.175	0.2186	0.2719	0.2131	0.1421	0.1871	0.1431	0.0998	0.26	0.1747
Mean	0.08556	0.08208	0.1216	0.1557	0.1851	0.2277	0.1269	0.1213	0.09194	0.1037	0.1314	0.1764
Std. Deviation	0.03781	0.04042	0.05489	0.09274	0.09548	0.07299	0.05501	0.06198	0.06497	0.03461	0.08037	0.06182
Std. Error of Mean	0.01891	0.01528	0.01941	0.04148	0.03609	0.0258	0.0246	0.0253	0.02456	0.01413	0.03038	0.02337
Shapiro-Wilk test												
W	0.8725	0.9057	0.878	0.6678	0.9717	0.9576	0.8931	0.8931	0.8036	0.9759	0.9023	0.9419
P value	0.3077	0.3671	0.1801	0.0043	0.9104	0.7871	0.373	0.3346	0.0445	0.9293	0.345	0.6555
Passed normality test (alpha=0.05)?	Yes	Yes	Yes	No	Yes	Yes	Yes	Yes	No	Yes	Yes	Yes
P value summary	ns	ns	ns	**	ns	ns	ns	ns	*	ns	ns	ns

PI	2-WAY ANOVA				
Tukey's multiple comparisons test	Predicted (LS) mean diff.	95.00% CI of diff.	Significant?	Summary	Adjusted P Value
Control:Adrb1 vs. Control:Adrb2	-0.0954	-0.152 to -0.0386	Yes	***	0.0002
Control:Adrb1 vs. 2µM Vinblastine:Ad	-0.012	-0.0703 to 0.0463	No	ns	0.9485
Control:Adrb2 vs. 2µM Vinblastine:Ac	0.056	-9.79e-005 to 0.112	No	ns	0.0506
2µM Vinblastine:Adrb1 vs. 2µM Vinbla	-0.0274	-0.0850 to 0.0302	No	ns	0.5965

Test details	Predicted (LS) mean 1	Predicted (LS) mean 2	Predicted (LS) mean diff.	SE of diff.	N1	N2	q	DF
Control:Adrb1 vs. Control:Adrb2	0.0994	0.195	-0.0954	0.0216	19	20	6.3	73
Control:Adrb1 vs. 2µM Vinblastine:Ad	0.0994	0.111	-0.012	0.0222	19	18	0.8	73
Control:Adrb2 vs. 2µM Vinblastine:Ac	0.195	0.139	0.056	0.0213	20	20	3.7	73
2µM Vinblastine:Adrb1 vs. 2µM Vinbla	0.111	0.139	-0.0274	0.0219	18	20	1.8	73

DI control							DI vinblastine					
β_1AR			β_2AR				β_1AR			β_2AR		
0.1382	0.1419	0.124865	0.12945	0.3348	0.13755		0.148	0.2423	0.053019	0.048155	0.0268	0.22945
0.11843	0.077775	0.20295	0.32015	0.27395	0.2109		0.092	0.142455	0.108545	0.083095	0.119105	0.13592
0.08302	0.106985	0.048124	0.124	0.146425	0.12445		0.128	0.06072	0.168	0.124	0.1281	0.1896
0.100725	0.062375	0.107585	0.103395	0.20012	0.25765		0.219	0.08436	0.114055	0.14795	0.156715	0.128655
	0.101285	0.075915	0.101555	0.06291	0.2609		0.255	0.03247	0.05085	0.114035	0.124	0.10648
	0.053375	0.11595		0.09723	0.28805			0.06672	0.04497	0.10499	0.28682	0.28115
	0.173095	0.11206		0.18045	0.3375				0.03548		0.078495	0.1637
		0.138925			0.20485							
4	7	8	5	7	8	Numbe	5	6	7	6	7	7
0.08302	0.05338	0.04812	0.1016	0.06291	0.1245	Minimu	0.092	0.03247	0.03548	0.04816	0.0268	0.1065
0.3183	0.1731	0.203	0.3202	0.3348	0.3375	Maximu	0.325	0.2423	0.168	0.148	0.2868	0.2812
0.2353	0.1197	0.1548	0.2186	0.2719	0.2131	Range	0.233	0.2098	0.1325	0.0998	0.26	0.1747
0.2103	0.1024	0.1158	0.1557	0.1851	0.2277	Mean	0.211	0.1048	0.08213	0.1037	0.1314	0.1764
0.0711	0.04319	0.04546	0.09274	0.09548	0.07299	Std. De	0.053	0.07664	0.04911	0.03461	0.08037	0.06182
0.01482	0.01632	0.01607	0.04148	0.03609	0.0258	Std. Err	0.011	0.03129	0.01856	0.01413	0.03038	0.02337
						Shapiro-Wilk test						
0.9504	0.9446	0.944	0.6678	0.9717	0.9576	W	0.986	0.8654	0.8613	0.9759	0.9023	0.9419
0.2988	0.68	0.6513	0.0043	0.9104	0.7871	P value	0.98	0.2084	0.1554	0.9293	0.345	0.6555
Yes	Yes	Yes	No	Yes	Yes	Passec	Yes	Yes	Yes	Yes	Yes	Yes
ns	ns	ns	**	ns	ns	P value	ns	ns	ns	ns	ns	ns

DI

Tukey's multiple comparisons test	Predicted (LS) mean diff.	95.00% CI of diff.	Significant'	Summary	Adjusted P Value
Control:Adrb1 vs. Control:Adrb2	-0.122	-0.168 to -0.0754	Yes	****	<0.0001
Control:Adrb1 vs. 2μM Vinblastine:Adrb1	-0.00396	-0.0509 to 0.0430	No	ns	0.9961
Control:Adrb2 vs. 2μM Vinblastine:Adrb2	0.00873	-0.0382 to 0.0556	No	ns	0.9612
2μM Vinblastine:Adrb1 vs. 2μM Vinblastine:Adrb2	-0.109	-0.157 to -0.0615	Yes	****	<0.0001

Test details	Predicted (LS) mean 1	Predicted (LS) mean 2	Predicted (I	SE of diff.	N1	N2	q	DF
Control:Adrb1 vs. Control:Adrb2	0.11	0.231	-0.122	0.0176	19	19	9.79	70
Control:Adrb1 vs. 2μM Vinblastine:Adrb1	0.11	0.114	-0.00396	0.0178	19	18	0.314	70
Control:Adrb2 vs. 2μM Vinblastine:Adrb2	0.231	0.223	0.00873	0.0178	19	18	0.692	70
2μM Vinblastine:Adrb1 vs. 2μM Vinblastine:Adrb2	0.114	0.223	-0.109	0.0181	18	18	8.53	70

Figure 4B

	β1AR							
	PNR				Cytosol			
	100	93.137	100	95.313	16.3934	0	33.3333	78.57
	100	93.443	96.72	100	20	47.3684	46.1539	25
	93.056	100	100	99.083	100	40	28.5714	36.36
	100	96	98.55	100	14.2857	15.3846	9.09091	11.11
	100				100			
Number of values	5	4	4	4	5	4	4	4
Minimum	93.06	93.14	96.72	95.31	14.29	0	9.091	11.11
Maximum	100	100	100	100	100	47.37	46.15	78.57
Range	6.944	6.863	3.279	4.687	85.71	47.37	37.06	67.46
Mean	98.61	95.65	98.82	98.6	50.14	25.69	29.29	37.76
Std. Deviation	3.105	3.174	1.556	2.233	45.57	21.92	15.38	29.1
Std. Error of Mean	1.389	1.587	0.778	1.116	20.38	10.96	7.688	14.55
Shapiro-Wilk test								
W	0.5522	0.8746	0.857	0.761	0.7167	0.9322	0.9726	0.917
P value	0.0001	0.3161	0.249	0.0486	0.0142	0.6071	0.8573	0.518
Passed normality test (alpha=0.05)?	No	Yes	Yes	No	No	Yes	Yes	Yes
P value summary	***	ns	ns	*	*	ns	ns	ns

	β2AR							
	PNR				Cytosol			
	67.5676	98.66	94.4444	93.3333	92.5926	96.5766	94.8276	100
	61.3333	64.55	89.2857	100	88.5	86.4583	97.9167	90.6883
	93.3333	62.5	100	77.2466	99.0826	97.5535	93.5484	68.5824
		66.67	72.973	100		75.3846	81.579	92.7645
			73.0769	82.6855		87.3016	92.9293	78.5916
				56.25				94.0299
Number of values	3	4	5	6	3	5	5	6
Minimum	61.33	62.5	72.97	56.25	88.5	75.38	81.58	68.58
Maximum	93.33	98.66	100	100	99.08	97.55	97.92	100
Range	32	36.16	27.03	43.75	10.58	22.17	16.34	31.42
Mean	74.08	73.09	85.96	84.92	93.39	88.65	92.16	87.44
Std. Deviation	16.96	17.13	12.4	16.8	5.336	9.01	6.22	11.61
Std. Error of Mean	9.794	8.565	5.544	6.859	3.081	4.029	2.781	4.74
Shapiro-Wilk test								
W	0.8895	0.722	0.8695	0.8907	0.9832	0.9086	0.8277	0.9065
P value	0.3529	0.02	0.2643	0.3219	0.7516	0.4595	0.1337	0.4137
Passed normality test (alpha=0.05)?	Yes	No	Yes	Yes	Yes	Yes	Yes	Yes
P value summary	ns	*	ns	ns	ns	ns	ns	ns

	Common test (SE)	Common test (p)	Group-level clustering (ICC)	Group-level (SE)	Superior fit (p)
β1AR	7.358	<0.0001	0%	7.138	N(1)
β2AR	4.103	0.0544	0%	3.993	N(1)

β1AR		β2AR	
Wilcoxon matched-pairs signed rank test		Wilcoxon matched-pairs signed rank test	
P value	6.10352E-05	P value	0.038490295
Exact or approximate P value?	Exact	Exact or approximate P value?	Exact
P value summary	****	P value summary	*
Significantly different (P < 0.05)?	Yes	Significantly different (P < 0.05)?	Yes
One- or two-tailed P value?	Two-tailed	One- or two-tailed P value?	Two-tailed
Sum of positive, negative ranks	1.000 , -135.0	Sum of positive, negative ranks	133.0 , -38.00
Sum of signed ranks (W)	-134	Sum of signed ranks (W)	95
Number of pairs	17	Number of pairs	18
Number of ties (ignored)	1	Number of ties (ignored)	0
Median of differences		Median of differences	
Median	-71.43	Median	7.636
How effective was the pairing?		How effective was the pairing?	
rs (Spearman)	-0.1859	rs (Spearman)	0.3104
P value (one tailed)	0.2357	P value (one tailed)	0.105
P value summary	ns	P value summary	ns
Was the pairing significantly effective?	No	Was the pairing significantly effective?	No

Figure 4D

	beta1-tubulin colocalisation vs beta2-tubulin colocalisation					
	β1-tubulin			β2-tubulin		
	0.563	0.742	0.772	0.781	0.734	0.671
	0.546	0.531	0.51	0.623	0.639	0.531
	0.51	0.646	0.713	0.83	0.678	0.642
	0.544	0.537	0.503	0.623	0.634	0.632
	0.527	0.682	0.449	0.506	0.828	0.643
	0.659	0.736	0.532	0.656	0.669	0.725
	0.53	0.589	0.387	0.693	0.616	0.584
	0.566	0.551	0.427	0.703	0.691	0.701
	0.699	0.828	0.318	0.718	0.637	0.64
	0.567	0.634	0.59	0.6	0.767	0.838
	0.572	0.64	0.519	0.626	0.504	0.701
	0.641	0.681	0.5	0.7	0.627	0.644
	0.594	0.532			0.786	0.728
	0.611	0.559			0.716	0.795
	0.416	0.536			0.73	0.639
					0.69	0.665
					0.781	0.636
					0.676	
					0.601	
					0.694	
					0.786	
	15	15	12	12	21	17
Number of values						
	0.416	0.531	0.318	0.506	0.504	0.531
Minimum	0.699	0.828	0.772	0.83	0.828	0.838
Maximum	0.283	0.297	0.454	0.324	0.324	0.307
Range						
	0.5697	0.6283	0.5183	0.6716	0.6897	0.6715
Mean	0.06759	0.09199	0.1275	0.08584	0.07655	0.07311
Std. Deviation	0.01745	0.02375	0.0368	0.02478	0.0167	0.01773
Std. Error of Mean						
Shapiro-Wilk test	0.9608	0.9025	0.942	0.9708	0.9716	0.9352
W	0.7062	0.1038	0.525	0.9188	0.7674	0.2653
P value	Yes	Yes	Yes	Yes	Yes	Yes
Passed normality test (alpha=0.05)?	ns	ns	ns	ns	ns	ns
P value summary						

Table Analyzed	nested	
Column A	β1-tubulin	
vs.	vs.	
Column B	β2-tubulin	
Nested t test		
P value	0.02881	
P value summary	*	
Significantly different (P < 0.05)?	Yes	
One- or two-tailed P value?	Two-tailed	
t, df	t=3.341, df=4	
F, DFn, Dfd	11.16, 1, 4	
How big is the difference?		
Mean of column A	0.5735	
Mean of column B	0.6781	
Difference between means (A - B) ± SEM	-0.1046 ± 0.03129	
95% confidence interval	-0.1914 to -0.01767	
Random effects	SD	Variance
Variation within subcolumns	0.08666	0.00751
Variation among subcolumn means	0.03107	0.00097
Do the subcolumns differ (within each column)?		
Chi-square, df	2.687, 1	
P value	0.10118	
P value summary	ns	
Is there significant difference between subcolumns (P < 0.05)?	No	
Data analyzed		
Number of treatments (columns)	2	
Number of subjects (subcolumns)	6	
Total number of values	92	

Figure 4F

	beta1 vs beta2 FRET response (normalised to saturator) in control vs vinblastine											
	beta1 control						beta2 control					
	55.33924	52.55667	42.20012	49.48137	55.3797	45.6563	29.1637	31.21476	33.7636	49.4198	32.97773	44.31298
	59.63844	66.13898	55.53276	48.79517	65.0637		53.0615	40.13171	40.0952	22.7855	36.3713	27.38813
	39.09486	57.86542	65.91323	51.68145	47.0711			37.38921	46.159	24.9185		
		37.95916		59.50104								
	3	4	3	4	3	1	2	3	3	3	2	2
Number of values												
	39.09	37.96	42.2	48.8	47.07	45.66	29.16	31.21	33.76	22.79	32.98	27.39
Minimum	59.64	66.14	65.91	59.5	65.06	45.66	53.06	40.13	46.16	49.42	36.37	44.31
Maximum	20.54	28.18	23.71	10.71	17.99	0	23.9	8.917	12.4	26.63	3.394	16.92
Range												
	51.36	53.63	54.55	52.36	55.84	45.66	41.11	36.25	40.01	32.37	34.67	35.85
Mean	10.84	11.85	11.89	4.914	9.005	0	16.9	4.567	6.198	14.8	2.4	11.97
Std. Deviation	6.256	5.924	6.863	2.457	5.199	0	11.95	2.637	3.579	8.545	1.697	8.462
Std. Error of Mean												
Shapiro-Wilk test	0.8987	0.9737	0.9949	0.8234	0.9981	N too sma	N too sma	0.9529	0.9998	0.8096	N too small	N too small
W	0.3814	0.8642	0.863	0.1513	0.9158			0.5824	0.9762	0.1377		
P value	Yes	Yes	Yes	Yes	Yes			Yes	Yes	Yes		
Passed normality test (alpha=0.05)?	ns	ns	ns	ns	ns			ns	ns	ns		

	beta1 vinblastine						beta2 vinblastine					
	45.3948	51.1629	50.2125	57.37934	54.8644	58.4234	16.99005	15.87322	17.5254	13.19521	9.177015	18.01492
	55.1205	58.8806	62.38902	52.49609	63.6423	61.7818	14.89129	12.10221	3.64024	18.40792	7.009616	10.85957
	61.2513	52.0461	64.93151		65.1715	61.0941			4.25145	2.544454		
			51.37116							7.296422		
	3	3	4	2	3	3	2	2	3	4	2	2
Number of values												
	45.39	51.16	50.21	52.5	54.86	58.42	14.89	12.1	3.64	2.544	7.01	10.86
Minimum	61.25	58.88	64.93	57.38	65.17	61.78	16.99	15.87	17.53	18.41	9.177	18.01
Maximum	15.86	7.718	14.72	4.883	10.31	3.358	2.099	3.771	13.89	15.86	2.167	7.155
Range												
	53.92	54.03	57.23	54.94	61.23	60.43	15.94	13.99	8.472	10.36	8.093	14.44
Mean	7.996	4.224	7.517	3.453	5.562	1.774	1.484	2.667	7.846	6.911	1.533	5.06
Std. Deviation	4.616	2.439	3.758	2.442	3.211	1.024	1.049	1.886	4.53	3.455	1.084	3.578
Std. Error of Mean												
Shapiro-Wilk test	0.9832	0.8346	0.8423	N too small	0.8585	0.8959	N too small	N too small	0.7829	0.9856	N too small	N too small
W	0.7514	0.2	0.2021		0.2634	0.3725			0.0744	0.9342		
P value	Yes	Yes	Yes		Yes	Yes			Yes	Yes		
Passed normality test (alpha=0.05)?	ns	ns	ns		ns	ns			ns	ns		

P value summary								
	Predicted (LS) mean diff.	95.00% CI of diff.	Significant?	Summary	Adjusted P Value			
Tukey's multiple comparisons test								
	16.44	9.610 to 23.27	Yes	****	1.64E-07			
Control :ß1AR vs. Control :ß2AR	-4.041	-10.55 to 2.469	No	ns	0.364761			
Control :ß1AR vs. 2uM vinblastine :ß1AR	25.16	18.03 to 32.29	Yes	****	2.02E-11			
Control :ß2AR vs. 2uM vinblastine :ß2AR	45.64	38.81 to 52.47	Yes	****	1.89E-11			
2uM vinblastine :ß1AR vs. 2uM vinblastine :ß2	45.64	38.81 to 52.47	Yes	****	1.89E-11			
	Predicted (LS) mean 1	Predicted (LS) mean 2	Predicted (LS) mean diff.	SE of diff.	N1	N2	q	DF
Test details								
	53.05	36.61	16.44	2.586	18	15	8.989	62
Control :ß1AR vs. Control :ß2AR	53.05	57.09	-4.041	2.466	18	18	2.318	62
Control :ß1AR vs. 2uM vinblastine :ß1AR	36.61	11.45	25.16	2.701	15	15	13.17	62
Control :ß2AR vs. 2uM vinblastine :ß2AR	57.09	11.45	45.64	2.586	18	15	24.96	62
2uM vinblastine :ß1AR vs. 2uM vinblastine :ß2	57.09	11.45	45.64	2.586	18	15	24.96	62

Figure 4G

	beta1 vs beta2 protein (intensity, on membrane)												
	β1AR							β2AR					
	Control			2µM Vin				Control			2µM Vin		
	4632.841	2853.301	4205.808	3543.4	2326.94	3894.07	3120.5	6718.649	5929.872	8443.849	4346.78	7757.626	5110.559
	4492.546	5917.78	4080.729	3072.37	2405.87	3782.23	2971.9	10014.41	7751.661	8004.025	4680.74	5119.359	5467.607
	4296.691	5746.169	5030.709	2009.63	2372.64	3892.78	2865.3	6974.581	9681.486	6477.168	3923.94	6931.135	5226.732
	3848.414	5806.863	4541.49	2276.31	2325.62	3892.75		8604.619			4793.83		5066.631
	3347.599	3920.229	3914.301	2239.37	3755.72	3818.24							5988.886
	5	5	5	5	5	5	3	4	3	3	4	3	5
Number of values													
	3348	2853	3914	2010	2326	3782	2865	6719	5930	6477	3924	5119	5067
Minimum	4633	5918	5031	3543	3756	3894	3120	10014	9681	8444	4794	7758	5989
Maximum	1285	3064	1116	1534	1430	111.8	255.2	3296	3752	1967	869.9	2638	922.3
Range													
	4124	4849	4355	2628	2637	3856	2986	8078	7788	7642	4436	6603	5372
Mean	525.2	1388	442.5	650.5	626.1	52.49	128.2	1538	1876	1032	390.8	1349	378.3
Std. Deviation	234.9	620.9	197.9	290.9	280	23.47	74	768.8	1083	595.9	195.4	779.1	169.2
Std. Error of Mean													
Shapiro-Wilk test	0.924	0.8055	0.9297	0.8801	0.5995	0.7735	0.9911	0.9069	0.9997	0.9076	0.9319	0.9556	0.854
W	0.5561	0.0898	0.5941	0.3096	0.0006	0.0484	0.8191	0.4663	0.9682	0.41	0.6058	0.5944	0.2074
P value	Yes	Yes	Yes	Yes	No	No	Yes	Yes	Yes	Yes	Yes	Yes	Yes
Passed normality test (alpha=0.05)?	ns	ns	ns	ns	***	*	ns	ns	ns	ns	ns	ns	ns
P value summary													

β1AR		β2AR	
Mann Whitney test		Unpaired t test	
P value	0.000007	P value	0.00010739
Exact or approximate P value?	Exact	P value summary	***
P value summary	****	Significantly different (P < 0.05)?	Yes
Significantly different (P < 0.05)?	Yes	One- or two-tailed P value?	Two-tailed
One- or two-tailed P value?	Two-tailed	t, df	t=4.806, df=20
Sum of ranks in column A,B	369 , 192		
Mann-Whitney U	21	How big is the difference?	
		Mean of column A	7860
Difference between medians		Mean of column B	5368
Median of column A	4297, n=15	Difference between means (B - A) ± SEM	-2492 ± 518.5
Median of column B	3022, n=18	95% confidence interval	-3574 to -141
Difference: Actual	-1275	R squared (eta squared)	0.536
Difference: Hodges-Lehmann	-1482		
		F test to compare variances	
		F, DFn, Dfd	1.596, 9, 11
		P value	0.45842439
		P value summary	ns
		Significantly different (P < 0.05)?	No
		Data analyzed	
		Sample size, column A	10
		Sample size, column B	12

Figure 5B

smFISH clustering analysis PI

	Control beta1					Control beta2					MI beta1			MI beta2		
	0.074515	0.10347	0.19562	0.072905	0.046595	0.20285	0.082365	0.083125	0.02958	0.09997	0.13555	0.19816	0.15305	0.22255	0.22195	0.26455
	0.018093	0.10319	0.084735	0.038935	0.085975	0.092915	0.1672	0.16445	0.1267	0.05716	0.22905	0.090765	0.19485	0.1719	0.151495	0.2045
	0.08549	0.085995	0.040685	0.099355	0.066405	0.07489	0.093275	0.14748	0.080555	0.12984	0.1102	0.047235	0.1673	0.2192	0.10304	0.180295
	0.1508	0.144435	0.112455	0.044045	0.066625	0.120485	0.139	0.196315	0.11298	0.10077	0.13565	0.148155	0.019715	0.16545	0.208145	0.141945
	0.24875	0.2005	0.06523	0.14185	0.049395	0.3167	0.1555	0.10915	0.110505	0.112555	0.1721	0.079485	0.111	0.14305	0.2011	0.2079
		0.069835	0.104875	0.06306	0.0338		0.16715	0.19535	0.12254	0.144515	0.1789	0.17395	0.11328	0.29355	0.2604	0.2775
		0.038515	0.03768	0.046595			0.06886	0.116425	0.09997		0.2056	0.08537	0.13945	0.36745	0.15912	0.198
		0.05503	0.087415	0.085975			0.09077	0.12732	0.05716		0.1687	0.05672	0.13225	0.2306	0.26105	0.2297
		0.07795	0.12167				0.084575	0.115185			0.12625	0.12815	0.13228	0.2724	0.22135	0.1537
											0.1605	0.1419			0.36935	
Number of values	5	9	9	8	6	5	9	9	8	6	10	10	9	9	10	9
Minimum	0.01809	0.03852	0.03768	0.03894	0.0338	0.07489	0.06886	0.08313	0.02958	0.05716	0.1102	0.04724	0.01972	0.1431	0.103	0.1419
Maximum	0.2488	0.2005	0.1956	0.1419	0.08598	0.3167	0.1672	0.1963	0.1267	0.1445	0.2291	0.1982	0.1949	0.3675	0.3694	0.2775
Range	0.2307	0.162	0.1579	0.1029	0.05218	0.2418	0.09834	0.1132	0.09712	0.08736	0.1189	0.1509	0.1751	0.2244	0.2663	0.1356
Mean	0.1155	0.09766	0.09449	0.07409	0.05813	0.1616	0.1165	0.1394	0.0925	0.1075	0.1623	0.115	0.1292	0.2318	0.2157	0.2065
Std. Deviation	0.08813	0.0493	0.04811	0.03462	0.01853	0.0996	0.04003	0.03938	0.03427	0.0301	0.0368	0.05066	0.04877	0.07069	0.07304	0.04565
Std. Error of Mean	0.03941	0.01643	0.01604	0.01224	0.007566	0.04454	0.01334	0.01313	0.01212	0.01229	0.01164	0.01602	0.01626	0.02356	0.0231	0.01522
Shapiro-Wilk test																
W	0.949	0.9155	0.9197	0.9068	0.9632	0.8835	0.8442	0.9282	0.8936	0.9496	0.964	0.9522	0.8872	0.9477	0.9503	0.9596
P value	0.7302	0.3561	0.3896	0.332	0.8441	0.3257	0.0643	0.464	0.2528	0.7367	0.8306	0.6941	0.1865	0.6646	0.6726	0.7943
Passed normality test (alpha=0.05)?	Yes	Yes	Yes	Yes	Yes	Yes	Yes	Yes	Yes	Yes	Yes	Yes	Yes	Yes	Yes	Yes
P value summary	ns	ns	ns	ns	ns	ns	ns	ns	ns	ns	ns	ns	ns	ns	ns	ns

smFISH clustering analysis PI

Sidak's multiple comparisons test	Mean Diff.	95.00% CI	Significant	Summary	Adjusted P Value
Control beta1 vs. MI beta1	-0.04791	-0.09219 t	Yes	*	0.0323
MI beta1 vs. MI beta2	-0.08227	-0.1301 to	Yes	**	0.0012
Control beta2 vs. MI beta2	-0.09611	-0.1407 to	Yes	***	0.0002

smFISH clustering analysis DI

	Control beta1					Control beta2					MI beta1			MI beta2		
	0.122945	0.15865	0.124	0.084845	0.110205	0.22845	0.2142	0.20195	0.2454	0.2531	0.1309	0.2402	0.20655	0.28425	0.322	0.3004
	0.13315	0.10667	0.14465	0.091725	0.094475	0.2769	0.2234	0.19315	0.23745	0.22885	0.07027	0.12935	0.17445	0.283	0.3373	0.348
	0.125045	0.061435	0.098865	0.117115	0.17735	0.19405	0.19495	0.17795	0.21405	0.24945	0.09714	0.15285	0.1774	0.28785	0.30985	0.2286
	0.098085	0.109585	0.12585	0.126055	0.093795	0.2083	0.2354	0.20425	0.23645	0.1904	0.154	0.21475	0.07771	0.24945	0.30695	0.2956
	0.097475	0.19015	0.113025	0.113095	0.12815	0.23745	0.277	0.24185	0.269	0.25035	0.1986	0.19895	0.17265	0.27045	0.3323	0.28545
		0.081415	0.09847	0.1217	0.10692		0.21635	0.2268	0.2755	0.1615	0.1977	0.1765	0.1793	0.26225	0.29965	0.36705
		0.07249	0.08711	0.110205			0.1455	0.28335	0.2531		0.20555	0.10445	0.14915	0.31005	0.27965	0.27855
		0.10444	0.121	0.094475			0.23165	0.24345	0.22885		0.2296	0.1638	0.1382	0.2885	0.3091	0.2933
		0.078545	0.09606				0.13165	0.2619			0.1535	0.1584	0.2244	0.27875	0.22375	0.3158
											0.17795	0.1538		0.28095	0.2851	
Number of values	5	9	9	8	6	5	9	9	8	6	10	10	9	10	10	9
Minimum	0.09748	0.06144	0.08711	0.08485	0.0938	0.1941	0.1317	0.178	0.2141	0.1615	0.07027	0.1045	0.07771	0.2495	0.2238	0.2286
Maximum	0.1332	0.1902	0.1447	0.1261	0.1774	0.2769	0.277	0.2834	0.2755	0.2531	0.2296	0.2402	0.2244	0.3101	0.3373	0.3671
Range	0.03568	0.1287	0.05754	0.04121	0.08356	0.08285	0.1454	0.1054	0.06145	0.0916	0.1593	0.1358	0.1467	0.0606	0.1136	0.1385
Mean	0.1153	0.107	0.1121	0.1074	0.1185	0.229	0.2078	0.2261	0.245	0.2223	0.1615	0.1693	0.1666	0.2796	0.3006	0.3014
Std. Deviation	0.01648	0.04232	0.01842	0.01516	0.03145	0.03169	0.04517	0.03459	0.02043	0.03803	0.05062	0.04012	0.04232	0.01634	0.03265	0.0401
Std. Error of Mean	0.007369	0.01411	0.006138	0.00536	0.01284	0.01417	0.01506	0.01153	0.007222	0.01552	0.01601	0.01269	0.01411	0.005168	0.01032	0.01337
Shapiro-Wilk test																
W	0.8407	0.8797	0.9474	0.9235	0.8142	0.9603	0.9249	0.9655	0.9706	0.8317	0.9496	0.9754	0.9223	0.954	0.8768	0.9527
P value	0.167	0.1559	0.6611	0.4589	0.0786	0.8098	0.4339	0.8533	0.9029	0.1111	0.6642	0.9356	0.4112	0.7162	0.1197	0.7198
Passed normality test (alpha=0.05)?	Yes	Yes	Yes	Yes	Yes	Yes	Yes	Yes	Yes	Yes	Yes	Yes	Yes	Yes	Yes	Yes
P value summary	ns	ns	ns	ns	ns	ns	ns	ns	ns	ns	ns	ns	ns	ns	ns	ns

smFISH clustering analysis DI

Sidak's multiple comparisons test	Mean Diff	95.00% CI	Significar	Summary	Adjusted P Value
Control beta1 vs. MI beta1	-0.05447	-0.07602 t	Yes	****	1.16E-08
Control beta2 vs. MI beta2	-0.06809	-0.08964 t	Yes	****	2.86E-12
MI beta1 vs. MI beta2	-0.1278	-0.1506 to	Yes	****	<0.000000000000001

Test details	Mean 1	Mean 2	Mean Diff	SE of diff	N1	N2	t	DF
Control beta1 vs. MI beta1	0.1113	0.1658	-0.05447	0.008531	37	29	6.384	128
Control beta2 vs. MI beta2	0.2255	0.2936	-0.06809	0.008531	37	29	7.981	128
MI beta1 vs. MI beta2	0.1658	0.2936	-0.1278	0.009034	29	29	14.15	128

Figure 6B

Polarization index

	Control beta1			Imp beta1			control beta2			Imp beta2		
	0.07452	0.08474	0.08598	0.1153	0.08266	0.18929	0.20285	0.16445	0.05716	0.45015	0.11798	0.12645
	0.01809	0.04069	0.0466	0.123	0.1448	0.4596	0.09292	0.14748	0.09997	0.09581	0.1501	0.1464
	0.08549	0.11246	0.08598	0.19645	0.426	0.175	0.07489	0.19632	0.05716	0.373	0.4134	0.2841
	0.1508	0.06523	0.06641	0.03486	0.1214	0.07255	0.12049	0.10915	0.12984	0.10633	0.20355	0.19004
	0.24875	0.10488	0.06663	0.20235	0.12745	0.2652	0.3167	0.19535	0.10077	0.29105	0.2361	0.20845
	0.10347	0.03768	0.0494	0.23635	0.06052	0.08208	0.08237	0.11643	0.11256	0.344	0.169	0.10933
	0.10319	0.08742	0.0338	0.07197	0.06948		0.1672	0.12732	0.14452	0.08477	0.303	
	0.086	0.12167	0.17025	0.11495			0.09328	0.11519	0.30455	0.03109		
	0.14444	0.07291	0.16955	0.10336			0.139	0.02958	0.15645	0.16457		
	0.2005	0.03894	0.1727	0.3319			0.1555	0.1267	0.35295	0.5811		
	0.06984	0.09936	0.1205	0.05155			0.16715	0.08056	0.30235	0.2794		
	0.03852	0.04405					0.06886	0.11298				
	0.05503	0.14185					0.09077	0.11051				
	0.07795	0.06306					0.08458	0.12254				
	0.19562	0.0466					0.08313	0.09997				
Number of values	15	15	11	11	7	6	15	15	11	11	7	6
Minimum	0.01809	0.03768	0.0338	0.03486	0.06052	0.07255	0.06886	0.02958	0.05716	0.03109	0.118	0.1093
Maximum	0.2488	0.1419	0.1727	0.3319	0.426	0.4596	0.3167	0.1963	0.353	0.5811	0.4134	0.2841
Range	0.2307	0.1042	0.1389	0.297	0.3655	0.3871	0.2478	0.1667	0.2958	0.55	0.2954	0.1748
Mean	0.1101	0.07743	0.09707	0.1438	0.1475	0.2073	0.1293	0.1236	0.1653	0.2547	0.2276	0.1775
Std. Deviation	0.06512	0.03335	0.05274	0.08911	0.1268	0.143	0.06618	0.04189	0.1048	0.174	0.1019	0.06434
Std. Error of Mean	0.01681	0.00861	0.0159	0.02687	0.04794	0.05837	0.01709	0.01082	0.03159	0.05248	0.03852	0.02627
Shapiro-Wilk test												
W	0.931	0.9318	0.8642	0.9248	0.6837	0.8895	0.8034	0.9271	0.8398	0.9438	0.9234	0.9367
P value	0.282	0.2899	0.0652	0.361	0.0025	0.3156	0.0041	0.2468	0.0314	0.566	0.4965	0.6326
Passed normality test (alpha=0.05)?	Yes	Yes	Yes	Yes	No	Yes	No	Yes	No	Yes	Yes	Yes
P value summary	ns	ns	ns	ns	**	ns	**	ns	*	ns	ns	ns

Disperson index

	Control beta1			Control beta2			Control beta2			Imp beta2		
	0.122945	0.14465	0.09448	0.23215	0.1732	0.07595	0.22845	0.19315	0.22885	0.06459	0.377	0.4355
	0.13315	0.09887	0.11021	0.15415	0.057355	0.121075	0.2769	0.17795	0.2531	0.3168	0.3667	0.3579
	0.125045	0.12585	0.09448	0.19195	0.5621	0.07431	0.19405	0.20425	0.22885	0.26205	0.8785	0.3352
	0.098085	0.11303	0.17735	0.12057	0.012025	0.05421	0.2083	0.24185	0.24945	0.154025	0.2746	0.3533
	0.097475	0.09847	0.0938	0.2625	0.04602	0.173085	0.23745	0.2268	0.1904	0.3224	0.2407	0.42555
	0.15865	0.08711	0.12815	0.2619	0.1518	0.11544	0.2142	0.28335	0.25035	0.305	0.8514	0.3683
	0.10667	0.121	0.10692	0.16425	0.07647		0.2234	0.24345	0.1615	0.28515		0.36455
	0.061435	0.09606	0.11812	0.1423			0.19495	0.2619	0.498	0.036961		
	0.109585	0.08485	0.14915	0.1605			0.2354	0.2454	0.56885	0.13765		
	0.19015	0.09173	0.09342	0.241			0.277	0.23745	0.47545	0.1094		
	0.081415	0.11712	0.13075	0.15835			0.21635	0.21405	0.27125	0.35185		
	0.07249	0.12606					0.1455	0.23645				
	0.10444	0.1131					0.23165	0.269				
	0.078545	0.1217					0.13165	0.2755				
	0.124	0.11021					0.20195	0.2531				
Number of values	15	15	11	11	7	6	15	15	11	11	6	7
Minimum	0.06144	0.08485	0.09342	0.1206	0.01203	0.05421	0.1317	0.178	0.1615	0.03696	0.2407	0.3352
Maximum	0.1902	0.1447	0.1774	0.2625	0.5621	0.1731	0.277	0.2834	0.5689	0.3519	0.8785	0.4355
Range	0.1287	0.05981	0.08394	0.1419	0.5501	0.1189	0.1454	0.1054	0.4074	0.3149	0.6378	0.1003
Mean	0.1109	0.11	0.1179	0.19	0.1541	0.1023	0.2145	0.2376	0.3069	0.2133	0.4981	0.3772
Std. Deviation	0.03361	0.01686	0.02691	0.05072	0.1889	0.04319	0.0396	0.03021	0.1382	0.1145	0.289	0.03804
Std. Error of Mean	0.008679	0.00435	0.00811	0.01529	0.0714	0.01763	0.01023	0.0078	0.04168	0.03451	0.118	0.01438
Shapiro-Wilk test												
W	0.9503	0.9594	0.8657	0.8926	0.7274	0.9283	0.9306	0.9686	0.8047	0.8919	0.7858	0.8489
P value	0.5287	0.6827	0.0682	0.1499	0.0073	0.5671	0.2781	0.8371	0.0109	0.1467	0.0437	0.1202
Passed normality test (alpha=0.05)?	Yes	Yes	Yes	Yes	No	Yes	Yes	Yes	No	Yes	No	Yes
P value summary	ns	ns	ns	ns	**	ns	ns	ns	*	ns	*	ns

Sidak's multiple comparisons test	Predicted	95.00% CI of diff.	Significant?	Summary	Adjusted P Value			
Control:β_1AR vs. 300μM Imipramine:β_1AR	-0.0661	-0.1280 to -0.004166	Yes	*	0.03			
Control:β_2AR vs. 300μM Imipramine:β_2AR	-0.0906	-0.1525 to -0.02866	Yes	***	0.0009			
300μM Imipramine:β_1AR vs. 300μM Imipramine:β_2AR	-0.0667	-0.1363 to 0.002830	No	ns	0.0671			
Test details	Predicted	Predicted (LS) mean 2	Predicted (LS) mean diff.	SE of diff.	N1	N2	t	DF
Control:β_1AR vs. 300μM Imipramine:β_1AR	0.09467	0.1608	-0.06608	0.02316	41	24	2.853	126
Control:β_2AR vs. 300μM Imipramine:β_2AR	0.1369	0.2275	-0.09058	0.02316	41	24	3.91	126
300μM Imipramine:β_1AR vs. 300μM Imipramine:β_2AR	0.1608	0.2275	-0.06671	0.02602	24	24	2.564	126

Sidak's multiple comparisons test	Predicted	95.00% CI of diff.	Significant?	Summary	Adjusted P Value			
Control:beta1 vs. 300μM Imipramine:beta1	-0.0452	-0.1196 to 0.02934	No	ns	0.4951324			
Control:b2AR vs. 300μM Imipramine:b2AR	-0.0846	-0.1591 to -0.01007	Yes	*	0.017441			
300μM Imipramine:beta1 vs. 300μM Imipramine:b2AR	-0.1747	-0.2584 to -0.09101	Yes	****	8.4E-07			
Test details	Predicted	Predicted (LS) mean 2	Predicted (LS) mean diff.	SE of diff.	N1	N2	t	DF
Control:beta1 vs. 300μM Imipramine:beta1	0.1125	0.1576	-0.04516	0.02787	41	24	1.62	126
Control:b2AR vs. 300μM Imipramine:b2AR	0.2477	0.3323	-0.08456	0.02787	41	24	3.034	126
300μM Imipramine:beta1 vs. 300μM Imipramine:b2AR	0.1576	0.3323	-0.1747	0.0313	24	24	5.581	126

Figure 6C

	β1AR (ISO)								
	Control			300uM imipramine			MI		
	7.1587	2.82253	2.258339	3.01885	6.3181	9.0941	3.69162	5.83983	6.77557
	8.7772	2.59435	2.343762	4.98919	7.8338	4.6964	1.84192	3.59882	5.37155
	6.1682	3.06983	3.690428	3.9081	7.5093	9.7685	3.98786	3.12091	4.51817
	4.7668	3.11761	3.07103		5.9659	4.5836	4.23997	8.91757	8.07848
	4.5351		2.126629		2.3573	4.7426	2.15363	3.90726	4.58
	10.0107		3.703286		3.7788		3.8732	6.04437	8.28279
					6.5247		2.66526	4.49666	3.70852
					7.5137		5.72067	3.9926	0.80547
					13.4164		2.14519	5.51828	0.76793
					2.5746		5.31704	6.36363	1.17861
					6.6632		3.16346	6.78228	1.66093
					2.7804		1.61799	6.35248	1.12219
					6.905		6.54403	3.89233	1.36494
					6.0899		3.06682	5.51054	1.3441
							5.40051	4.85146	2.36285
							4.73228	8.97191	1.77129
							1.29751	4.57352	2.95993
							0.62176		
							3.34756		
							0.89072		
							1.15887		
							1.64858		
							2.37982		
							1.07695		
							4.01779		
							2.57122		
							2.52177		
Number of values	6	4	6	3	14	5	27	17	17
Minimum	4.535	2.594	2.127	3.019	2.357	4.584	0.6218	3.121	0.7679
Maximum	10.01	3.118	3.703	4.989	13.42	9.769	6.544	8.972	8.283
Range	5.476	0.5233	1.577	1.97	11.06	5.185	5.922	5.851	7.515
Mean	6.903	2.901	2.866	3.972	6.159	6.577	3.026	5.455	3.333
Std. Deviation	2.188	0.242	0.7227	0.9867	2.836	2.617	1.6	1.699	2.534
Std. Error of Mean	0.8934	0.121	0.295	0.5697	0.7578	1.17	0.3079	0.4121	0.6147
Shapiro-Wilk test									
W	0.9358	0.9145	0.8387	0.9969	0.8727	0.7416	0.9623	0.9198	0.8629
P value	0.6257	0.5067	0.1272	0.8928	0.0457	0.0248	0.4165	0.1467	0.017
Passed normality test (alpha=0.05)?	Yes	Yes	Yes	Yes	No	No	Yes	Yes	No
P value summary	ns	ns	ns	ns	*	*	ns	ns	*

	β1AR (saturator)								
	Control			300uM imipramine			MI		
	27.077	11.4385	5.98084	8.563774	14.9938	10.2912	8.680941	10.75639	10.87888
	21.127	8.74238	9.95191	10.15255	18.5618	7.3558	5.344075	7.685393	7.325043
	17.8658	9.2134	10.2971	5.0148	14.1197	21.6419	8.669651	7.428993	7.899065
	14.2073	11.8529	9.95026		13.0578	8.0144	11.72372	13.64537	12.59411
	13.8823		7.90547		12.6192	10.391	6.956681	8.658414	7.488512
	18.4813		9.45752		14.6615		8.533629	12.52583	13.34789
					10.0955		9.467202	12.74366	9.940072
					16.9284		12.77738	10.15488	7.075605
					18.0936		9.8809	11.90328	6.883458
					11.7498		13.71937	10.88209	9.142513
					10.4471		10.791	12.24705	7.560408
					11.1375		10.46547	11.1897	8.997681
					16.4821		11.08631	9.956739	11.61583
					13.8881		11.53808	9.576379	10.64569
							16.16061	9.667641	11.31691
							14.54196	14.41106	8.953576
							3.279839	13.16914	6.338497
							5.653154		
							6.707113		
							4.539796		
							4.430563		
							5.85462		
							10.98184		
							4.616886		
							11.55599		
							10.75423		
							10.13734		
Number of values	6	4	6	3	14	5	27	17	17
Minimum	13.88	8.742	5.981	5.015	10.1	7.356	3.28	7.429	6.338
Maximum	27.08	11.85	10.3	10.15	18.56	21.64	16.16	14.41	13.35
Range	13.19	3.11	4.316	5.138	8.466	14.29	12.88	6.982	7.009
Mean	18.77	10.31	8.924	7.91	14.06	11.54	9.217	10.98	9.294
Std. Deviation	4.902	1.561	1.672	2.63	2.74	5.807	3.361	2.031	2.141
Std. Error of Mean	2.001	0.7807	0.6826	1.519	0.7324	2.597	0.6468	0.4926	0.5193
Shapiro-Wilk test									
W	0.9141	0.8599	0.8236	0.9537	0.9591	0.7517	0.9674	0.9761	0.9419
P value	0.4636	0.2599	0.0948	0.5859	0.7081	0.0309	0.5348	0.9134	0.3415
Passed normality test (alpha=0.05)?	Yes	Yes	Yes	Yes	Yes	No	Yes	Yes	Yes
P value summary	ns	ns	ns	ns	ns	*	ns	ns	ns

beta 1 (ISO)						
Dunn's multiple comparisons test	Mean rank diff.	Significant?	Summary	Adjusted P Value		
Control vs. Imipramine	-15.58	No	ns	0.30315549	A-B	
Control vs. MI	5.305	No	ns	>0.999999999999999	A-C	
Imipramine vs. MI	20.89	Yes	*	0.015730249	B-C	
Test details	Mean rank 1	Mean rank 2	Mean rank d	n1	n2	
Control vs. Imipramine	48.13	63.71	-15.58	16	17	
Control vs. MI	48.13	42.82	5.305	16	61	
Imipramine vs. MI	63.71	42.82	20.89	17	61	

beta 1 (Saturator)						
Dunn's multiple comparisons test	Mean rank diff.	Significant?	Summary	Adjusted P Value		
Control vs. Imipramine	-4.813	No	ns	>0.9999	A-B	
Control vs. MI	15.84	No	ns	0.149	A-C	
Imipramine vs. MI	20.65	Yes	*	0.0115	B-C	
Test details	Mean rank 1	Mean rank 2	Mean rank diff.	n1	n2	
Control vs. Imipramine	58.69	63.5	-4.813	16	22	
Control vs. MI	58.69	42.85	15.84	16	61	
Imipramine vs. MI	63.5	42.85	20.65	22	61	

	beta2 (ISO)										
	Control				300uM imipramine				MI		
	4.536398	13.993	3.173014	4.636471	1.19088	8.120141	3.6806	4.5567	0.516761	3.177347	4.468872
	6.168649	8.8765	4.906172	7.003619	4.126971	9.5538	11.1196	1.5225	1.866317	5.927479	3.71441
		6.1285	8.666293	7.638592	1.6614	2.3536		1.3394	0.609744	5.709252	6.021251
			5.263768	4.331727	2.375727	2.404964		2.1441	0.533676	3.486113	3.335364
			5.976253	5.931856	0.406532	9.6637		1.142	0.345295	6.142524	
			4.994458	6.988947	0.59435			1.701		2.212463	
			6.108276	9.113867	0.031554			1.4779		6.783672	
			5.18159	6.352767				3.3553		7.813715	
			7.04163							2.658281	
										7.873474	
Number of values	2	3	9	8	7	5	2	8	5	10	4
Minimum	4.536	6.129	3.173	4.332	0.03155	2.354	3.681	1.142	0.3453	2.212	3.335
Maximum	6.169	13.99	8.666	9.114	4.127	9.664	11.12	4.557	1.866	7.873	6.021
Range	1.632	7.865	5.493	4.782	4.095	7.31	7.439	3.415	1.521	5.661	2.686
Mean	5.353	9.666	5.701	6.5	1.484	6.419	7.4	2.155	0.7744	5.178	4.385
Std. Deviation	1.154	3.991	1.534	1.565	1.412	3.738	5.26	1.193	0.618	2.123	1.188
Std. Error of Mean	0.8161	2.304	0.5112	0.5532	0.5336	1.672	3.72	0.4218	0.2764	0.6714	0.5941
Shapiro-Wilk test											
W	N too small	0.9707	0.9476	0.9649	0.9101	0.7756	N too sma	0.804	0.6989	0.9026	0.9175
P value		0.6712	0.6642	0.855	0.3965	0.0505		0.0316	0.0093	0.2342	0.5232
Passed normality test (alpha=0.05)?		Yes	Yes	Yes	Yes	Yes		No	No	Yes	Yes
P value summary		ns	ns	ns	ns	ns		*	**	ns	ns

beta2 (Saturator)										
Control				300uM imipriamine				MI		
17.20087	15.62115	15.89811	30.5486	2.590141	57.70027	17.2558	20.934	1.437263	11.07607	12.95381
14.0213	20.48024	17.45158	18.5568	19.56796	40.8938	34.8353	8.2885	5.256579	12.31625	12.23168
	17.35337	12.8572	7.9903	10.9975	12.4111		9.1892	1.592856	10.66087	12.05445
	15.31743	11.5855		10.53221	17.56123		7.4278	1.762186	7.529029	9.197444
	17.70642	14.79445		8.907366	36.7377		10.082	0.7864842	12.58563	
	18.18202	13.17142		19.01958			15.9149		9.756729	
	19.17166	15.00965		10.65471			11.9335		13.91652	
	20.51887	13.76279					15.5659		13.14463	
	20.01645								7.872898	
									16.35169	
2	9	8	3	7	5	2	8	5	10	4
14.02	15.32	11.59	7.99	2.59	12.41	17.26	7.428	0.7865	7.529	9.197
17.2	20.52	17.45	30.55	19.57	57.7	34.84	20.93	5.257	16.35	12.95
3.18	5.201	5.866	22.56	16.98	45.29	17.58	13.51	4.47	8.823	3.756
15.61	18.26	14.32	19.03	11.75	33.06	26.05	12.42	2.167	11.52	11.61
2.248	1.959	1.861	11.29	5.905	18.36	12.43	4.671	1.766	2.724	1.654
1.59	0.653	0.658	6.516	2.232	8.212	8.79	1.651	0.7899	0.8613	0.8272
N too small	0.9165	0.9868	0.9987	0.8983	0.9468	N too small	0.9125	0.7453	0.9734	0.8304
	0.3638	0.9886	0.9304	0.3211	0.714		0.3721	0.0269	0.9208	0.1689
	Yes	Yes	Yes	Yes	Yes		Yes	No	Yes	Yes
	ns	ns	ns	ns	ns		ns	*	ns	ns

beta 2 (ISO)					
Dunn's multiple comparisons test	Mean rank diff.	Significant?	Summary	Adjusted P Value	
Control vs. Imipramine	20.95	Yes	***	0.000449	A-B
Control vs. MI	16.43	Yes	*	0.012626	A-C
Imipramine vs. MI	-4.524	No	ns	>0.9999999	B-C
Test details	Mean rank 1	Mean rank 2	Mean rank d	n1	n2
Control vs. Imipramine	44.27	23.32	20.95	22	22
Control vs. MI	44.27	27.84	16.43	22	19
Imipramine vs. MI	23.32	27.84	-4.524	22	19

beta 2 (Saturator)					
Dunn's multiple comparisons test	Mean rank diff.	Significant?	Summary	Adjusted P Value	
Control vs. Imipriamine	2.695	No	ns	>0.999999999	A-B
Control vs. MI	20.91	Yes	****	9.25003E-05	A-C
Imipriamine vs. MI	18.21	Yes	**	0.003741934	B-C
Test details	Mean rank 1	Mean rank 2	Mean rank	n1	n2
Control vs. Imipriamine	35.91	33.21	2.695	22	14
Control vs. MI	35.91	15	20.91	22	19
Imipriamine vs. MI	33.21	15	18.21	14	19

Supplementary figure 1B

tubulin quantifiacation area

	Control			2µM Vinblastine		
	0.517	0.404	0.281	0.016	0.033	0.068
	0.505	0.595	0.501	0.139	0.16	0.023
	0.42	0.57	0.422		0.097	0.051
	0.524	0.495	0.497		0.252	0.076
	0.668	0.626	0.474			
			0.523			
Number of values	5	5	6	2	4	4
Minimum	0.42	0.404	0.281	0.016	0.033	0.023
Maximum	0.668	0.626	0.523	0.139	0.252	0.076
Range	0.248	0.222	0.242	0.123	0.219	0.053
Mean	0.5268	0.538	0.4497	0.0775	0.1355	0.0545
Std. Deviation	0.0893	0.089	0.0895	0.087	0.0934	0.02344
Std. Error of Mean	0.04	0.04	0.0365	0.0615	0.0467	0.01172
Shapiro-Wilk test						
W	0.8991	0.926	0.8071	N too sm	0.9907	0.9344
P value	0.405	0.571	0.068		0.961	0.6203
Passed normality test (alpha=0.05)?	Yes	Yes	Yes		Yes	Yes
P value summary	ns	ns	ns		ns	ns

Unpaired t test	
P value	<.001
P value summary	***
Significantly different (P < 0.05)?	Yes
One- or two-tailed P value?	Two-tailed
t, df	t=10.2, df=33
How big is the difference?	
Mean of column A	0.501
Mean of column B	0.247
Difference between means (B - A) ± SEM	-0.254 ± 0.0250
95% confidence interval	-0.305 to -0.204
R squared (eta squared)	0.759
F test to compare variances	
F, DFn, Dfd	3.20, 15, 18
P value	0.021
P value summary	*
Significantly different (P < 0.05)?	Yes
Data analyzed	
Sample size, column A	16
Sample size, column B	19

Figure 1C

Total RNA gene expression

	beta1		beta2	
	Control	2μM Vinblastine	Control	2μM Vinblastine
	0.989415	1.230674	1.354552	1.05743
	0.9971088	0.8970364	0.1780825	0.6252832
	1.290235	0.700896	1.078065	0.9568402
	0.8804597	1.514569	1.669	1.272731
	0.8922788	0.5002044		0.3179145
	Mann Whitney test		Mann Whitney test	
	P value	0.8413	P value	0.4127
	Exact or approximate P value?	Exact	Exact or approximate P value?	Exact
	P value summary	ns	P value summary	ns
	Significantly different (P < 0.05)?	No	Significantly different (P < 0.05)?	No
	One- or two-tailed P value?	Two-tailed	One- or two-tailed P value?	Two-tailed
	Sum of ranks in column A,B	29 , 26	Sum of ranks in column A,B	24 , 21
	Mann-Whitney U	11	Mann-Whitney U	6
	Difference between medians		Difference between medians	
	Median of column A	0.9894, n=5	Median of column A	1.216, n=4
	Median of column B	0.8970, n=5	Median of column B	0.9568, n=5
	Difference: Actual	-0.09238	Difference: Actual	-0.2595
	Difference: Hodges-Lehmann	-0.09238	Difference: Hodges-Lehmann	-0.3467

Supplementary figure 2D

	mean % cell area											
	DMSO				5μM Nocodazole				10μM Nocodazole			
	Rat 1	Rat 2	Rat 3	Rat 4	Rat 1	Rat 2	Rat 3	Rat 4	Rat 1	Rat 2	Rat 3	Rat 4
	0.34616	0.54139	0.37869	0.51561	0.05899	0.00213	0.02526	0.03187	0.00201	0.00066	0.00346	0.00392
	0.35543	0.49715	0.43818	0.50297	0.01693	0.00518	0.0243	0.04318	0.00194	0.00035	0.00937	0.00073
	0.45184	0.55426	0.505	0.48831	0.01002	0.00854	0.02506	0.02459	0.00568	0.0019	0.00881	0.00126
	0.26093	0.39898	0.47516	0.40256	0.01055	0.00406	0.0426		0.00251		0.00805	
		0.42965									0.01002	
Number of values	4	5	4	4	4	4	4	3	4	3	5	3
Minimum	0.2609	0.399	0.3787	0.4026	0.01002	0.00213	0.0243	0.02459	0.00194	0.00035	0.00346	0.00073
Maximum	0.4518	0.5543	0.505	0.5156	0.05899	0.00854	0.0426	0.04318	0.00568	0.0019	0.01002	0.00392
Range	0.1909	0.1553	0.1263	0.1131	0.04897	0.00641	0.01831	0.01859	0.00374	0.00155	0.00657	0.00319
Mean	0.3536	0.4843	0.4493	0.4774	0.02412	0.00498	0.02931	0.03321	0.00303	0.00097	0.00794	0.00197
Std. Deviation	0.0781	0.06816	0.05441	0.0511	0.02346	0.00269	0.00888	0.00937	0.00178	0.00082	0.00261	0.00171
Std. Error of Mean	0.03905	0.03048	0.0272	0.02555	0.01173	0.00134	0.00444	0.00541	0.00089	0.00047	0.00117	0.00099
Shapiro-Wilk test												
W	0.9631	0.9118	0.9731	0.8197	0.7302	0.9724	0.6732	0.9846	0.7393	0.8934	0.8043	0.8716
P value	0.7983	0.4784	0.8603	0.1425	0.0248	0.8565	0.0054	0.7622	0.0305	0.3648	0.0878	0.3
Passed normality test (alpha=0.05)?	Yes	Yes	Yes	Yes	No	Yes	No	Yes	No	Yes	Yes	Yes
P value summary	ns	ns	ns	ns	*	ns	**	ns	*	ns	ns	ns
Dunnett's multiple comparisons test	Mean Diff.	95.00% CI	Significant	Summary	Adjusted P Value							
DMSO vs. 5μM Nocodazole	0.4191	0.3520 to	Yes	****	<0.0001							
DMSO vs. 10μM Nocodazole	0.4382	0.3709 to	Yes	****	<0.0001							
Test details	Mean 1	Mean 2	Mean Diff.	SE of diff.	N1	N2	q	DF				
DMSO vs. 5μM Nocodazole	0.4418	0.02266	0.4191	0.02563	17	15	16.35	9				
DMSO vs. 10μM Nocodazole	0.4418	0.00365	0.4382	0.02568	17	15	17.07	9				

Supplementary figure 3C

Nocodazole PI

	DMSO beta1				Noc beta1				DMSO beta2				Noc beta2			
	0.122455	0.047323	0.0849	0.05487	0.2098	0.02948	0.014225	0.05274	0.1361	0.1184	0.08059	0.1835	0.1144	0.06768	0.133425	0.1562
	0.089775	0.145985	0.07583	0.13035	0.05374	0.105215	0.1426	0.14232	0.20365	0.096345	0.14545	0.097045	0.2471	0.29425	0.05237	0.2165
	0.12643	0.07963	0.014835	0.06243	0.2046	0.1545	0.28375	0.1117	0.10955	0.15155	0.23025	0.113795	0.125235	0.2385	0.14589	0.083795
	0.129	0.095235	0.081395	0.068915	0.07476	0.1173	0.071475	0.08495	0.1519	0.26175	0.1573	0.268	0.2585	0.3059	0.065625	0.04095
	0.06487	0.13068	0.07486	0.108865	0.271	0.181	0.1651	0.14675	0.1292	0.11241	0.12062	0.1589	0.17185	0.146225	0.12645	0.2069
	0.152355	0.04345	0.045485	0.025624	0.269	0.15335	0.091295	0.06799	0.07047	0.24415	0.2276	0.09508	0.1574	0.1575	0.09884	0.06786
	0.07694	0.081265	0.0475	0.11269	0.20115	0.084105	0.034935	0.08119	0.05345	0.0972	0.24755	0.1982	0.10614	0.143245	0.1588	0.09265
	0.075345	0.088935	0.07497	0.066715	0.08884		0.066445	0.14859	0.09717	0.05877	0.21615	0.1419	0.1813	0.152325	0.21995	0.28365
	0.13868	0.1375	0.108605	0.05086	0.15995		0.05956	0.15095	0.2859		0.19155	0.274	0.069763		0.1697	0.044705
				0.035355			0.046485	0.035722	0.05634			0.13645			0.08657	0.160115
							0.101975	0.062105				0.27955			0.17345	0.2339
							0.05559								0.15076	
Number of values	9	9	9	10	9	7	12	11	10	8	9	11	9	8	12	11
Minimum	0.06487	0.04345	0.01484	0.02562	0.05374	0.02948	0.01422	0.03572	0.05345	0.05877	0.08059	0.09508	0.06976	0.06768	0.05237	0.04095
Maximum	0.1524	0.146	0.1086	0.1304	0.271	0.181	0.2838	0.151	0.2859	0.2618	0.2476	0.2796	0.2585	0.3059	0.22	0.2837
Range	0.08749	0.1025	0.09377	0.1047	0.2173	0.1515	0.2695	0.1152	0.2325	0.203	0.167	0.1845	0.1887	0.2382	0.1676	0.2427
Mean	0.1084	0.09444	0.0676	0.07167	0.1703	0.1179	0.09445	0.09864	0.1294	0.1426	0.1797	0.1769	0.1591	0.1882	0.1318	0.1443
Std. Deviation	0.03186	0.0372	0.02744	0.03461	0.08142	0.05115	0.07356	0.04296	0.07193	0.07301	0.05691	0.07	0.06346	0.08295	0.04869	0.08351
Std. Error of Mean	0.01062	0.0124	0.009146	0.01094	0.02714	0.01933	0.02124	0.01295	0.02275	0.02581	0.01897	0.02111	0.02115	0.02933	0.01406	0.02518
Shapiro-Wilk test																
W	0.9075	0.9225	0.9287	0.9248	0.9053	0.9564	0.8416	0.8907	0.9011	0.8627	0.9358	0.8878	0.9453	0.9012	0.9724	0.9293
P value	0.2985	0.4133	0.4689	0.3989	0.2841	0.7876	0.029	0.1419	0.225	0.1278	0.538	0.1304	0.6388	0.2965	0.934	0.4035
Passed normality test (alpha=0.05)?	Yes	Yes	Yes	Yes	Yes	Yes	No	Yes	Yes	Yes	Yes	Yes	Yes	Yes	Yes	Yes
P value summary	ns	ns	ns	ns	ns	ns	*	ns	ns	ns	ns	ns	ns	ns	ns	ns

Sidak's multiple comparisons test	Mean Diff.	95.00% CI	Significant	Summary	Adjusted P Value
DMSO beta1 vs. DMSO beta2	-0.0722	-0.1204 to	Yes	**	0.004802
Noc beta1 vs. Noc beta2	-0.03532	-0.08293 t	No	ns	0.158327

Test details	Mean 1	Mean 2	Mean Diff.	SE of diff.	N1	N2	t	DF
DMSO beta1 vs. DMSO beta2	0.08533	0.1575	-0.0722	0.01886	37	38	3.828	12
Noc beta1 vs. Noc beta2	0.1187	0.1541	-0.03532	0.01865	39	40	1.894	12

Nocodazole DI

	DMSO beta1				Noc beta1				DMSO beta2				Noc beta2			
	0.110485	0.104195	0.07562	0.104195	0.045315	0.065635	0.16875	0.02177	0.2323	0.2772	0.25185	0.22595	0.24315	0.2208	0.21725	0.172315
	0.074275	0.074625	0.18799	0.044015	0.081585	0.06548	0.02257	0.014215	0.2563	0.2603	0.25015	0.2778	0.144325	0.2261	0.13742	0.20805
	0.1067	0.058795	0.137235	0.053384	0.081765	0.13175	0.03794	0.03484	0.2279	0.2881	0.35045	0.2487	0.164	0.2436	0.18765	0.164
	0.092045	0.095165	0.049785	0.12475	0.125945	0.0847	0.024875	0.04952	0.2219	0.2971	0.3193	0.2539	0.279	0.10318	0.1897	0.18215
	0.09064	0.09869	0.086295	0.12665	0.05131	0.025245	0.0548	0.014312	0.23575	0.27025	0.2937	0.24285	0.3057	0.17575	0.22495	0.087025
	0.078935	0.093135	0.05713	0.08018	0.17045	0.08838	0.0225	0.0879	0.2446	0.38815	0.2879	0.24655	0.2646	0.23335	0.15125	0.2253
	0.06746	0.0733	0.13585	0.101125	0.10839	0.080025	0.06003	0.082335	0.27615	0.2999	0.31585	0.29395	0.2949	0.2179	0.20635	0.1962
	0.10495	0.076825	0.064975	0.047645	0.032045	0.02334	0.011752	0.019505	0.22495	0.25175	0.27165	0.2405	0.24395	0.16565	0.050805	0.163845
	0.06342	0.07528	0.05369	0.03046	0.07895	0.002366	0.022325	0.03494		0.35	0.28305	0.27065	0.3004	0.12442	0.15338	0.1376
			0.04721	0.09983	0.195639	0.03112	0.00791	0.00792		0.2817	0.2498	0.25575	0.17595	0.12311	0.16	0.12985
			0.050655				0.010344					0.2945				0.12272
							0.0123									
Number of values	9	9	11	10	10	10	10	12	8	10	10	11	10	10	10	11
Minimum	0.06342	0.0588	0.04721	0.03046	0.03205	0.002366	0.00791	0.00792	0.2219	0.2518	0.2498	0.226	0.1443	0.1032	0.05081	0.08703
Maximum	0.1105	0.1042	0.188	0.1267	0.1956	0.1318	0.1688	0.0879	0.2762	0.3882	0.3505	0.2945	0.3057	0.2436	0.225	0.2253
Range	0.04707	0.0454	0.1408	0.09619	0.1636	0.1294	0.1608	0.07998	0.05425	0.1364	0.1007	0.06855	0.1614	0.1404	0.1741	0.1383
Mean	0.08766	0.08333	0.08604	0.08122	0.09714	0.0598	0.04335	0.03249	0.24	0.2964	0.2874	0.2592	0.2416	0.1834	0.1679	0.1626
Std. Deviation	0.01754	0.01497	0.04691	0.03511	0.05361	0.03906	0.04722	0.02748	0.01841	0.04208	0.03362	0.02228	0.05976	0.05213	0.0508	0.04075
Std. Error of Mean	0.005847	0.00499	0.01414	0.0111	0.01695	0.01235	0.01493	0.007933	0.006508	0.01331	0.01063	0.006717	0.0189	0.01648	0.01606	0.01229
Shapiro-Wilk test																
W	0.9265	0.9251	0.8044	0.9108	0.9246	0.9526	0.6834	0.8039	0.8868	0.8562	0.9269	0.9303	0.8801	0.8847	0.8873	0.982
P value	0.4491	0.4365	0.0108	0.2865	0.3965	0.6997	0.0006	0.0104	0.2185	0.0689	0.4184	0.4138	0.131	0.1476	0.158	0.976
Passed normality test (alpha=0.05)?	Yes	Yes	No	Yes	Yes	Yes	No	No	Yes	Yes	Yes	Yes	Yes	Yes	Yes	Yes
P value summary	ns	ns	*	ns	ns	ns	***	*	ns	ns	ns	ns	ns	ns	ns	ns

Tukey's multiple comparisons test	Mean Diff.	95.00% CI of diff.	Significant?	Summary	Adjusted P Value
DMSO:β_{1AR} vs. DMSO:β_{2AR}	-0.187	-0.214 to -0.161	Yes	****	9.4E-14
DMSO:β_{1AR} vs. 10µM Nocodazole:β_{1AR}	0.0276	0.00155 to 0.0536	Yes	*	0.03324
DMSO:β_{2AR} vs. 10µM Nocodazole:β_{2AR}	0.0838	0.0576 to 0.110	Yes	****	3.88E-13
10µM Nocodazole:β_{1AR} vs. 10µM Nocodazole:β_{2AR}	-0.131	-0.157 to -0.106	Yes	****	9.4E-14

Test details	Mean 1	Mean 2	Mean Diff.	SE of diff.	N1	N2	q	DF
DMSO:β_{1AR} vs. DMSO:β_{2AR}	0.0846	0.272	-0.187	0.0102	39	39	26	157
DMSO:β_{1AR} vs. 10µM Nocodazole:β_{1AR}	0.0846	0.057	0.0276	0.01	39	42	3.89	157
DMSO:β_{2AR} vs. 10µM Nocodazole:β_{2AR}	0.272	0.188	0.0838	0.0101	39	41	11.8	157
10µM Nocodazole:β_{1AR} vs. 10µM Nocodazole:β_{2AR}	0.057	0.188	-0.131	0.0099	42	41	18.8	157

Figure 3D

	beta1									
	DMSO					10μM Nocodazole				
	54.6718	40.3048	73.72511	67.6188	46.5672	50.2678	36.4816	34.093	53.1777	41.538
	47.7992	31.55189	61.85355	69.8102	36.6967	49.0222	27.0912	36.605	55.5628	55.5877
	52.9243	43.76106	69.01777	68.0878	55.2996	45.277	34.4	42.8731	77.7209	37.9276
	43.5564	74.01507	59.16003	75.4753	65.0871	38.6188	69.0155	61.2049	36.2203	
	50.075	78.8989	43.06662		37.0657		22.6488	48.3148		
	29.3256	54.0349	32.02986		41.68		43.0389	57.4981		
	30.6544	34.88652	46.76664		74.6824		50.0615	69.6805		
	61.8874	43.12898			61.1853		45.7824			
	45.5795	86.66386			52.0105		55.8493			
	56.7879	79.72423					56.2035			
	61.9441	75.66269					58.7715			
	61.8099	72.2652					65.692			
	39.7434	64.533					67.4426			
	57.6027	76.53098					70.9026			
		54.20075					75.4211			
		59.29075					22.5404			
		38.24613					31.3496			
		68.12367								
Number of values	14	18	7	4	9	4	17	7	4	3
Minimum	29.33	31.55	32.03	67.62	36.7	38.62	22.54	34.09	36.22	37.93
Maximum	61.94	86.66	73.73	75.48	74.68	50.27	75.42	69.68	77.72	55.59
Range	32.62	55.11	41.7	7.856	37.99	11.65	52.88	35.59	41.5	17.66
Mean	49.6	59.77	55.09	70.25	52.25	45.8	48.98	50.04	55.67	45.02
Std. Deviation	10.84	17.7	15	3.61	13.1	5.234	17.67	13.26	17.04	9.33
Std. Error of Mean	2.898	4.172	5.668	1.805	4.368	2.617	4.285	5.012	8.518	5.387
Shapiro-Wilk test										
W	0.9157	0.9282	0.9592	0.827	0.951	0.906	0.9379	0.9519	0.9604	0.8957
P value	0.1904	0.1803	0.8116	0.16	0.7008	0.4612	0.294	0.7472	0.7815	0.3719
Passed normality test (alpha=0.05)?	Yes	Yes	Yes	Yes	Yes	Yes	Yes	Yes	Yes	Yes
P value summary	ns	ns	ns	ns	ns	ns	ns	ns	ns	ns

	beta2							
	DMSO				10μM Nocodazole			
	21.7267	41.0005	28.1178	38.5146	10.7792	6.90879	17.7205	3.35634
	25.7444	32.0511	29.573	38.4052	20.362	9.14199	22.1533	8.92371
	32.4255	36.0738	29.227	38.692	6.24042	7.86481	15.5835	3.74395
		38.5297	28.7937	39.5521	10.2919		15.0411	11.7506
		30.5741	22.7361		10.398		9.70017	9.16362
		24.414	37.2873				4.9737	14.8877
							11.0461	
							20.0833	
Number of values	3	6	6	4	5	3	8	6
Minimum	21.73	24.41	22.74	38.41	6.24	6.909	4.974	3.356
Maximum	32.43	41	37.29	39.55	20.36	9.142	22.15	14.89
Range	10.7	16.59	14.55	1.147	14.12	2.233	17.18	11.53
Mean	26.63	33.77	29.29	38.79	11.61	7.972	14.54	8.638
Std. Deviation	5.404	6.016	4.661	0.521	5.228	1.12	5.698	4.493
Std. Error of Mean	3.12	2.456	1.903	0.2605	2.338	0.6469	2.015	1.834
Shapiro-Wilk test								
W								
P value	0.9798	0.9707	0.8847	0.8212	0.8261	0.9932	0.9739	0.9288
Passed normality test (alpha=0.05)?	0.7274	0.8969	0.2914	0.146	0.13	0.8418	0.9269	0.5706
P value summary	Yes	Yes	Yes	Yes	Yes	Yes	Yes	Yes

Table Analyzed	B1AR nested data
Column B	10μM Nocodazole
vs.	vs.
Column A	DMSO
Nested t test	
P value	
P value summary	ns
Significantly different (P < 0.05)?	No
One- or two-tailed P value?	Two-tailed
t, df	t=1.853, df=8
F, DFn, Dfd	3.433, 1, 8
How big is the difference?	
Mean of column B	
Mean of column A	
Difference between means (B - A) ± SEM	-6.708 ± 3.621
95% confidence interval	-15.06 to 1.641
Random effects	SD
Variation within subcolumns	
Variation among subcolumn means	
Do the subcolumns differ (within each column)?	
Chi-square, df	0.1775, 1
P value	
P value summary	ns
Is there significant difference between subcolumns (P < 0.05	No
Data analyzed	
Number of treatments (columns)	
Number of subjects (subcolumns)	
Total number of values	

supplementary figure 4A

beta1_2 Density_Regularity

beta1 Density

	Control					Vin				
	19.164	22.567	21.53	18.734	23.351	4.863	17.493	15.938	18.993	11.88
	16.777	16.456	22.772	22.785	26.12	16.941	8.354	16.49	15.427	20.794
	17.302	20.241	24.818	13.094	21.892	18.421	14.288	13.497	15.536	2.23
	22.976	18.584	25.09		18.666	10.598		10.366	8.545	15.993
						13.647				
Number of	4	4	4	3	4	5	3	4	4	4
Minimum	16.78	16.46	21.53	13.09	18.67	4.863	8.354	10.37	8.545	2.23
Maximum	22.98	22.57	25.09	22.79	26.12	18.42	17.49	16.49	18.99	20.79
Range	6.199	6.111	3.56	9.691	7.454	13.56	9.139	6.124	10.45	18.56
Mean	19.05	19.46	23.55	18.2	22.51	12.89	13.38	14.07	14.63	12.72
Std. Devia	2.808	2.586	1.7	4.867	3.104	5.414	4.637	2.792	4.379	7.888
Std. Error	1.404	1.293	0.8498	2.81	1.552	2.421	2.677	1.396	2.189	3.944
Shapiro-Wilk test										
W	0.8832	0.9988	0.8964	0.9911	0.9962	0.9478	0.9711	0.9104	0.9009	0.9681
P value	0.3523	0.9963	0.4133	0.8197	0.9868	0.7212	0.6739	0.4843	0.4357	0.8299
Passed no	Yes	Yes	Yes	Yes	Yes	Yes	Yes	Yes	Yes	Yes
P value su	ns	ns	ns	ns	ns	ns	ns	ns	ns	ns

beta2_density

	Control					Vin		
	11.908	20.712	14.526	23.849	27.511	15.556	26.741	9.561
	19.948	33.158	16.095	14.335	25.527	2.176	18.543	12.153
	17.657	27.068	13.824	18.53	27.28	4.112	15.44	15.986
	15.202	19.784		24.79	28.514		19.662	7.741
		18.768		16.6				
Number of	4	5	3	5	4	3	4	4
Minimum	11.91	18.77	13.82	14.34	25.53	2.176	15.44	7.741
Maximum	19.95	33.16	16.1	24.79	28.51	15.56	26.74	15.99
Range	8.04	14.39	2.271	10.46	2.987	13.38	11.3	8.245
Mean	16.18	23.9	14.82	19.62	27.21	7.281	20.1	11.36
Std. Devia	3.444	6.107	1.163	4.551	1.242	7.231	4.776	3.576
Std. Error	1.722	2.731	0.6713	2.035	0.621	4.175	2.388	1.788
Shapiro-Wilk test								
W	0.9895	0.8593	0.9537	0.9132	0.9449	0.8559	0.9242	0.9692
P value	0.9549	0.2256	0.5857	0.4871	0.6845	0.2564	0.5609	0.8366
Passed no	Yes	Yes	Yes	Yes	Yes	Yes	Yes	Yes
P value su	ns	ns	ns	ns	ns	ns	ns	ns

**beta1 density**		
Unpaired t test with Welch's correction		
P value	5.21E-06	
P value summary	****	
Significantly different (P < 0.05)?	Yes	
One- or two-tailed P value?	Two-tailed	
Welch-corrected t, df	t=5.389, df=34.33	
How big is the difference?		
Mean of column A	20.68	
Mean of column B	13.51	
Difference between means (B - A) ± SEM	-7.165 ± 1.330	
95% confidence interval	-9.866 to -4.464	
R squared (eta squared)	0.4583	
F test to compare variances		Variance
F, DFn, Dfd	1.979, 19,	17.52
P value	0.153893	0
P value summary	ns	
Significantly different (P < 0.05)?	No	
Data analyzed		
Sample size, column A	19	
Sample size, column B	20	

**beta2 density**		
Unpaired t test with Welch's correction		
P value	0.00988637	
P value summary	**	
Significantly different (P < 0.05)?	Yes	
One- or two-tailed P value?	Two-tailed	
Welch-corrected t, df	t=2.902, df=17.10	
How big is the difference?		
Mean of column A	20.74	
Mean of column B	13.42	
Difference between means (B - A) ± SEM	-7.318 ± 2.522	
95% confidence interval	-12.64 to -1.999	
R squared (eta squared)	0.3299	
F test to compare variances		Variance
F, DFn, Dfd	1.513, 10, 20	20.32
P value	0.412659128	26.45
P value summary	ns	
Significantly different (P < 0.05)?	No	
Data analyzed		
Sample size, column A	21	
Sample size, column B	11	

	beta1 Regularity										beta2 Regularity							
	Control					Vin					Control					Vin		
	611916	365290	142190	719239	283039	173711	94376.4	136636	236856	91641.5	102375	181392	92303.1	120928	104830	78253	86691	90462.1
	379491	167193	258309	113303	216750	60489.2	274613	61817.7	230234	151475	124854	80513.6	63763.9	211620	168245	72839.2	105937	53363.6
	374234	192451	238004	251564	145400	68736.6		262016	193919	152873	102493	62991	100845	317306	191361	88542.2	29181.3	78867.9
	363867	648567	411021		479239	182466			76330.2			68172.9		87666.9	126348		47002.8	
												131871		72759				
Number of	4	4	4	3	4	4	2	3	4	3	3	5	3	5	4	3	4	3
Minimum	363867	167193	142190	113303	145400	60489	94376	61818	76330	91642	102375	62991	63764	72759	104830	72839	29181	53364
Maximum	611916	648567	411021	719239	479239	182466	274613	262016	236856	152873	124854	181392	100845	317306	191361	88542	105937	90462
Range	248049	481374	268831	605936	333839	121977	180237	200198	160526	61232	22479	118401	37081	244547	86531	15703	76756	37099
Mean	432377	343375	262381	361369	281107	121351	184495	153490	184335	131997	109907	104988	85637	162056	147696	79878	67203	74231
Std. Devia	119869	221691	111281	317541	143548	65699	127447	101158	74436	34955	12944	50691	19418	102174	39252	7977	35279	18979
Std. Error	59934	110845	55641	183332	71774	32849	90118	58403	37218	20182	7473	22670	11211	45694	19626	4605	17639	10957
Shapiro-Wilk test																		
W	0.6811	0.877	0.9527	0.9103	0.9354	0.7923	N too smal	0.9792	0.8146	0.7671	0.7539	0.8609	0.9116	0.887	0.955	0.9689	0.9435	0.9552
P value	0.0068	0.3258	0.7333	0.4191	0.6262	0.0892		0.7235	0.1311	0.0382	0.0087	0.2316	0.4235	0.3421	0.7474	0.6612	0.6757	0.5928
Passed no	No	Yes	Yes	Yes	Yes	Yes		Yes	Yes	No	No	Yes	Yes	Yes	Yes	Yes	Yes	Yes
P value su	**	ns	ns	ns	ns	ns		ns	ns	*	**	ns	ns	ns	ns	ns	ns	ns

beta1 Regularity	
Unpaired t test with Welch's correction	
P value	0.000406
P value summary	***
Significantly different (P < 0.05)?	Yes
One- or two-tailed P value?	Two-tailed
Welch-corrected t, df	t=4.082, df=24.79
How big is the difference?	
Mean of column A	334793
Mean of column B	153012
Difference between means (B - A) ± SEM	-181781 ± 44529
95% confidence interval	-273530 to -90032
R squared (eta squared)	0.402
F test to compare variances	
F, DFn, Dfd	5.879, 18, 15
P value	0.001168
P value summary	**
Significantly different (P < 0.05)?	Yes
Data analyzed	
Sample size, column A	19
Sample size, column B	16

beta2 Regularity	
Mann Whitney test	
P value	0.007175966
Exact or approximate P value?	Exact
P value summary	**
Significantly different (P < 0.05)?	Yes
One- or two-tailed P value?	Two-tailed
Sum of ranks in column A,B	370 , 95
Mann-Whitney U	40
Difference between medians	
Median of column A	103662, n=20
Median of column B	78560, n=10
Difference: Actual	-25101
Difference: Hodges-Lehmann	-37059

supplementary figure 6C

	Imp Density						
	Control			300µM Impramine			
	12.334	17.943	20.468	0.106	3.296	0.797	2.02
	15.976	19.67	15.843	3.137	1.781	2.366	6.991
	13.45	20.574	19.591	3.509	3.695	3.137	2.711
	16.906	18.298	17.092	5.875	7.443	10.898	7.31
	11.776	14.939	17.119	2.446	3.349	0.665	1.781
	18.129	20.229	21.106	5.954	2.446	2.392	1.249
	17.251	10.154		12.148	6.725	5.104	1.568
				5.051	2.951	3.562	5.472
				11.191			7.602
				6.539			
Number of values	7	7	6	10	8	8	9
Minimum	11.78	10.15	15.84	0.106	1.781	0.665	1.249
Maximum	18.13	20.57	21.11	12.15	7.443	10.9	7.602
Range	6.353	10.42	5.263	12.04	5.662	10.23	6.353
Mean	15.12	17.4	18.54	5.596	3.961	3.615	4.078
Std. Deviation	2.558	3.717	2.135	3.743	2.026	3.277	2.715
Std. Error of Mean	0.9667	1.405	0.8717	1.184	0.7161	1.159	0.9051
Shapiro-Wilk test							
W	0.8978	0.839	0.9081	0.9333	0.8403	0.8103	0.822
P value	0.3179	0.0972	0.424	0.4811	0.0759	0.0368	0.0363
Passed normality test (alpha=0.05)?	Yes	Yes	Yes	Yes	Yes	No	No
P value summary	ns	ns	ns	ns	ns	*	*

	Imp Regularity						
	Control			300µM Imipramine			
	1338520	1118990	965499	1287.15	86965.7	748426	523926
	1110430	893016	2345810	30035.6	352457	779896	242737
	478228	2910440	2020660	5878.5	10362.1	18152	158084
	870442	1986860	2472680		1462460	400156	108966
	1701110	2009350	1254070		238111	105941	5024.35
	2355020	2245880	290171				13350.8
	1934770	2947750					
Number of values	7	7	6	3	5	5	6
Minimum	478228	893016	290171	1287	10362	18152	5024
Maximum	2355020	2947750	2472680	30036	1462460	779896	523926
Range	1876792	2054734	2182509	28748	1452098	761744	518902
Mean	1398360	2016041	1558148	12400	430071	410514	175348
Std. Deviation	646325	794527	862805	15444	592152	352650	192876
Std. Error of Mean	244288	300303	352239	8917	264818	157710	78741
Shapiro-Wilk test							
W	0.9901	0.9131	0.9279	0.8663	0.7552	0.8793	0.8704
P value	0.9934	0.4179	0.5638	0.2849	0.0332	0.306	0.2278
Passed normality test (alpha=0.05)?	Yes	Yes	Yes	Yes	No	Yes	Yes
P value summary	ns	ns	ns	ns	*	ns	ns

Imp Density		Imp Regularity	
Mann Whitney test		Mann Whitney test	
P value	4.8E-14	P value	3.52E-08
Exact or approximate P value?	Exact	Exact or approximate P value?	Exact
P value summary	****	P value summary	****
Significantly different (P < 0.05)?	Yes	Significantly different (P < 0.05)?	Yes
One- or two-tailed P value?	Two-tailed	One- or two-tailed P value?	Two-tailed
Sum of ranks in column A,B	906 , 634	Sum of ranks in column A,B	573 , 207
Mann-Whitney U	4	Mann-Whitney U	17
Difference between medians		Difference between medians	
Median of column A	17.19, n=20	Median of column A	1817940, n=20
Median of column B	3.349, n=35	Median of column B	108966, n=19
Difference: Actual	-13.84	Difference: Actual	-1708974
Difference: Hodges-Lehmann	-13.22	Difference: Hodges-Lehmann	-1333069

supplementary figure 6F

beta-tubulin quantification

	Control			300 µM Imipramine		
	24.32599	14.5881	22.90001	35.25027	12.55235	30.56879
	28.51212	24.86636	27.73355	25.73666	27.99775	15.72698
	27.78545	36.82725	18.78778	31.72776	35.94295	32.57402
	30.39652	21.82948	13.25424	25.78118	22.65047	22.78668
	31.8334	43.33243	35.32236	46.73144	30.49294	
			21.59705			
			29.09088			
			33.12645			
			20.66884			
			29.13299			
			12.05722			
			17.16816			
Number of values	5	5	12	5	5	4
Minimum	24.33	14.59	12.06	25.74	12.55	15.73
Maximum	31.83	43.33	35.32	46.73	35.94	32.57
Range	7.507	28.74	23.27	20.99	23.39	16.85
Mean	28.57	28.29	23.4	33.05	25.93	25.41
Std. Deviation	2.856	11.62	7.528	8.662	8.875	7.715
Std. Error of Mean	1.277	5.198	2.173	3.874	3.969	3.858
Shapiro-Wilk test						
W	0.9683	0.9557	0.9613	0.8788	0.9653	0.9279
P value	0.8645	0.7775	0.8019	0.3039	0.844	0.5823
Passed normality test (alpha=0.05)?	Yes	Yes	Yes	Yes	Yes	Yes
P value summary	ns	ns	ns	ns	ns	ns

Column B	300 µM Imipramine
vs.	vs.
Column A	Control
Mann Whitney test	
P value	0.3268
Exact or approximate P value?	Exact
P value summary	ns
Significantly different (P < 0.05)?	No
One- or two-tailed P value?	Two-tailed
Sum of ranks in column A,B	376 , 290
Mann-Whitney U	123
Difference between medians	
Median of column A	26.30, n=22
Median of column B	29.25, n=14
Difference: Actual	2.945
Difference: Hodges-Lehmann	2.733

supplementary figure 7A

Phaloidin intensity						
	DMSO			1μM CytoD		
	22305.21	23779.43	24272.29	12072.87	8579.328	11202.45
	20103.96	24862.69	27112.08	8763.022	6687.103	12559.92
	22770.16	20301.1	24464.88	8850.448	8636.384	16860.3
	33393.18	22990.48		8848.423	7815.74	17175.03
				8062.481	7997.113	17314.96
						11766.01
						10558.03
Number of values	4	4	3	5	5	7
Minimum	20104	20301	24272	8062	6687	10558
Maximum	33393	24863	27112	12073	8636	17315
Range	13289	4562	2840	4010	1949	6757
Mean	24643	22983	25283	9319	7943	13920
Std. Deviation	5948	1946	1587	1574	787.7	3054
Std. Error of Mean	2974	973	916.2	704	352.3	1154
Shapiro-Wilk test						
W	0.7993	0.9391	0.8006	0.7281	0.8823	0.8118
P value	0.1009	0.6489	0.116	0.0184	0.32	0.0535
Passed normality test (alpha=0.05)?	Yes	Yes	Yes	No	Yes	Yes
P value summary	ns	ns	ns	*	ns	ns

Table Analyzed	Phaloidin intensity
Column B	1μM CytoD
vs.	vs.
Column A	DMSO
Mann Whitney test	
P value	9.31E-08
Exact or approximate P value?	Exact
P value summary	****
Significantly different (P < 0.05)?	Yes
One- or two-tailed P value?	Two-tailed
Sum of ranks in column A,B	253 , 153
Mann-Whitney U	0
Difference between medians	
Median of column A	23779, n=11
Median of column B	8850, n=17
Difference: Actual	-14929
Difference: Hodges-Lehmann	-13669

tubulin area

	DMSO			1µM CytoD		
	28.95015	17.81666	27.44238	28.26758	18.03978	22.75348
	35.87509	23.54572	18.96213	17.77882	15.78972	36.14065
	16.60849	14.29522	38.92787	37.90992	17.01627	25.33621
	39.43782	20.76478	36.3831	41.35732	17.04923	17.34555
			30.00885	45.34806	21.42641	21.9233
				31.93469		
Number of values	4	4	5	6	5	5
Minimum	16.61	14.3	18.96	17.78	15.79	17.35
Maximum	39.44	23.55	38.93	45.35	21.43	36.14
Range	22.83	9.251	19.97	27.57	5.637	18.8
Mean	30.22	19.11	30.34	33.77	17.86	24.7
Std. Deviation	10.06	3.969	7.878	9.981	2.145	7.016
Std. Error of Mean	5.032	1.985	3.523	4.075	0.9593	3.138
Shapiro-Wilk test						
W	0.9335	0.9921	0.9589	0.9653	0.8664	0.9006
P value	0.6154	0.9678	0.8001	0.8595	0.2521	0.4131
Passed normality test (alpha=0.05)?	Yes	Yes	Yes	Yes	Yes	Yes
P value summary	ns	ns	ns	ns	ns	ns

Table Analyzed	tubulin area_nested	
Column A	DMSO	
vs.	vs.	
Column B	1µM CytoD	
Nested t test		
P value	0.865473	
P value summary	ns	
Significantly different (P < 0.05)?	No	
One- or two-tailed P value?	Two-tailed	
t, df	t=0.1806, df=4	
F, DFn, Dfd	0.03261, 1, 4	
How big is the difference?		
Mean of column A	26.62	
Mean of column B	25.55	
Difference between means (A - B) :	1.077 ± 5.966	
95% confidence interval	-15.49 to 17.64	
Random effects	SD	Variance
Variation within subcolumns	7.55	57
Variation among subcolumn means	6.433	41.38

Do the subcolumns differ (within each column)?	
Chi-square, df	5.556, 1
P value	0.018422
P value summary	*
Is there significant difference between subcolumns (P < 0.05)?	Yes
Data analyzed	
Number of treatments (columns)	2
Number of subjects (subcolumns)	6
Total number of values	29

supplementary figure 7B

beta1 density

DMSO			CytoD		
13.286	16.418	20.892	21.076	15.04	18.88
19.086	14.56	21.146	18.784	20.78	18.33
15.84	14.954	17.198	17.348	13.6725	22.238
	21.854	19.592		16.546	22.3975
		20.2167		15.454	21.9375
					21.9375
					16.6725
					17.878

beta1 regularity

DMSO			CytoD		
1903067	649293	468947	1458002	484457	392220
1538361	298337	500612	2250298	224223	230185
1591360	352530	240109	739651	334254.8	283432
	154665	244511		194140.2	321368
		208873		340325.8	334161
					334161
					1065949
					381675

beta2 density

DMSO			CytoD		
N1	N2	N3	N1	N2	N3
16.498	14.28	16.374	15.386	14.186	18.094
14.946	18.632	15	18.7567	15.814	18.735
15.296	16.174	16.88	17.024	17.348	19.402
		21.682		18.414	
				11.956	

beta2 regularity

DMSO			CytoD		
258655	143705	265906	906864	381860	221696
271047	141304	96367.3	927801	790949	124724
281417	173053	224191	1708160	903929	274403
				511875	
				352120	

Table Analyzed	beta1 density	
Column A	Control	
vs.	vs.	
Column B	CytoD	
Nested t test		
P value	0.66204	
P value summary	ns	
Significantly different (P < 0.05)?	No	
One- or two-tailed P value?	Two-tailed	
t, df	t=0.4712, df=4	
F, DFn, Dfd	0.2220, 1, 4	
How big is the difference?		
Mean of column A	17.72	
Mean of column B	18.5	
Difference between means (A - B) ± SEM	-0.7802 ± 1.656	
95% confidence interval	-5.377 to 3.817	
Random effects	SD	Variance
Variation within subcolumns	2.469	6.096
Variation among subcolumn means	1.641	2.693
Do the subcolumns differ (within each column)?		
Chi-square, df	3.109, 1	
P value	0.07788	
P value summary	ns	
Is there significant difference between subcolumns (P < 0.05)?	No	
Data analyzed		
Number of treatments (columns)	2	
Number of subjects (subcolumns)	6	
Total number of values	28	

Table Analyzed	beta1 regularity	
Column A	Control	
vs.	vs.	
Column B	CytoD	
Nested t test		
P value	0.92928	
P value summary	ns	
Significantly different (P < 0.05)?	No	
One- or two-tailed P value?	Two-tailed	
t, df	t=0.09447, df=4	
F, DFn, Dfd	0.008925, 1, 4	
How big is the difference?		
Mean of column A	785481	
Mean of column B	731236	
Difference between means (A - B) ± SEM	54245 ± 574200	
95% confidence interval	-1539990 to 1648480	
Random effects	SD	Variance
Variation within subcolumns	300161	9E+10
Variation among subcolumn means	687747	4.7E+11
Do the subcolumns differ (within each column)?		
Chi-square, df	22.28, 1	
P value	2.4E-06	
P value summary	****	
Is there significant difference between subcolumns (P < 0.05)?	Yes	
Data analyzed		
Number of treatments (columns)	2	
Number of subjects (subcolumns)	6	
Total number of values	28	

Table Analyzed	beta2 density	
Column A	Control	
vs.	vs.	
Column B	CytoD	
Nested t test		
P value	0.74959	
P value summary	ns	
Significantly different (P < 0.05)?	No	
One- or two-tailed P value?	Two-tailed	
t, df	t=0.3420, df=4	
F, DFn, Dfd	0.1169, 1, 4	
How big is the difference?		
Mean of column A	16.54	
Mean of column B	16.93	
Difference between means (A - B) ± SEM	-0.3887 ± 1.137	
95% confidence interval	-3.545 to 2.767	
Random effects	SD	Variance
Variation within subcolumns	2.129	4.532
Variation among subcolumn means	0.7893	0.623
Do the subcolumns differ (within each column)?		
Chi-square, df	0.3069, 1	
P value	0.57958	
P value summary	ns	
Is there significant difference between subcolumns (P < 0.05)?	No	
Data analyzed		
Number of treatments (columns)	2	
Number of subjects (subcolumns)	6	
Total number of values	21	

Table Analyzed	beta2 regularity	
Column A	Control	
vs.	vs.	
Column B	CytoD	
Nested t test		
P value	0.18604	
P value summary	ns	
Significantly different (P < 0.05)?	No	
One- or two-tailed P value?	Two-tailed	
t, df	t=1.595, df=4	
F, DFn, Dfd	2.543, 1, 4	
How big is the difference?		
Mean of column A	206183	
Mean of column B	657350	
Difference between means (A - B) ± SEM	-451168 ± 282947	
95% confidence interval	-1236753 to 334418	
Random effects	SD	Variance
Variation within subcolumns	222301	4.9E+10
Variation among subcolumn means	323659	1E+11
Do the subcolumns differ (within each column)?		
Chi-square, df	7.753, 1	
P value	0.00536	
P value summary	**	
Is there significant difference between subcolumns (P < 0.05)?	Yes	
Data analyzed		
Number of treatments (columns)	2	
Number of subjects (subcolumns)	6	
Total number of values	20	

supplementary figure 7C

FRET														
	beta1								beta2					
	DMSO				CytoD				DMSO			CytoD		
	10.96418	48.18749	64.52479		22.2674	2.31787	63.0174	34.1475	40.4759	11.2857	2.987154	38.0647	47.3931	48.50867
	24.67564	17.96851	88.59135		16.0437	14.1774	45.7913	44.9092	27.2587	11.2857	48.11992		63.8831	60.51268
	29.6755	18.21963	41.29518		1.16453		37.0139	31.6592	38.8688		7.63208		45.3701	46.23913
	33.31921	31.66944	49.62085		15.924		69.2542	48.1618	42.8674		55.32427		31.9674	33.68167
	5.464664	43.18685	43.18675		25.5109		38.6962	85.9991	53.4583		65.54874		72.7616	
	26.30262	53.36961	54.51552		16.0124		46.1936	58.1103	36.0601		21.04288		14.4643	
	37.75959	29.47386	39.73797		5.4713				43.7188				14.75	
	23.55088	20.26665	41.83722		16.3057				47.7072					
	35.83938		42.69865											
	30.86381		51.90657											
	26.90075		46.18309											
	39.15704		23.61496											
Number of values	12	8	12		8	2	6	6	8	2	6	1	7	4
Minimum	5.465	17.97	23.61		1.165	2.318	37.01	31.66	27.26	11.29	2.987	38.06	14.46	33.68
Maximum	39.16	53.37	88.59		25.51	14.18	69.25	86	53.46	11.29	65.55	38.06	72.76	60.51
Range	33.69	35.4	64.98		24.35	11.86	32.24	54.34	26.2	0	62.56	0	58.3	26.83
Mean	27.04	32.79	48.98		14.84	8.248	49.99	50.5	41.3	11.29	33.44	38.06	41.51	47.24
Std. Deviation	10.19	13.99	15.87		8.018	8.386	13.18	19.89	7.823	0	26.35	0	22.6	10.99
Std. Error of Mean	2.942	4.946	4.581		2.835	5.93	5.381	8.118	2.766	0	10.76	0	8.543	5.497
Shapiro-Wilk test														
W	0.9029	0.8962	0.8755		0.899	N too sm.	0.8763	0.8902	0.983	N too sm.	0.9032	N too sm.	0.9299	0.9765
P value	0.173	0.2668	0.0767		0.2828		0.2526	0.319	0.9762		0.3931		0.5503	0.8815
Passed normality test (alpha=0.05)?	Yes	Yes	Yes		Yes		Yes	Yes	Yes		Yes		Yes	Yes
P value summary	ns	ns	ns		ns		ns	ns	ns		ns		ns	ns
Sidak's multiple comparisons test	Predicted (LS) mean difl	95.00% CI of diff.	Significant?	Summary	Adjusted P Value									
DMSO - CytoD														
β_1AR	6.41	-5.541 to 18.36	No	ns	0.39912									
β_2AR	-8.53	-24.54 to 7.481	No	ns	0.4039									
Test details	Predicted (LS) mean 1	Predicted (LS) mean 2	Predicted (LS) mean diff.	SE of dif	N1	N2	t	DF						
DMSO - CytoD														
β_1AR	36.7	30.29	6.41	5.238	32	20	1.224	76						
β_2AR	34.6	43.13	-8.53	7.018	16	12	1.216	76						
