# Supplementary material for: Microtubule-Mediated Regulation of β2AR Translation and Function in Failing Hearts
Source: Circ Res. 2023 Oct 23;133(11):944–58. doi: 10.1161/CIRCRESAHA.123.323174 (PMC10635332; doi:10.1161/CIRCRESAHA.123.323174)
Supplement: Supplementary file 3 [file res-133-944-s003.pdf]

Major Resources Table

In order to allow validation and replication of experiments, all essential research materials listed in the Methods should be included in the Major Resources Table below. Authors are encouraged to use public repositories for protocols, data, code, and other materials and provide persistent identifiers and/or links to repositories when available. Authors may add or delete rows as needed.

Animals (in vivo studies)

Species	Vendor or Source	Background Strain	Sex	Persistent ID / URL
Rattus Norvegicus	Charles River	Sprague-Dawley	Male	https://www.criver.com/

Antibodies

Target antigen	Vendor or Source	Catalog #	Working concentration	Lot # (preferred but not required)	Persistent ID / URL
β-tubulin (clone TU27)	BioLegend	903401	1:1000		https://www.biolegend.com/fr-ch/products/purified-anti-beta[illegible]<br>tubulin-antibody-11242?GroupID=BLG14147
β1 adrenergic receptor	Alomone Labs	AAR-023	1:100		https://www.alomone.com/p/anti-1-adrenoceptor-extracellular/AAR-023
β2 adrenergic receptor	Alomone Labs	AAR-016	1:100		https://www.alomone.com/p/anti-2-adrenoceptor-extracellular/AAR-016
Di-8-ANEPPS	Invitrogen	D3167	10 µM		https://www.thermofisher.com/order/catalog/product/D3167
Alexa Fluor 488 donkey anti-mouse	Thermo Fisher Scientific	A21202	1:1000		https://www.thermofisher.com/antibody/product/Donkey-anti-Mouse-IgG-H-L-Highly-Cross-Adsorbed-Secondary-Antibody-Polyclonal/A-21202
Alexa Fluor 546 donkey anti-mouse	Thermo Fisher Scientific	A10036	1:1000		https://www.thermofisher.com/antibody/product/Donkey-anti-Mouse-IgG-H-L-Highly-Cross-Adsorbed-Secondary-Antibody-Polyclonal/A10036
Alexa Fluor 488 goat anti-rabbit	Thermo Fisher Scientific	A11008	1:1000		https://www.thermofisher.com/antibody/product/Goat-anti-Rabbit-IgG-H-L-Cross-Adsorbed-Secondary-Antibody-Polyclonal/A-11008
Alexa Fluor 546 donkey anti-rabbit	Thermo Fisher Scientific	A10040	1:1000		https://www.thermofisher.com/antibody/product/Donkey-anti-Rabbit-IgG-H-L-Highly-Cross-Adsorbed-Secondary-Antibody-Polyclonal/A10040
Alexa Fluor™ 546 Phalloidin	Invitrogen	A22283	1:600		https://www.thermofisher.com/order/catalog/product/A2228[illegible]

DOI [to be added]

Data & Code Availability

Description	Source / Repository	Persistent ID / URL
Fiji macro for PI_DI analysis	In-house	In supplementary materials
Fiji macro for distance analysis	In-house	In supplementary materials

Other

Description	Source / Repository	Persistent ID / URL
RNAscope™ Multiplex Fluorescent Reagent Kit v2	Advanced Cell Diagnostics	https://acdbio.com/rnascope-multiplex-fluorescent-v2-assay
RNA-Protein Co-detection Ancillary Kit	Advanced Cell Diagnostics	https://acdbio.com/co-detection-of-mrna-and-protein
Laminin	Bio-Techne	https://www.bio-techne.com/p/cell-culture/cultrex-3-d-culture-matrix-laminin-i_3446-005-01
Isoproteranol	Sigma-Aldrich	https://www.sigmaaldrich.com/GB/en/product/sigma/i6504
ICI 118.551	Tocris	https://www.tocris.com/products/ici-118-551-hydrochloride_0821
CGP-20712A	Sigma-Aldrich	https://www.sigmaaldrich.com/GB/en/product/sigma/c231
IBMX	Sigma-Aldrich	https://www.sigmaaldrich.com/GB/en/product/sigma/i5879
Forskolin	Tocris	https://www.tocris.com/products/forskolin_1099
Vinblastine	Tocris	https://www.tocris.com/products/vinblastine-sulfate_1256
Imipramine	Sigma-Aldrich	https://www.sigmaaldrich.com/GB/en/product/cerillian/i902
Nocodazole	Tocris	https://www.tocris.com/products/nocodazole_1228
Cytochalasin D	Tocris	https://www.tocris.com/products/cytochalasin-d_1233

DOI [to be added]

ARRIVE GUIDELINES

The ARRIVE guidelines (https://arriveguidelines.org/) are a checklist of recommendations to improve the reporting of research involving animals. Key elements of the study design should be included below to better enable readers to scrutinize the research adequately, evaluate its methodological rigor, and reproduce the methods or findings.

Study Design

Groups	Sex	Age	Number (prior to experiment)	Number (after termination)	Littermates (Yes/No)	Other description
Group 1 (Control)	M	16-28 weeks	36	53	No	
Group 2 (MI)	M	24-28 weeks	12	17	No	
Add more if needed						

Sample Size: Please explain how the sample size was decided Please provide details of any a *prior* sample size calculation, if done.

- A minimum of 3 animals were used for initial experiment and analysis. If a trend is observed but the dataset failed to give statistical significance, 2-3 more animals were used.

Inclusion Criteria

- In all experiments, cells with healthy morphology (rod shape, non-apoptotic) were included.

Exclusion Criteria

1. smFISH: dead cells or cells with ill morphology (did not have rod shape morphology) were excluded.
2. Immunostaining: dead cells or cells with ill morphology (did not have rod shape morphology) were excluded. Cells with poor fluorescent labelling were also excluded.
3. Nanoscale subcellular biopsy: dead cells were excluded from the dish.
4. RNA-protein co-detection: cells with ill morphology (did not give rod shape morphology)
5. FRET microscopy: if the cells are dead, or if the cell failed to respond to saturator

Randomization

- Animals arrived at the Central Biomedical Services animal unit were randomly selected for coronary ligation surgery for heart failure model.

Blinding

- Experiments involving imaging were blinded. Samples are alphabetically labelled. The immunostaining experiment, imaging, and image analysis were carried out by at least more than 1 person.

DOI [to be added]
